# Rapid development of clone‐specific, high‐performing perfusion media from established feed supplements

**DOI:** 10.1002/btpr.2933

**Published:** 2019-11-11

**Authors:** Patrick Mayrhofer, David Reinhart, Andreas Castan, Renate Kunert

**Affiliations:** ^1^ Department of Biotechnology University of Natural Resources and Life Sciences (BOKU) Vienna Austria; ^2^ GE Healthcare Bio‐Sciences AB Uppsala Sweden

**Keywords:** CSPR, DoE, multivariate data analysis, regression model, small‐scale perfusion model

## Abstract

Perfusion cultivation of recombinant CHO cells is of substantial interest to the biopharmaceutical industry. This is due to increased space–time‐yields (STYs) and a short residence time of the recombinant protein in the bioreactor. Economic processes rely on cultivation media supporting rapid growth in the exponential phase and high protein production in the stationary phase at minimal media consumption rates. To develop clone‐specific, high‐performing perfusion media we present a straightforward and rapid two‐step approach combining commercially available basal media and feed supplements using design‐of‐experiment. First, the best performing feed supplements are selected in batch cultures. Then, the mixing ratio of selected feed supplements is optimized in small‐scale semicontinuous perfusion cultures. The final media formulation is supported by statistical response surface modeling of a set of cultivation experiments with blended media formulations. Two best performing novel media blends were finally applied to perfusion bioreactor verification runs to reach 200 × 10^6^ c/ml within 2 weeks at minimum cell‐specific perfusion rates as low as 10–30 pL/c/d. Obtained STYs of 0.4–1.2 g/L/d represent a 10‐fold increase compared to batch cultures. This general workflow is universally applicable to any perfusion platform combining a specific cell line, basal medium, and established feed solutions.

## 1 INTRODUCTION

Perfusion processes gained much attraction in the past for reliable production of labile therapeutic biologics including recombinant interferon‐beta, coagulation factor VIII, recombinant follicle stimulating hormone, and different monoclonal antibodies (mAbs).^1^ Viable cell densities (VCDs) of up to 130 × 10^6^ c/ml and high productivities,^2^ as well as space–time yields (STYs)^3^ were reported. Despite high promises of this early technology, perfusion processes were rarely implemented in industry because of more complex process control, higher risk of process failure and higher media demands compared to traditional batch or established fed‐batch processes.^4^

As for any cell cultivation, also for perfusion processes the success depends highly on the available perfusion media. Maintaining high cell‐specific production rates (qP) is a major determinant of the final yield. This is achieved through a balance between the supplied nutrients and the withdrawal of toxic by‐products.

A critical parameter for an economic process that describes the media performance is the cell‐specific perfusion rate (CSPR). Low CSPR are advantageous due to lower volumes during media preparation (thus reduced tank sizes) and higher product concentrations in the harvest. Minimum CSPR as low as 50 to 300 pL/c/d were reported in literature^2^, ^5^ but some groups even achieved 15–20 pL/c/d.^6^, ^7^

Despite numerous cell culture media are commercially available for batch and fed‐batch cultivation, this is not the case for perfusion media, which must be designed individually in a complex and time‐consuming process. Here, we present a straightforward strategy for perfusion media development by screening and optimizing the spike concentration and blending of eight commercially available feed supplements. In a first step, the best feed supplements are identified by spiking the basal media with different feed combinations according to a fractional factorial design. The supplement concentrations were then optimized in a face‐centered central‐composite design using semicontinuous small‐scale (10 ml) perfusion cultures (“pseudo‐perfusion”) in shaking tubes. This two‐step and empirical design‐of‐experiment (DoE) workflow resulted in two novel perfusion media. The final perfusion media were used in bench‐scale bioreactor runs at 500 ml working volume that achieved VCDs of more than 200 × 10^6^ c/ml during a 2 weeks bioprocess. Bioreactor perfusion cultures were successfully operated at CSPRs as low as 10–30 pL/c/d.

## 2 MATERIALS AND METHODS

### 2.1 Cell lines, media, feed supplements, and process analytics

A recombinant CHO K1 cell line producing an IgG1 antibody under the glutamine‐synthetase (GS) selection system was used throughout this study. Cells were passaged every 3–4 days for routine cultivation and adapted to CDM4NS0 or ActiPro basal media. Eight different HyClone™ Cell Boost™ feed supplements, referred to as CB1 to CB7b in this study, were supplemented into basal media and used in “spiked batch” or semicontinuous perfusion cultures in shaking tubes. Feed supplements are chemically defined mixtures of diverse nutrient substance groups as summarized in Table 1 according to the manufacturer.^8^ Cultures were incubated in a Kuhner shaker incubator at 37°C, 80% relative humidity, 220 rpm, 90° angle, and 50 mm orbital shaking diameter.

**Table 1:** Substance classes included in eight commercially available HyClone™ feed supplements (“Cell Boosts,” CB1‐7b) as disclosed by the manufacturer^8^

Feed supplement	Amino acids	Vitamins	Glucose	Trace elements	Growth factors (peptides)	Hypoxanthine / thymidine	Lipids	Cholesterol
CB 1	●	●	●					
CB 2	●		●					
CB 3	●	●	●	●		●		
CB 4	●	●	●	●	●		●	●
CB 5	●	●	●	●	●	●	●	●
CB 6	●	●	●	●	●	●	●	●
CB 7a	●	●	●	●				
CB 7b	●							

Cell concentration, viability, and cell size was measured with a Vi‐Cell XR. Glucose, lactate, glutamine, glutamate, and ammonia concentrations were quantified using a BioProfile 100 Plus. Osmolality was analyzed with Osmomat 030 and product concentrations were determined using an OctetRed.

### 2.2 Screening design‐of‐experiment in spiked batch cultures (DoE1)

**Figure 1:**
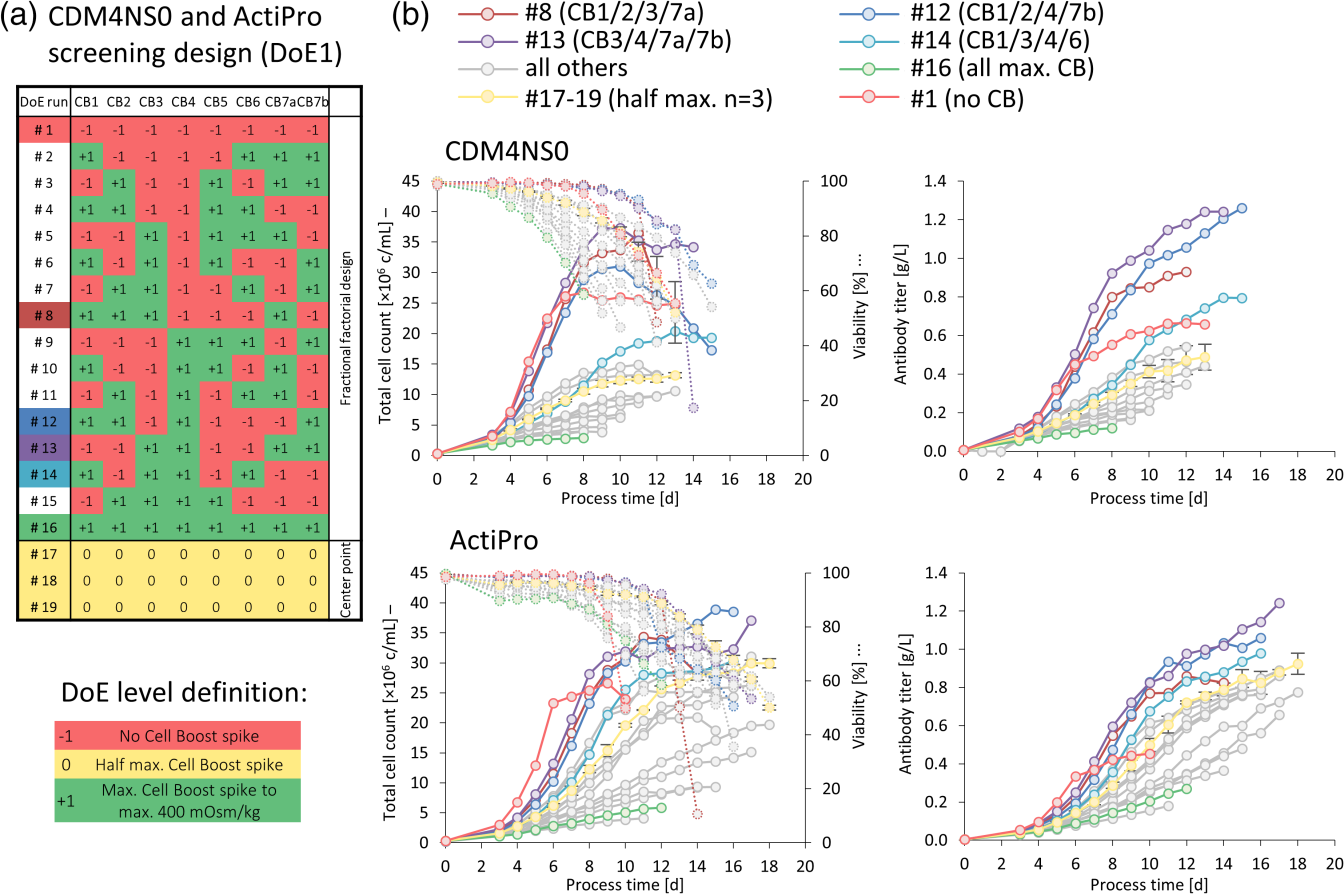
Screening design (DoE1) of eight different feed supplements (CB1‐7b) in spiked batch cultures in CDM4NS0 and ActiPro. A DoE matrix was used to test 19 different feed combinations (a). Cell culture data including total cell count, viability, and antibody titer are exemplarily shown (b). Error bars represent the *SD* of the triplicate (*n* = 3) cultures (#17–19). The full data set used for subsequent regression analyses can be found in Supporting Information S1 and S2

A screening experimental design (Figure 1a) was used to identify the main effects of eight different feed supplements representing continuous variables (“factors”) at three concentrations (“factor levels”) on cell culture parameters (“responses”). A total of 19 spiked batch cultures were used to establish a fractional factorial design at two levels to resolve linear (main) effects (run #1 to #16) and a triplicate center point to detect nonlinear curvature in the response surface (run #17 to #19). Three different factor levels were defined for each feed supplement. For definition of the maximum factor Level +1 all feed supplements were mixed according to their total molar amino acid concentration and spiked into CDM4NS0 or ActiPro basal medium to reach a final target osmolality of 400 mOsm/kg. Accordingly, in the first DoE1 screening design, CB 1 to 6, 7a, and 7b were spiked into CDM4NS0 and ActiPro at 7.2, 4.3, 13.0, 4.1, 11.4, 11.1, 2.5, and 0.3%, respectively. DoE factor Level −1 was defined as no supplement addition and factor Level 0 as the half‐maximum spike concentration. Media were prepared by spiking the basal medium with feed supplements according to the DoE matrix depicted in Figure 1a. Spiked batch experiments were seeded at 0.3 × 10^6^ c/ml at a working volume of 30 ml in 50 ml shaking tubes. Once glucose levels reached 3 g/L in spiked batch cultures a 250 g/L glucose stock solution was added to increase glucose levels to 6 g/L. A 60% viability threshold was defined as batch termination criterion.

### 2.3 Semicontinuous small‐scale models (“pseudo‐perfusion”) in shaking tubes

Ten milliliters small‐scale semicontinuous perfusion models were seeded at 10 × 10^6^ c/ml in fresh medium in 50 ml shaking tubes. A daily, complete medium exchange was performed every 24 hr by daily sampling followed by centrifugation (300 g, 7 min) and resuspension of the cell pellet in 10 ml of fresh medium (one reactor‐volume change per day, vvd).

### 2.4 Optimization design‐of‐experiment in semicontinuous perfusion cultures (DoE2)

An optimization experiment was used to resolve linear (main), nonlinear (quadratic), and interaction effects of the preselected feed supplements in a semicontinuous perfusion mode. The fractional factorial design at two levels with a triplicate center point was expanded with star points to create a central‐composite face‐centered design (Figure 3a,c). Factor Level +1 for each feed supplement was again defined as the maximum spike concentration to reach a maximum target osmolality of 400 mOsm/kg when all preselected feed supplements were mixed according to their total amino acid ratio and spiked into the basal medium. CDM4NS0 was spiked with CB 1, 3, 7a, and 7b at 11.1, 19.9, 3.8, and 0.8%, respectively. ActiPro was spiked with CB1, 3, and 7b at 13.9, 25.1, and 0.5%, respectively. Differently spiked media at three factor levels were subsequently prepared according to Figure 3a,c.

### 2.5 Data analysis and regression models

Statistical analyses of the two DoE runs were performed with the GraphPad Prism 6 software for Pearson correlation analysis or the MODDE 12.1 software for response surface modeling. Mean, minimum, and maximum values of different cell culture parameters describing growth ((p)VCD: (peak) viable cell density, μ: specific growth rate, ∆IVCD: daily cell days, IVCD: cumulative viable cell days), productivity ((p)Titer: (peak) titer, qP: specific production rate), metabolic activity (specific qGluc‐ and qGlu consumption or qLac‐, qGln‐, and qNH4+ production), or cell morphology (cell size) were summarized for the exponential phase (average of Day 0–4 in semicontinuous perfusion cultures) and stationary phase (average of Day 5–11 in semicontinuous perfusion cultures) individually and used as input for statistical evaluation. Measured responses were fit by partial least squares (PLS) models. For the fractional factorial screening design at resolution III in DoE1 only linear (main) coefficients were used, whereas for the face‐centered central‐composite design in DoE2 also quadratic terms and interactions were included to fit the response values to the three supplement levels −1, 0, and +1. Model fit (*R* ^2^) and future prediction precision (*Q* ^2^) are the most critical regression model quality parameters and were improved by transforming selected responses or by removing small and nonsignificant model coefficients.

### 2.6 Bioreactor verification runs

A Wave 20/50 system equipped with a Cellbag bioreactor was seeded at 1 × 10^6^ c/ml in fresh nonspiked basal media and operated at 37°C, pH set point 7.0, 24 rpm, and rocking angle of 9°. Once cultures reached 10 × 10^6^ c/ml, perfusion was started on Day 4 at constant volumetric perfusion rate of 1 reactor‐volume change per day (vvd) in CDM4NS0 or on Day 3 with a constant CSPR of 30 pL/c/d in ActiPro that was subsequently reduced to 14 pL/c/d using the final perfusion media formulation. Feed and harvest flow were controlled gravimetrically and pH was adjusted by CO_2_ or 8% sodium bicarbonate. Product quality defined by size‐, charge‐, and glycan profiles was analyzed as reported in Reinhart et al.^9^

## 3 RESULTS AND DISCUSSION

In this study, two different basal media (ActiPro and CDM4NS0) and eight feed supplements (Table 1) were used to develop two novel perfusion culture media. In a first DoE experiment, cultures in batch mode were used to empirically select the best performing feed supplements. In the second DoE experiment, semicontinuous perfusion cultures were employed to establish valid regression models by response surface modeling and to define the optimal mixing ratios. Two novel perfusion media were compared to the corresponding basal media in small‐scale models and finally applied to perfusion bioreactor runs with the goal to reach high VCD of more than 200 × 10^6^ c/ml at low CSPR.

### 3.1 Screening design of feed‐spiked batch cultures (DoE1)

**Figure 2:**
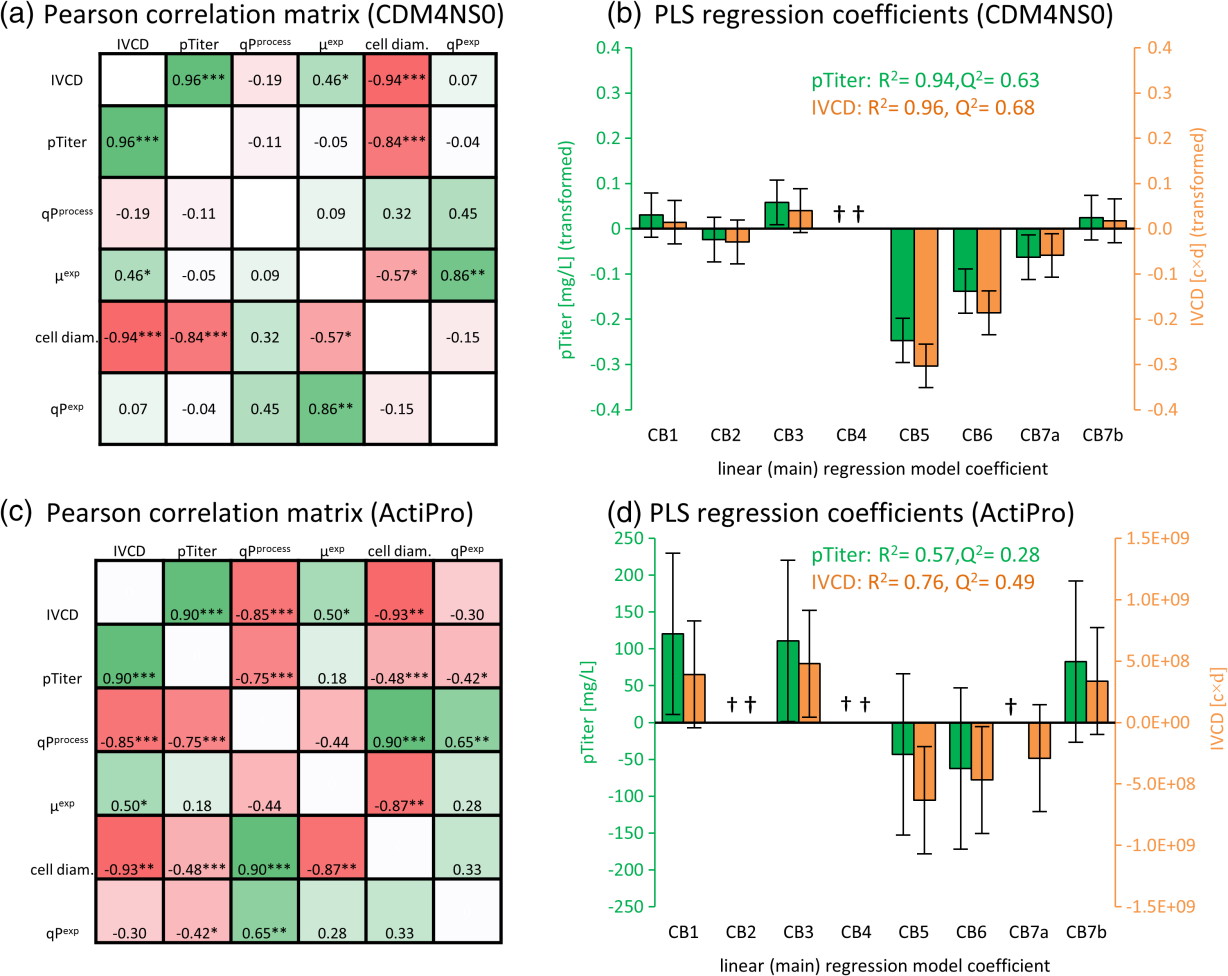
Statistical analyses of spiked batch cultures (DoE1) by Pearson correlation and regression analysis. Pearson correlation coefficient (*r*) and *p*‐values (*<.05, **<.01, ***<.001) were used to detect positive (green) or negative (red) correlations between culture parameters of spiked batch cultures and are exemplarily shown for CDM4NS0 (a) or ActiPro (c). Culture parameters for the exponential phase or the entire process are indicated by the superscript “exp” or “process,” respectively. Linear (main) model coefficients of individual feed supplements are exemplarily shown for peak titer (pTiter) and integrated viable cell density (IVCD) in CDM4NS0 (b) or ActiPro (d). High quality models indicated by *R* ^2^ and *Q* ^2^ by partial least squares (PLS) regression analyses were generated by removing small and nonsignificant coefficients (†). The complete data set can be found in the Supporting Information S3–S5

A fractional factorial design (Figure 1a) was used for the initial screening approach to select those supplement combinations that supported the highest IVCD and peak titers (pTiters) in spiked batch cultures. The pVCD values ranged from 3 to 39 × 10^6^ c/ml in CDM4NS0 or ActiPro basal media due to the various supplement combinations (Figure 1b). pTiters reached 0.1–1.3 g/L and STYs between 0.01 and 0.10 g/L/d (Supporting Information S2). Three individual feed combinations (DoE1 run #8, #12, and #13) in both basal media were particularly interesting as the final IVCD was increased by 85% (3.9–5.6 × 10^9^ c × d) and peak titers by 170% (0.9–1.3 g/L; Supporting Information S2). Interestingly, these cultures contained neither supplement CB5 nor CB6, indicating a negative impact of these two feed solutions for the investigated clone. Early exponential cell growth was highest in both basal media without any feed supplementation. Supplementation of all eight feed solutions at maximum levels critically diminished cell growth accompanied by a fast drop in viability (run #16; Figure 1b). These results emphasized that individual substances of one or multiple feed supplements, may be responsible for the reduced cell growth and was therefore investigated in more detail by correlation and regression analyses (Figure 2).

Pearson correlation analysis indicated that high specific productivity (qP^exp^) directly correlated with high specific growth (μ^exp^) in the exponential phase (Figure 2a,c). It can be concluded that the media supporting high growth also support high productivity at early culture times. Significant positive Pearson correlation coefficients between pTiter and IVCD indicate that cultures accumulate high titers because of increased biomass and not qP. High titers were additionally supported by longer process durations (Supporting Information S3). A significantly negative correlation between feed concentration and pVCD or μ was observed for CB5 and CB6 confirming the observed cell growth suppression (Supporting Information S3).

DoE methodology was subsequently combined with response surface modeling to calculate the linear (main) coefficients for each individual feed supplement (Figure 2b,d). Good model qualities (*R* ^2^ and *Q* ^2^) were obtained by removing small and nonsignificant coefficients for CB2, CB4, and CB7a (Supporting Information S4). Large negative and significant coefficients for IVCD and pTiter were found for CB5, CB6 (Figure 2b,d) that were absent in the best performing cultures #8, #12, and #13. Both feed supplements also decreased the maximum specific growth rate in the exponential phase (max. μ^exp^) and peak cell count (pCC) in both media and reduced process duration in CDM4NS0 (Supporting Information S5). CB7a significantly reduced IVCD and pTiter in CDM4NS0 but not in ActiPro (Figure 2b,d). In contrast, CB3 significantly enhanced pTiter in both media and IVCD in ActiPro. Feed supplements CB1 and CB7b showed additional positive effects on IVCD and pTiter in both media.

In summary, CB1, CB3, and CB7b showed clear beneficial effects on growth and final titer in DoE1 batch cultures and were finally chosen for further evaluation in semicontinuous perfusion cultures. CB7a was additionally tested in CDM4NS0 as it was initially developed as a combination feed supplement with CB7b. Beneficial nutrient supplementation was demonstrated for this system in fed‐batch.^9^ With the chosen screening design at resolution III (DoE1) possible synergistic effects and two‐factor interactions cannot be resolved and were further investigated in the subsequent optimization design (DoE2).

### 3.2 Optimization in semicontinuous small‐scale perfusion models (DoE2)

**Figure 3:**
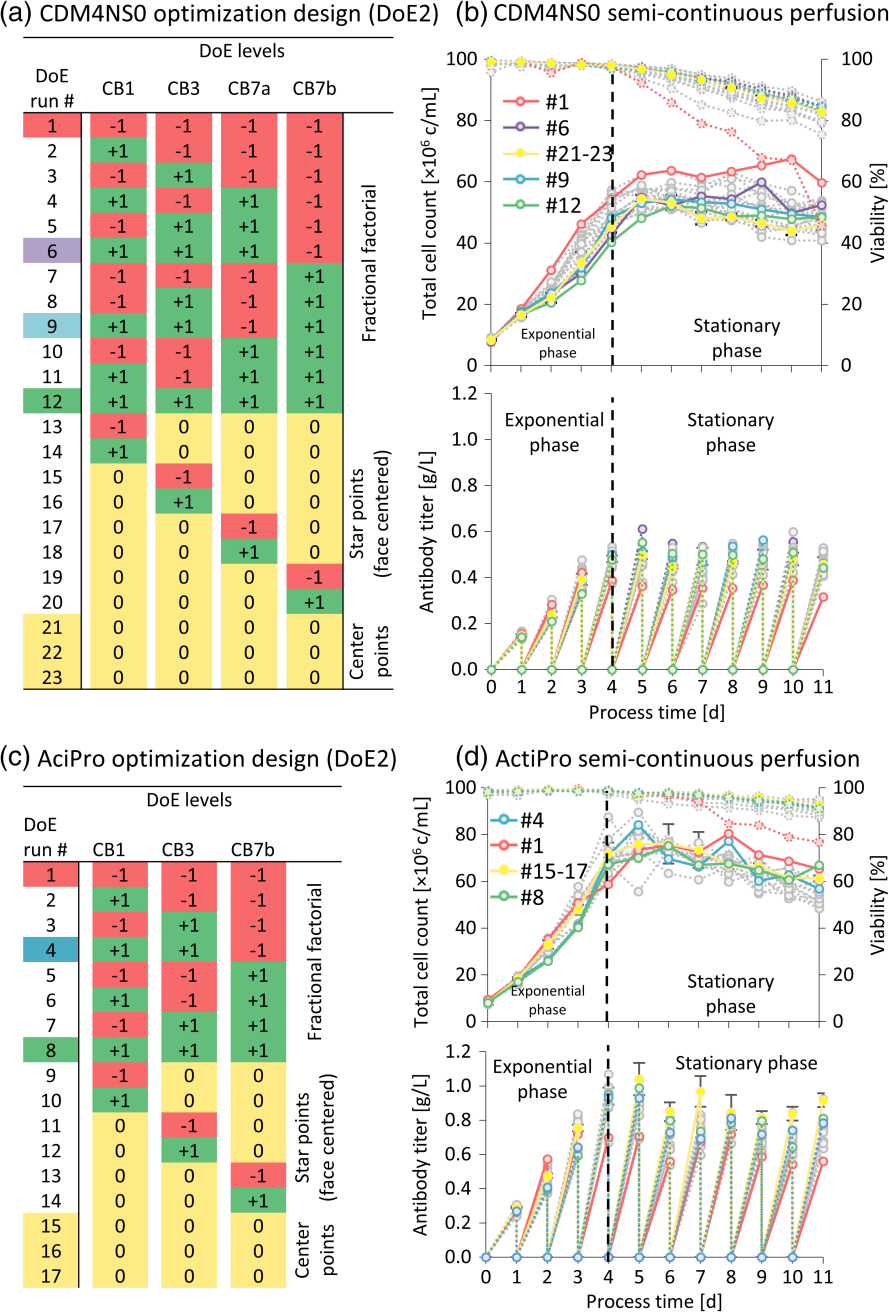
Optimization design (DoE2) for CB1, CB3, CB7a, and CB7b in semicontinuous perfusion models. A DoE matrix for CDM4NS0 (a) or ActiPro (c) was used to generate cell culture data (b and d) for subsequent regression analyses of the exponential‐ and stationary phase. Total cell count, viability, and titer values are exemplarily shown. Error bars represent the *SD* of the triplicate (*n* = 3) cultures (#21–23 for CDM4NS0, #15–17 for ActiPro). The complete data set can be found in Supporting Information S6

A face‐centered central‐composite design was chosen, comprising a total of 23 semicontinuous perfusion runs including cultures for the fractional factorial‐, star‐, and center points (Figure 3a,c). This design allows to fit a quadratic regression model in order to determine linear main effects in addition to two‐factor interaction‐ and nonlinear quadratic effects that explain the slope, synergism, and curvature of the regression model in the response surface, respectively. Daily complete media exchange in a discontinuous mode provided high mean steady‐state VCD values of 42–69 × 10^6^ c/ml for more than 11 days (Figure 3b,d) in both basal CDM4NS0 and ActiPro media supplemented with different feed combinations according to the DoE2 optimization matrix. After an exponential growth phase, this technique enables a stable steady‐state phase from Day 5 to 11, accumulating daily steady‐state mean titers of 0.4–0.6 g/L in CDM4NS0 or 0.6–0.9 g/L in ActiPro (Figure 3b,d). Exceptional cell performance was already observed with the plain basal media in the exponential phase. After day three, the limitation in glucose as primary energy source stopped the specific lactate production followed by a switch to lactate consumption within the 24 hr sampling interval (Supporting Information S6). Residual glutamate and secreted glutamine levels reached critical low concentrations on day four indicated by decreasing viabilities starting in early stationary phase.

In contrast, enrichment of the basal media with all feed supplements at half‐maximum and maximum levels maintained cell viabilities above 80% for 11 days (Figure 3b,d). Similar to batch cultures, any combination of feed supplements decreased cell‐specific growth in the exponential growth phase until day four. However, higher cell‐specific production rates were maintained in the stationary phase from day 5 to 11 (Supporting Information S6). Importantly, although the steady‐state VCDs were also decreased by 1–5% in half‐ and maximum supplemented cultures, mean steady‐state titers were boosted by 30–40% compared to nonsupplemented cultures to 0.5–0.9 g/L. Supplemented cultures also showed highest cell‐specific productivities maintained over the entire culture process duration. Those high‐performing cell cultures never reached glucose limitation and thus enriched highest lactate concentrations. Similarly, glutamate was not limiting and for CDM4NS0 substantial secretion of de novo synthesized l‐glutamine was observed. A healthy cell phenotype can be inferred from the high glutamine levels secreted into the extracellular supernatant because of high activity of the exogenous GS. A profoundly different glutamate/glutamine metabolism was observed in the ActiPro cultures. Lower residual glutamine levels were measured since the basal concentration of the precursor molecule glutamate was generally lower at 400 mg/L. Similarly to lactate, also glutamine secretion was decreased and switched to consumption once glutamate concentrations reached limiting concentrations. Notably, nonspiked ActiPro showed ammonia consumption in the first 3 days of cultures, when glutamate levels were not limiting, but switched to high specific ammonia production once glutamate was limiting due to cleavage of glutamine.

The average stationary qGluc^stat^ consumption and qGlu^stat^ consumption was higher in CDM4NS0, reaching mean steady‐state values of 249 and 11 pg/c/d, respectively. The calculation of the CSPR indicates that VCD^stat^ values of 50 × 10^6^ c/ml at one reactor‐volume change per day (vvd) translates into a CSPR of 20 pL/c/d. This means that a rich perfusion media, fully supporting the cellular nutrient demand in such a process, must supply 12.5 g/L glucose and 550 mg/L glutamate. Glucose and glutamate derived from feed supplements may be seen as surrogate markers for limitation of other media nutrients. As enough glucose and glutamate is supplied by feed solutions it can be expected that also other critical nutrients are supplied in adequate amounts by the predefined feed solutions.

High titers in the exponential phase positively correlated with high cell growth (Supporting Information S8). These fast growing cultures also had higher specific glucose consumption rates leading to lower osmolalities and smaller cell sizes. Cultures with no or low supplement concentrations are indicated by lower starting glucose levels. The provided glucose as energy source is depleted within the 24 hr interval and compensated by catabolism of the previously secreted glutamine indicated by higher specific ammonia production (Supporting Information S6). Such a reciprocal regulation of glucose and glutamine consumption and a shift from glycolysis to glutaminolysis was reported in literature for human fibroblasts,^10^ hybridoma‐,^11^, ^12^ myeloma‐,^13^ and CHO cells.^14^, ^15^ Especially at low glucose concentrations, glutamine plays an essential role as primary energy source.^16^ Martinelle et al^13^ contributed the increase of glutamine consumption under glucose‐limiting conditions to elevated activity of glutaminase and glutamine dehydrogenase.

Specific productivity in the exponential phase at 12.6 ± 0.6 pg/c/d for CDM4NS0 or 20.2 ± 0.9 pg/c/d for ActiPro showed no correlation to any other parameter and was rather similar between cultures with the same basal media. Pearson correlation found that higher stationary qP^stat^ values, but not higher cell numbers, translated into higher daily harvest titers (Supporting Information S8). In CDM4NS0, cultures with higher stationary qP^stat^ showed lower cumulative cell days and all cultures spiked with high CB3 levels also showed high qP^stat^ values, but lower VCD^stat^. No decrease of cell days at high qP^stat^ was observed in ActiPro, but higher specific glucose consumption and lactate production. Unlike in batch screening, the final daily harvested titer in semicontinuous perfusion mode of both media thus showed positive correlation with qP^stat^, but neither with the VCD^stat^ nor with cumulative cell days.

Partial least squares regression was used to assess the impact of individual feed levels on semicontinuous perfusion cultures. For a multitude of parameters good model parameters (*R* ^2^ and *Q* ^2^) were obtained (Supporting Information S9) and key parameters were summarized in Table 2. In contrast to correlation analysis, application of regression analysis allows to quantify the effect of the individual supplements on selected responses. For example, in both basal media systems the daily titer in stationary steady‐state was significantly increased by CB3 showing a linear model coefficient of 23 mg/L in CDM4NS0 (Supporting Information S10A). The linear coefficient is the slope of the regression function and describes the response change when altering the factor from Level 0 to +1. This translates into a modeled main effect of 59 mg/L (Table 2A**)** when changing the CB3 level from −1 (no spike) to +1 (maximum spike) and is in accordance with the actual experimental data (Supporting Information S7A) demonstrating the validity of the established regression models.

**Table 2:** Linear (main) factor effects of semicontinuous perfusion cultures (DoE2) calculated from PLS regression in CDM4NS0 (A) or ActiPro (B)

A. CDM4NS0										
	Model quality (*R* ^2^/*Q* ^2^)		CB1		CB3		CB7a		CB7b	
	Exp.	Stat.	Exp.	Stat.	Exp.	Stat.	Exp.	Stat.	Exp.	Stat.
VCD (×10^6^ c/ml)	0.94/0.86	0.91/0.75	−3.0 ± 1.0	2.5 ± 1.6	−2.7 ± 1.0	−3.7 ± 1.6	−2.8 ± 1.0	1.6 ± 1.6	−1.0 ± 1.0	−1.8 ± 1.5
∆IVCD (×10^6^ c × d/ml)	0.96/0.83	0.93/0.73	−2.3 ± 0.5	2.0 ± 1.2	−1.7 ± 0.5	−4.1 ± 1.2	−1.7 ± 0.5	n.s.	−0.8 ± 0.5	−2.1 ± 1.1
Viability (%)	0.60/0.30	0.93/0.76	n.s.	(~) 0.13 ± 0.03	n.s.	n.s.	n.s.	(~) 0.08 ± 0.03	n.s.	n.s.
Titer (mg/L)	0.94/0.80	0.72/0.57	−24 ± 8	33 ± 27	−30 ± 8	59 ± 26	−21 ± 8	n.s.	n.s.	n.s.
qP (pg/c/d)	0.88/0.61	0.85/0.70	n.s.	(~) n.s.	n.s.	(~) 0.011 ± 0.003	n.s.	(~) n.s.	0.8 ± 0.3	(~) n.s.
Cell diameter (μm)	0.95/0.76	0.99/0.71	0.58 ± 0.15	1.28 ± 0.11	n.s.	0.59 ± 0.12	0.42 ± 0.15	0.77 ± 0.11	n.s.	n.s.
Residual Gluc (mg/L)	0.99/0.81	0.95/0.87	6,041 ± 370	3,153 ± 553	2,870 ± 419	1,713 ± 530	2,296 ± 370	1,557 ± 553	n.s.	n.s.
Residual Lac (mg/L)	0.93/0.70	0.97/0.83	n.s.	1,569 ± 279	n.s.	565 ± 267	417 ± 154	927 ± 279	697 ± 155	371 ± 281
Residual Glu (mg/L)	0.99/0.91	0.89/0.69	46.1 ± 21.7	n.s.	39.8 ± 20.8	n.s.	340.0 ± 21.7	232.4 ± 48.2	105.4 ± 21.9	125.0 ± 50.7
Residual Gln (mg/L)	0.99/0.91	0.99/0.79	15.3 ± 6.4	80.6 ± 19.8	−6.6 ± 6.1	44.4 ± 19.9	139.2 ± 6.4	250.2 ± 19.8	15.3 ± 7.3	25.7 ± 22.4
Residual NH4+ (mg/L)	0.98/0.84	0.90/0.61	8.6 ± 1.6	9.3 ± 4.5	9.0 ± 1.4	n.s	10.6 ± 1.6	10.4 ± 4.5	n.s.	n.s.
qGlucose (pg/c/d)	0.87/0.72	0.93/0.74	−65 ± 20	n.s.	−39 ± 19	35 ± 14	−30 ± 20	n.s.	33 ± 19	n.s.
qLactate (pg/c/d)	0.99/0.91	0.96/0.78	13 ± 5	(~) 0.26 ± 0.05	n.s.	(~) 0.10 ± 0.05	25 ± 5	(~) 0.14 ± 0.05	49 ± 5	(~) 0.11 ± 0.05
IVCD (×10^6^ c × d/ml)	0.93/0.72		n.s.		−41 ± 9		n.s.		−24 ± 11	

B. ActiPro								
	Model quality (*R* ^2^/*Q* ^2^)		CB1		CB3		CB7b	
	Exp.	Stat.	Exp.	Stat.	Exp.	Stat.	Exp.	Stat.
VCD (×10^6^ c/ml)	0.89/0.86	0.74/0.56	−6.9 ± 1.5	n.s.	n.s.	n.s.	n.s.	n.s.
∆IVCD (×10^6^ c × d/ml)	0.88/0.83	0.70/0.47	−4.7 ± 1.1	n.s.	n.s.	n.s.	n.s.	n.s.
Viability (%)	0.68/0.47	0.82/0.68	−0.5 ± 0.3	n.s.	n.s.	(~) 0.2 ± 0.1	n.s.	n.s.
Titer (mg/L)	0.89/0.84	0.79/0.66	−106 ± 23	n.s.	n.s.	100 ± 65	n.s.	n.s.
qP (pg/c/d)	0.54/0.35	0.74/0.57	n.s.	n.s.	n.s.	1.3 ± 0.9	n.s.	n.s.
Cell diameter (μm)	0.92/0.81	0.97/0.76	0.72 ± 0.14	(~) 0.006 ± 0.001	0.36 ± 0.14	(~) 0.004 ± 0.001	n.s.	(~) n.s.
Residual Gluc (mg/L)	0.98/0.94	0.81/0.67	7,438 ± 737	(~) 1.2 ± 0.4	3,618 ± 737	(~) n.s.	n.s.	(~) n.s.
Residual Lac (mg/L)	0.25/0.16	0.96/0.85	320 ± 303	1,672 ± 406	n.s.	1,118 ± 406	n.s.	n.s.
Residual Glu (mg/L)	0.75/0.71	0.83/0.58	144 ± 59	29 ± 18	74 ± 59	n.s.	n.s.	40 ± 18
Residual NH4+ (mg/L)	0.83/0.76	0.86/0.75	(~) 0.17 ± 0.05	(~) n.s.	(~) n.s.	(~) ‐0.04 ± 0.03	(~) n.s.	(~) n.s.
qGlucose (pg/c/d)	0.74/0.69	0.93/0.88	−43 ± 24	27 ± 11	−25 ± 24	20 ± 11	28 ± 24	n.s.
qLactate (pg/c/d)	0.56/0.27	0.92/0.88	25 ± 20	(~) 0.16 ± 0.04	n.s.	(~) 0.09 ± 0.04	n.s.	n.s.

*Note*: Main effects were calculated for the exponential phase (exp.) and stationary phase (stat.). Nonsignificant (n.s.) model terms were excluded from interpretation. Selected responses were transformed (~) for model quality improvement.

Abbreviation: IVCD, integrated viable cell density; PLS, partial least squares; VCD, viable cell densities.

Suppression of cell growth at high viabilities and productivities is especially important when bleed rates in steady‐state perfusion need to be minimized in order to prevent product loss. Such attempts to decouple productivity from growth were already proven by media additives such as small molecule cell growth inhibitors, or altered sodium–potassium levels to arrest cells in the G0/G1 phase.^17^ Alternatively, process control strategies were used to keep cells in a physiological state using lactate consumption and the resulting rise in culture pH to regulate perfusion media addition^18^ or minimizing growth and maximize productivity by reduced culture temperatures^19^, ^20^ or limiting CSPR.^21^ Culture pH, pCO_2_ levels, and osmolality were also shown to profoundly impact cell growth and productivity^22^, ^23^ and may be used for future perfusion control strategies. Indeed, valid regression models confirm the general observation that supplementation with CB1, 3, 7a, or 7b suppresses VCD^exp^ by 1.0–3.0 × 10^6^ c/ml and daily viable cell days (∆IVCD^exp^) by 0.8–2.3 × 10^6^ c × d/ml during exponential phase in CDM4NS0 (Table 2A). Growth was also reduced by CB1 in ActiPro resulting in strong suppression of VCD^exp^ by 6.9 × 10^6^ c/ml and ∆IVCD^exp^ by 4.7 × 10^6^ c × d/ml.

Supplement addition did not substantially alter specific productivities or viabilities in the exponential growth phase. Only a small increase of qP^exp^ with CB7b in CDM4NS0 and a minor reduction of viability with CB1 in ActiPro was induced. As qP^exp^ between nonsupplemented (CDM4NS0: 12 pg/c/d, ActiPro: 20 pg/c/d) and supplemented cultures (CDM4NS0: 12–14 pg/c/d, ActiPro: 18–22 pg/c/d) was highly similar (Supporting Information S7), the resulting titers in supplemented cultures were lower in the exponential phase as a consequence of lowered VCD^exp^.

Regression analysis was also used to quantify the impact of supplement addition on primary metabolic pathways. Because of decreased growth, cell‐specific glucose (qGluc^exp^) uptake was also reduced by CB1, CB3, and CB7a in the exponential phase of both basal media (Table 2). Despite lower qGluc^exp^, specific lactate production (qLac^exp^) was increased with CB1 or CB7a.

In the subsequent CDM4NS0 stationary phase, a positive CB1 main effect on VCD^stat^ by 2.5 × 10^6^ c/ml (Table 2A) was observed accompanied by increased cell days and viabilities. A similar effect was also seen for CB7a, whereas CB3 and CB7b induced reduced stationary VCD^stat^ resulting in lower daily (∆IVCD^stat^) and cumulative cell days (IVCD). A significant positive stationary titer effect of 33 mg/L for CB1 or 59 mg/L for CB3 was modeled in CDM4NS0 but provoked by different routes (Table 2A). CB1 increased stationary VCD^stat^ but not qP^stat^, while CB3 caused increasing qP^stat^ but suppressed VCD^stat^. CB3 similarly enhanced stationary ActiPro titer by 100 mg/L through increased specific productivity qP^stat^ and viability (Table 2B).

Metabolic activity was enhanced by CB3 marked by a higher qGluc^stat^ consumption and higher qLac^stat^ secretion in both media. Part of this energy is channeled into higher production capability indicated by a significant increase in qP^stat^. Beside metabolic changes also cell morphological alterations were induced by all feed supplements, except CB7b, indicated by increased cell diameters throughout all culture phases (Table 2).

**Figure 4:**
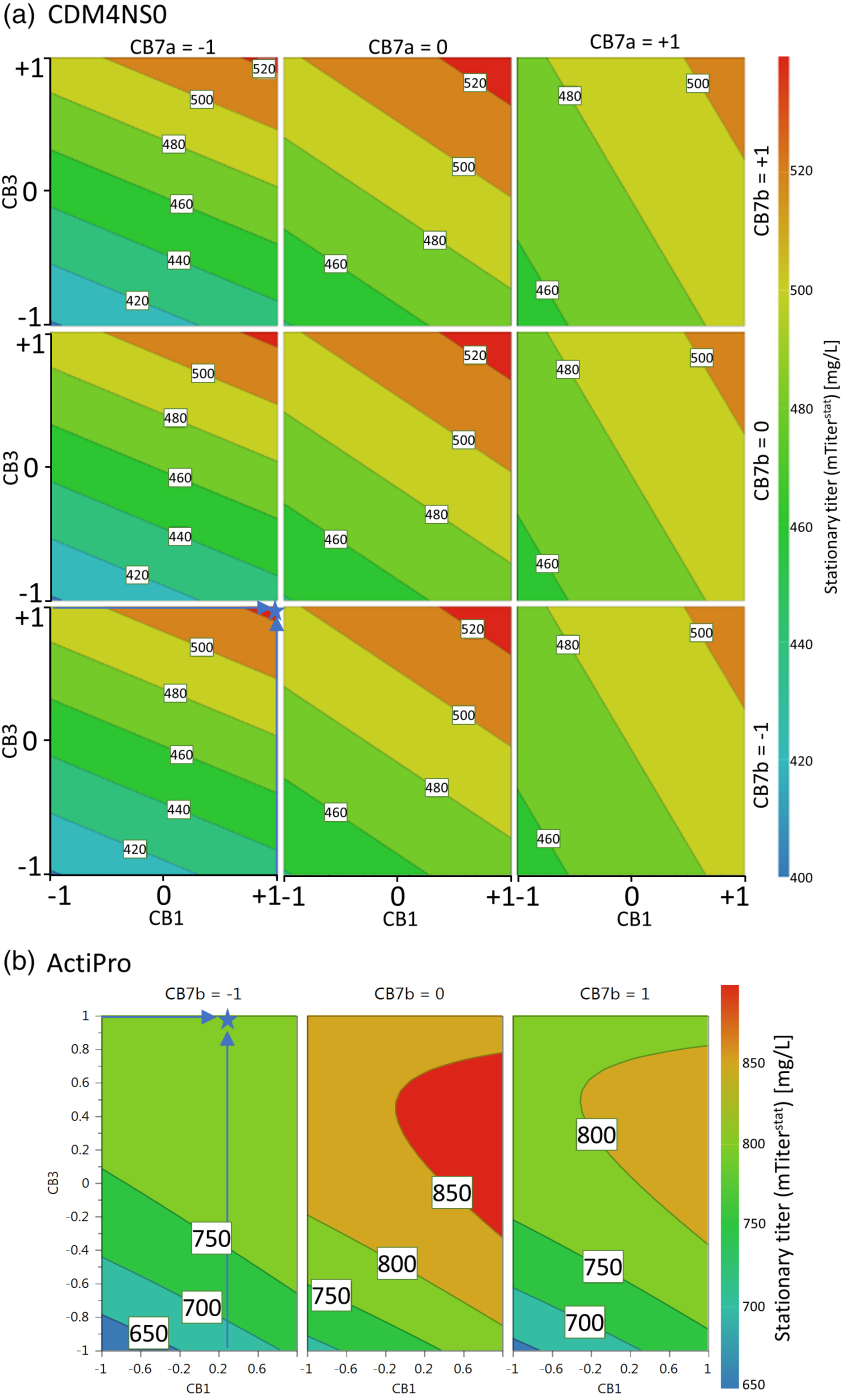
Contour plot of regression models for the stationary mean titer mTiter^stat^ of semicontinuous perfusion cultures in CDM4NS0 (a) and ActiPro (b). The final two feed combinations for the CDM4NS0 or ActiPro perfusion media are indicated by blue stars. Note that CB7b was suggested for the ActiPro model, but was omitted in the final media formulation because of solubility issues and to reduce the complexity but at a reasonably high‐predicted stationary titer

Established regression models for stationary titer values were finally leveraged to define the best supplement factor levels that were then transformed into the respective spike concentrations (Figure 4). Optimal combination of CB1 and CB3 at Level +1 was calculated for CDM4NS0 keeping CB7a and CB7b at −1 resulting in a predicted titer of 520 mg/L (Figure 4a). CB1 and CB3 at Level +1 translates into a final spike concentrations of 11.06% CB1 and 19.9% CB3 for CDM4NS0. For ActiPro optimal factor levels were determined for CB1 at +1, CB3 at +0.26, and CB7b at +0.1. However, since the model suggested only minor CB7b amounts of 0.26%, this feed supplement was omitted with keeping reasonable high predicted titer values above 750 mg/L (Figure 4b). Consequently, the final ActiPro perfusion medium was spiked with 13.94% CB1 and 15.8% CB3.

### 3.3 Verification of performance by semicontinuous small‐scale models and bioreactor perfusion experiments

**Figure 5:**
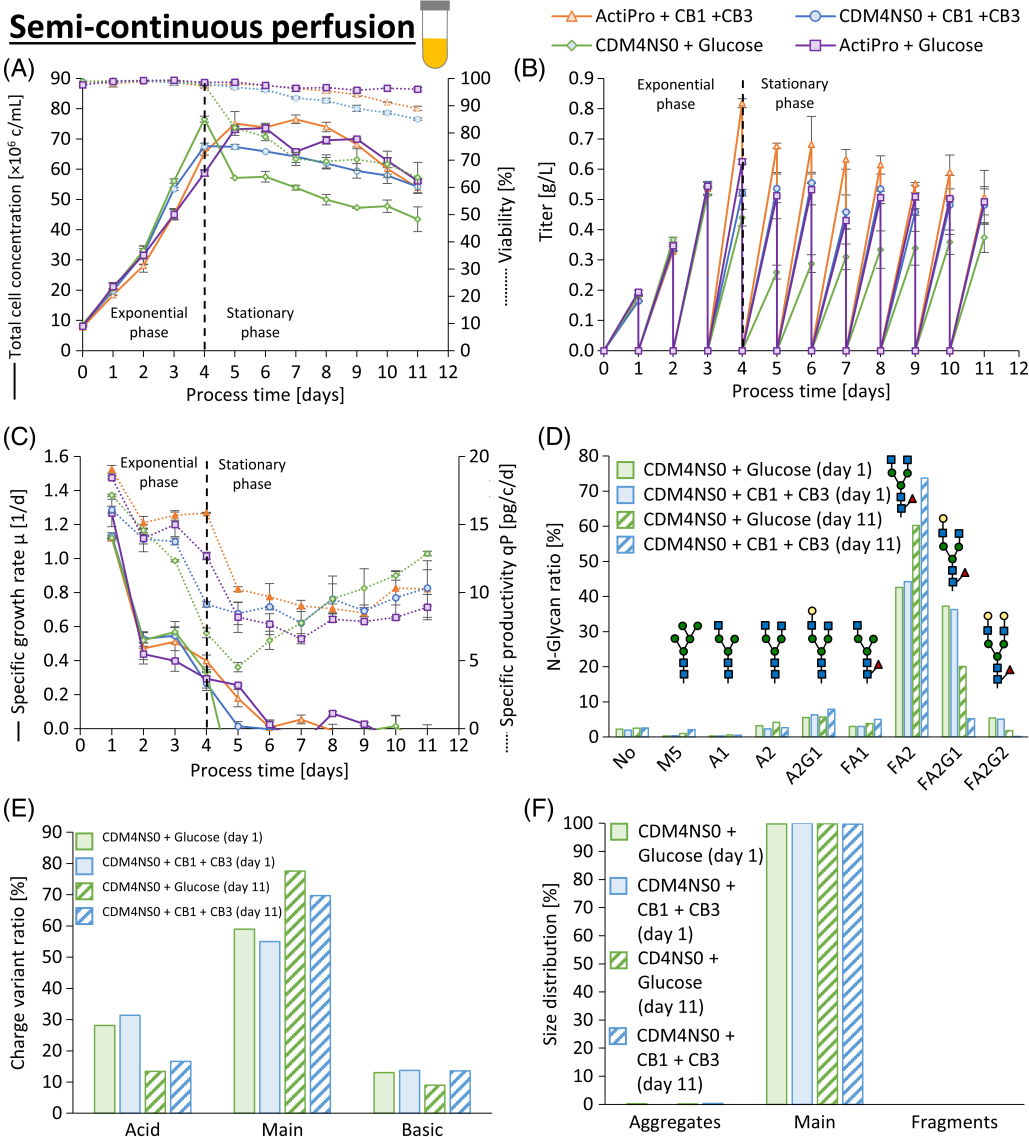
Perfusion media performance verification in semicontinuous small‐scale models. The CB1‐ and CB3 spiked ActiPro (orange triangles) and CDM4NS0 (blue circles) perfusion media were compared to the respective basal media that was spiked only with glucose to the level of the respective perfusion medium (purple squares for ActiPro and green diamonds for CDM4NS0). Total cell concentration (a) and antibody titer (b) was recorded to investigate differences in specific growth and productivity (c) in the exponential‐ and stationary phase. Highly similar product quality profiles were confirmed by N‐glycosylation (d), charge‐ (e) and size distribution (f) for the basal CDM4NS0 and CB1‐ and CB3‐spiked perfusion medium. Each condition was run in duplicate

**Figure 6:**
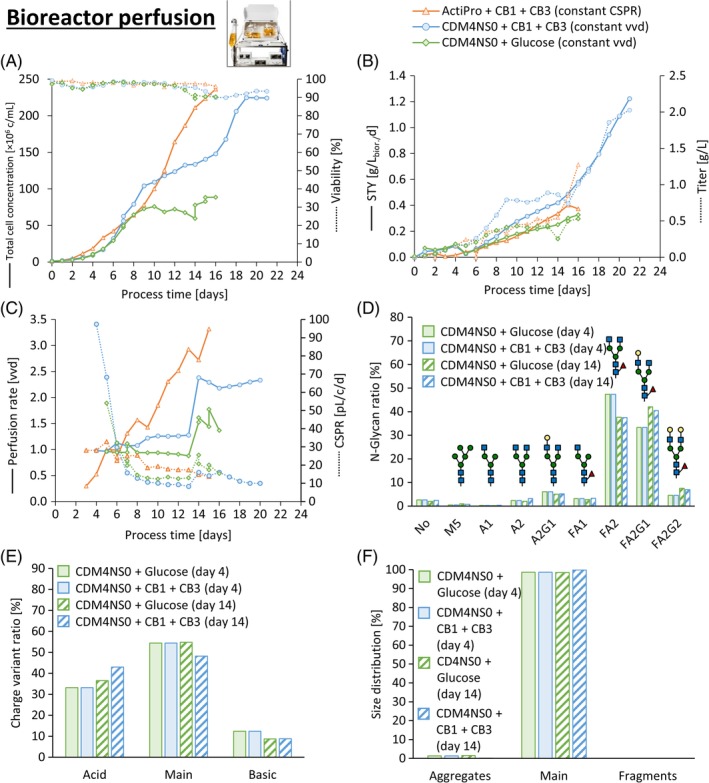
Bioreactor perfusion verification with the final perfusion media. Glucose‐spiked CDM4NS0 control (green diamonds) was compared to the CB1‐ and CB3 spiked CDM4NS0 perfusion medium (blue circles) at constant volumetric perfusion rates of one to two vvd. The performance of CB1‐ and CB3 spiked ActiPro perfusion medium (orange triangles) was tested at constantly low CSPR of 15 to 30 pL/c/d. Exceptionally high total cell numbers and viabilities (a) were reached, accumulating in high titer and space–time‐yields (STYs) (b) at very low CSPR (c). Highly similar product quality profiles as assessed by N‐glycosylation (d), charge‐ (e) and size (f) distribution were obtained for the two CDM4NS0 runs. CSPR, cell‐specific perfusion rates

The optimized CDM4NS0 and ActiPro perfusion media were finally applied to 10 ml semicontinuous perfusion models (Figure 5) and two 500 ml bioreactor verification runs using two different strategies (Figure 6) to identify possible media limitation and to demonstrate applicability to bioreactor perfusion cultures.

Mean stationary VCD^stat^ of semicontinuous perfusion cultures was increased up to 53% by spiking CDM4NS0 with CB1 and CB3 to maintain higher viabilities after day four compared to the nonspiked control (Figure 5a). As a combined effect of higher VCD^stat^ and higher qP^stat^ (Figure 5c), the mean daily harvested product titer was increased by 56% up to 0.5 g/L (Figure 5b) and was accurately predicted by the mTiter regression model (Figure 4a). Similarly, mean daily product titer was increased by 22% up to 0.6 g/L when spiking ActiPro with CB1 and CB3. The increased titer is a consequence of increased specific productivity (Figure 5c) rather than an increase in stationary VCD^stat^ (Figure 5a).

Usually the primary and first objective during media optimization is to increase VCD and harvest titer. Despite the two markers are often used for describing best cell performance, the focus of critical parameters is shifted toward increased product quality.^24^, ^25^, ^26^ Thereby, critical quality attributes have to be defined for each molecule of interest individually and quality characteristics are predominantly defined by the process operation mode/conditions and harvest time.^22^, ^27^, ^28^, ^29^, ^30^, ^31^, ^32^, ^33^

In semicontinuous small‐scale models, N‐glycosylation and charge profiles changed distinctly along different culture harvest times, but not by the addition of the CB1 and CB3 feed supplements (Figure 5d,e). The variation of product quality attributes was higher between different harvest times than between different media. The strong decrease of galactosylated N‐glycan species over cultivation time can be explained by increasing levels of ammonia in semicontinuous perfusion models (Supporting Information S11E). It is known that elevated ammonia levels inhibit activity of glyco‐attaching enzymes including galactosyl transferases.^34^, ^35^, ^36^ Final ammonia levels and specific ammonia production rates in the stationary phase were increased by feed supplementation (Supporting Information S11E and J), whereas specific glucose consumption and lactate production were consistent throughout all cultures at high viabilities (Supporting Information S11F and G).

The CB1‐ and CB3 spiked CDM4NS0 perfusion medium (Figure 6, blue circles) was run at constant volumetric perfusion rates in bioreactors at one vvd starting on day four until day 14 and was then increased to two vvd for additional 7 days (Figure 6c). Two steady‐state cell concentrations were reached at 130 × 10^6^ c/ml at one vvd and 220 × 10^6^ c/ml at two vvd (Figure 6a), translating into a minimum CSPR of 8–10 pL/c/d with titers of 0.9 and 1.9 g/L, respectively (Figure 6b,c). Oxygen transfer was sufficient to support such exceptionally high VCD values. We have estimated the cell‐specific oxygen consumption rate to 7.0 pmol/c/d based on the achieved VCDs and OTRs (results not shown). That value did not change much independently of the culture conditions. The STY of the entire process approached 1.2 g/L/d (Figure 6b). Spiking the basal CDM4NS0 with CB1 and CB3 increased the cell concentration and titer at one vvd by 96 and 105%, respectively, compared to a similar process with basal CDM4NS0 spiked only with glucose (Figure 6, green diamonds). No major changes in N‐glycan‐ or charge profiles were induced by CB1‐ or CB3 supplementation, but rather because of different sampling points in the early (Day 4 batch) phase or at later (Day 14) perfusion phase (Figure 6d,e). No reduction in galactosylated N‐glycan species was observed in bioreactor CDM4NS0 cultures over process time as a result of constantly lower ammonia levels below 30 mg/L (Supporting Information S13E) compared to the semicontinuous small‐scale models at 157 mg/L (Supporting Information S11E).

The ActiPro perfusion medium was used to test an alternative strategy based on fixed CSPR at 30 pL/c/d starting on Day 3 (Figure 6c, orange triangles). Using this strategy, the feed rate was increased every 24 hr according to the increase in VCD. CSPR setpoint values were subsequently decreased to 14 pL/c/d to optimize media consumption and to increase the measured titer. Very high cell numbers of up to 240 × 10^6^ c/ml at 96% viability were generated in a 16‐days process cumulating perfusion titers of 1.3 g/L and a final STY of 0.4 g/L/d (Figure 6b). Both bioreactor runs operated at very low CSPR values demonstrate the high performance of the two novel perfusion media developed from the two‐step DoE approach.

## 4 CONCLUSION

The main goal of improved perfusion media compositions is the reduction of cell‐specific media volume in combination with high viable cell concentrations, high cell‐specific productivities, and consistent product qualities. In this study, we investigated the benefit of a rapid and empirical DoE‐driven workflow to develop novel perfusion media by blending chemically defined basal media and established feed supplements. Using this empirical approach, a larger design space can be investigated when the exact media ingredients are unknown, compared to a more rational and targeted optimization approach.^37^ Rapid screening of a total of eight different cell supplement solutions in two different basal media was demonstrated. Semicontinuous perfusion by daily media exchange was performed in shaking tubes that resulted in high VCD and stable daily steady‐state titers. This generally applicable approach efficiently provides novel and high‐performing perfusion media within 1–2 months for any given cell line at minimal resources and personnel demands. The performed statistical analyses by Pearson correlation and regression analyses was highly suitable to abstract the multivariate data information and to detect meaningful correlations. We found that high batch titers correlated with high cumulative cell days rather than qP. In contrast, higher qP values were responsible for higher titers in the semicontinuous perfusion models and not cultures with highest cumulative cell days. Regression analysis was used to quantify the impact of individual feed supplements on cell growth, productivity, metabolism, and morphology in detail by fitting the observed responses with suitable regression models. The established models were finally leveraged to define two novel high‐performing perfusion media suitable for achieving more than 200 × 10^6^ c/ml within 2 weeks at viabilities above 90% in perfusion bioreactors. High cell densities were supported at minimal CSPR as low as 10–30 pL/c/d and no changes of product quality were induced by the included feed supplements. The high‐performing perfusion media, developed from this empirical design approach, can subsequently be used as starting point for further rational and additional targeted optimization.^37^

## Supporting information

Supporting Information
