# Supplementary material for: Rapid development of clone‐specific, high‐performing perfusion media from established feed supplements
Source: Biotechnol Prog. 2019 Nov 11;36(2):e2933. doi: 10.1002/btpr.2933 (PMC7187557; doi:10.1002/btpr.2933)

Supporting Information

Supplementary Information 1 Graphical representation of spiked batch culture data from the screening design (DoE1). *CDM4NS0 (A., left panels) or ActiPro basal media (B., right panels) were spiked with different combinations of CB 1, 2, 3, 4, 5, 6, 7a and 7b according to the DoE1 matrix (Figure 1A).*

A. CDM4NS0 | B. ActiPro

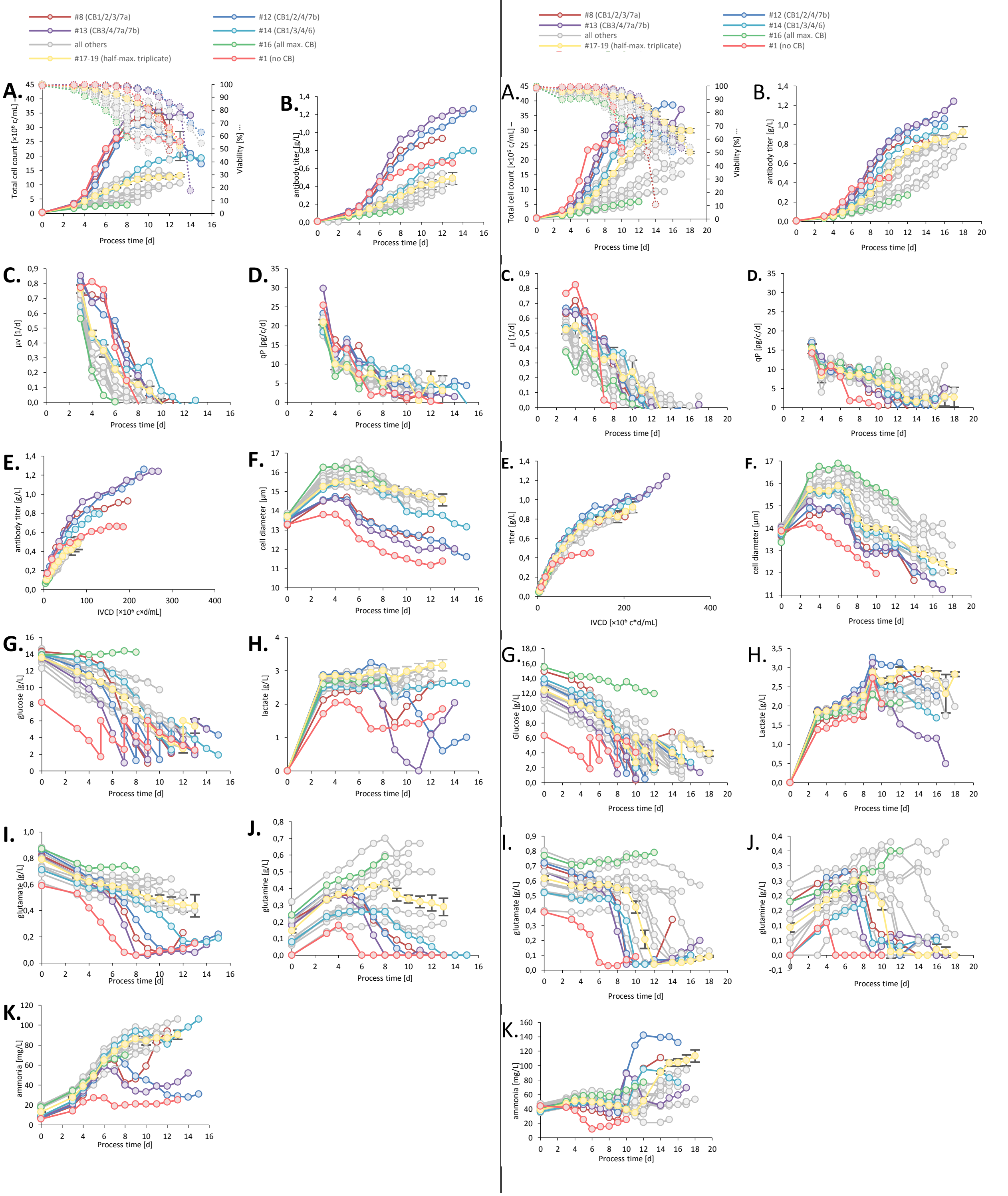

Supplementary Information 2 Summary table of spiked batch experiments (DoE1) in CDM4NS0 (A.) or ActiPro (B.).

A. CDM4NS0:

DoE run	CB1	CB2	CB3	CB4	CB5	CB6	CB7a	CB7b	max. process time [days]	max. exponential growth time (µ>0.2) [days]	pCC [mio. c/mL]	max. cell diameter [µm]	mean. cell diameter [µm]	min. cell diameter [µm]	pTiter [g/L]	max./start Gluc [mg/L]	first Gluc below 3 g/L [days]	max. Lac [mg/L]	max./start Glu [mg/L]	max. Gln [mg/L]	max. NH4+ [mg/L]	start Osm. [mOsm/kg]	max. process qP [pg/c/d]	mean process qP [pg/c/d]	min. process qP [pg/c/d]	max. qP in expon. [pg/c/d]	mean qP in expon. [pg/c/d]	min. qP in expon. [pg/c/d]	IVCD [$\times 10^9$ c×d]	max. µv in expon. [1/d]	mean µv in expon. [1/d]	min. µv in expon. [1/d]	max. qGluc cons. in expon. [pg/c/d]	max. qLac prod. in expon. [pg/c/d]	max. qGlu cons. in expon. [pg/c/d]	max. qGln prod. in expon. [pg/c/d]	max. qNH4+ prod. in expon. [pg/c/d]	mean qGluc cons. in expon. [pg/c/d]	mean qLac prod. in expon. [pg/c/d]	mean qGlu cons. in expon. [pg/c/d]	mean qGln prod. in expon. [pg/c/d]	mean qNH4+ prod. in expon. [pg/c/d]	min. qGluc cons. in expon. [pg/c/d]	min. qLac prod. in expon. [pg/c/d]	min. qGlu cons. in expon. [pg/c/d]	min. qGln prod. in expon. [pg/c/d]	min. qNH4+ prod. in expon. [pg/c/d]
#1	-1	-1	-1	-1	-1	-1	-1	-1	13	6	26.74	14	12	11	664	8220	5	2060	590	180	27	295	25.4	6.4	-0.4	25.4	15.2	7.4	4.17	0.81	0.68	0.37	848.2	460.5	22.4	35.0	2.2	360.7	129.7	13.8	8.2	1.1	118.5	-10.6	6.2	-6.5	0.0
#2	+1	-1	-1	-1	-1	+1	+1	+1	12	6	13.20	15	15	14	464	14600	12	3090	740	290	94	363	19.5	7.0	1.7	19.5	11.4	6.7	1.78	0.75	0.43	0.22	750.0	667.2	24.0	37.4	4.3	300.7	171.1	10.8	12.4	3.0	116.7	-7.9	1.3	1.3	2.3
#3	-1	+1	-1	-1	+1	-1	+1	+1	10	4	6.79	16	15	14	217	12950	20*	2960	840	610	76	360	18.4	6.7	-0.5	18.4	14.2	10.1	0.80	0.76	0.50	0.24	972.0	779.4	40.3	48.9	4.0	585.2	389.7	27.6	29.0	3.5	198.3	0.0	15.0	9.0	3.0
#4	+1	+1	-1	-1	+1	+1	-1	-1	9	5	4.69	16	16	14	171	14390	20*	2590	730	490	80	369	19.7	8.2	3.8	19.7	14.8	9.9	0.52	0.71	0.46	0.21	655.1	896.6	40.3	54.9	5.5	382.7	453.5	20.1	30.9	4.5	110.3	10.3	0.0	6.9	3.4
#5	-1	-1	+1	-1	+1	+1	+1	-1	8	3	3.95	16	16	14	157	13810	20*	2560	680	400	79	344	19.3	9.3	4.6	19.3	19.3	19.3	0.43	0.64	0.64	0.64	773.8	837.1	35.2	45.7	5.6	773.8	837.1	35.2	45.7	5.6	773.8	837.1	35.2	45.7	5.6
#6	+1	-1	+1	-1	+1	-1	-1	+1	13	6	10.58	16	15	13	444	14210	20*	2710	610	200	106	344	17.4	8.3	3.9	17.4	12.1	9.9	1.23	0.69	0.37	0.23	578.7	832.6	30.5	47.4	5.4	261.1	214.4	9.4	14.4	4.4	109.9	-6.7	0.0	0.0	3.7
#7	-1	+1	+1	-1	-1	+1	-1	+1	12	6	14.92	15	15	14	541	13310	9	3160	730	280	86	342	19.9	7.4	2.4	19.9	11.7	7.7	1.97	0.81	0.46	0.22	793.9	716.1	32.2	40.2	4.0	337.0	178.1	12.4	11.9	2.9	162.8	-12.2	1.6	0.0	2.1
#8	+1	+1	+1	-1	-1	-1	+1	-1	12	8	36.54	15	14	13	930	14300	9	2610	820	360	94	385	21.7	8.6	0.7	21.7	13.4	6.5	4.27	0.72	0.53	0.20	188.4	664.3	40.7	37.6	3.8	134.9	118.9	13.3	5.9	1.5	103.2	-23.4	5.1	-5.7	-0.8
#9	-1	-1	-1	+1	+1	+1	-1	+1	9	4	3.95	17	16	14	164	12280	20*	3100	710	440	91	342	17.4	8.7	3.5	17.4	14.0	10.6	0.47	0.60	0.42	0.23	1195.5	1049.4	37.5	60.0	6.0	729.8	547.8	28.0	39.2	5.8	264.0	46.3	18.5	18.5	5.6
#10	+1	-1	-1	+1	+1	-1	+1	-1	10	5	6.21	16	15	14	211	14140	20*	2790	800	550	78	387	18.7	7.4	1.0	18.7	11.9	8.1	0.72	0.73	0.39	0.22	353.5	917.1	43.4	50.0	5.0	181.7	310.6	19.1	22.2	3.5	75.3	0.0	2.9	5.8	2.3
#11	-1	+1	-1	+1	-1	+1	+1	-1	11	5	8.61	16	15	14	295	13580	20*	2910	880	700	76	383	22.2	8.1	2.2	22.2	13.6	7.7	0.92	0.75	0.46	0.28	813.6	970.1	52.2	55.6	5.2	376.7	326.3	23.0	25.6	3.5	101.8	3.6	2.6	10.4	1.8
#12	+1	+1	-1	+1	-1	-1	-1	+1	15	7	31.01	15	13	12	1261	13900	8	3240	860	410	73	389	23.2	8.0	1.6	23.2	14.4	10.3	4.75	0.82	0.59	0.32	498.7	941.7	49.2	52.5	4.3	247.7	193.8	15.6	9.7	2.2	155.0	-2.7	2.7	-6.1	0.5
#13	-1	-1	+1	+1	-1	-1	+1	+1	14	7	37.31	15	13	12	1241	13480	7	2670	810	360	59	360	29.8	8.0	1.4	29.8	15.5	9.8	5.65	0.85	0.59	0.26	692.7	711.5	37.7	43.1	3.5	290.8	142.5	13.5	6.9	1.6	143.3	-5.8	5.1	-6.1	-0.2
#14	+1	-1	+1	+1	-1	+1	-1	-1	15	9	20.40	16	14	13	797	13890	11	2640	710	260	106	365	18.4	7.8	-0.3	18.4	10.9	7.5	2.42	0.65	0.35	0.22	208.0	852.1	34.2	51.3	5.1	158.6	119.8	9.4	7.6	2.7	81.2	-25.7	0.0	-7.5	0.6
#15	-1	+1	+1	+1	+1	-1	-1	-1	12	5	9.72	16	15	14	346	13610	20*	2870	800	540	91	364	22.0	7.8	2.6	22.0	14.1	8.9	1.11	0.73	0.47	0.34	715.9	937.8	46.5	60.8	5.0	330.5	314.0	20.9	25.7	3.6	99.4	-10.4	5.2	5.2	2.1
#16	+1	+1	+1	+1	+1	+1	+1	+1	8	4	2.89	16	16	14	119	14370	20*	2720	870	590	70	410	20.3	8.8	3.5	20.3	13.5	6.8	0.33	0.56	0.39	0.21	60.2	1093.4	46.3	75.7	6.7	-9.9	571.3	34.1	46.1	5.6	-79.9	49.3	21.9	16.4	4.4
#17	0	0	0	0	0	0	0	0	13	10	12.84	16	15	14	486	13530	12	3010	780	440	86	363	20.5	7.5	1.1	20.5	8.9	4.7	1.67	0.75	0.26	0.01	561.6	906.9	42.0	54.9	4.8	196.4	114.2	8.3	8.3	1.9	99.9	-67.7	-1.0	-13.1	-0.5
#18	0	0	0	0	0	0	0	0	13	9	12.97	15	15	14	449	13560	12	3260	790	430	95	363	21.2	7.0	-1.6	21.2	9.1	4.5	1.68	0.79	0.30	0.04	524.1	925.3	42.1	64.7	4.9	202.7	133.2	9.8	11.0	2.2	106.2	-11.2	0.0	-4.1	0.6
#19	0	0	0	0	0	0	0	0	13	10	13.64	16	15	14	528	13580	12	3270	800	420	90	362	21.2	7.6	1.4	21.2	9.2	6.0	1.79	0.74	0.28	0.01	611.9	981.8	51.9	65.7	4.8	201.8	126.2	10.1	9.3	1.9	90.3	-12.5	1.3	-6.5	-0.1
min.									8	3	2.89	14	12	11	119	8220	5	2060	590	180	27	295	17.4	6.4	-1.6	17.4	8.9	4.5	0.33	0.56	0.26	0.01	60.2	460.5	22.4	35.0	2.2	-9.9	114.2	8.3	5.9	1.1	-79.9	-67.7	-1.0	-13.1	-0.8
max.									15	10	37.31	17	16	14	1261	14600	12	3270	880	700	106	410	29.8	9.3	4.6	29.8	19.3	19.3	5.65	0.85	0.68	0.64	1195.5	1093.4	52.2	75.7	6.7	773.8	837.1	35.2	46.1	5.8	773.8	837.1	35.2	45.7	5.6
mean									12	6	14.58	16	15	13	499	13458	n.a.*	2854	766	418	82	363	20.9	7.8	1.7	20.9	13.0	8.6	1.93	0.73	0.45	0.24	620.8	849.5	39.4	51.7	4.7	318.1	283.8	17.6	19.5	3.2	148.9	39.5	6.5	3.4	1.9
Stdv.									2.162	2.104	10.87	1	1	1	346	1383	2.497	308	81	141	18	24	3.0	0.8	1.7	3.0	2.6	3.2	1.61	0.08	0.12	0.14	279.0	153.6	8.3	10.8	1.0	196.4	199.0	8.5	13.4	1.4	164.7	194.7	9.5	13.4	2.0

B. ActiPro:

DoE run	CB1	CB2	CB3	CB4	CB5	CB6	CB7a	CB7b	max. process time [days]	max. exponential growth time (µ>0.2) [days]	pCC [mio. c/mL]	max. cell diameter [µm]	mean. cell diameter [µm]	min. cell diameter [µm]	pTiter [g/L]	max./start Gluc [mg/L]	first Gluc below 3 g/L [days]	max. Lac [mg/L]	max./start Glu [mg/L]	max. Gln [mg/L]	max. NH4+ [mg/L]	start Osm. [mOsm/kg]	max. process qP [pg/c/d]	mean process qP [pg/c/d]	min. process qP [pg/c/d]	max. qP in expon. [pg/c/d]	mean qP in expon. [pg/c/d]	min. qP in expon. [pg/c/d]	IVCD [$\times 10^9$ c×d]	max. µv in expon. [1/d]	mean µv in expon. [1/d]	min. µv in expon. [1/d]	max. qGluc cons. in expon. [pg/c/d]	max. qLac prod. in expon. [pg/c/d]	max. qGlu cons. in expon. [pg/c/d]	max. qGln prod. in expon. [pg/c/d]	max. qNH4+ prod. in expon. [pg/c/d]	mean qGluc cons. in expon. [pg/c/d]	mean qLac prod. in expon. [pg/c/d]	mean qGlu cons. in expon. [pg/c/d]	mean qGln prod. in expon. [pg/c/d]	mean qNH4+ prod. in expon. [pg/c/d]	min. qGluc cons. in expon. [pg/c/d]	min. qLac prod. in expon. [pg/c/d]	min. qGlu cons. in expon. [pg/c/d]	min. qGln prod. in expon. [pg/c/d]	min. qNH4+ prod. in expon. [pg/c/d]
#1	-1	-1	-1	-1	-1	-1	-1	-1	10	6	26.6	14.2	13.2	12.0	452	6300	5	2730	390	110	44	306	14.3	6.0	0.5	14.3	10.6	8.1	2.8	0.83	0.71	0.61	562.6	398	14.4	26.0	-0.6	274.0	107.3	10.3	4.7	-0.9	168.9	8.7	5.3	-11.6	-1.4
#2	+1	-1	-1	-1	-1	+1	+1	+1	16	12	24.3	15.7	14.6	12.7	762	13100	12	2710	610	250	88	374	14.4	7.0	0.5	14.4	9.2	6.3	2.3	0.56	0.35	0.22	805.7	819	17.9	62.7	3.6	233.1	112.2	4.0	9.5	0.8	30.1	-23.6	-6.9	-8.4	-0.4
#3	-1	+1	-1	-1	+1	-1	+1	+1	17	7	15.1	16.1	15.0	13.7	657	10900	15	2620	740	380	57	373	14.8	7.8	3.3	14.8	9.4	6.6	1.5	0.62	0.36	0.21	812.3	808	21.4	12.8	2.6	313.5	207.8	7.4	4.4	1.0	116.0	-4.8	-8.9	0.0	-0.6
#4	+1	+1	-1	-1	+1	+1	-1	-1	17	8	26.3	16.0	14.4	12.8	860	13470	11	2740	580	230	94	375	13.5	6.7	0.9	13.5	9.9	8.2	2.7	0.54	0.41	0.34	623.3	726	17.1	34.2	3.0	260.2	138.2	2.3	9.8	0.8	112.6	8.3	-7.2	1.7	-0.5
#5	-1	-1	+1	-1	+1	+1	+1	-1	15	7	9.3	16.7	15.6	13.8	480	12140	15	2280	570	310	64	351	14.4	10.1	6.4	14.4	12.4	9.8	0.9	0.39	0.32	0.23	796.8	833	15.5	46.6	4.7	335.7	186.0	3.7	17.8	1.8	164.0	6.2	-5.4	4.0	-0.9
#6	+1	-1	+1	-1	+1	-1	-1	+1	15	9	25.4	15.7	14.1	12.2	781	13190	10	2740	410	80	77	353	13.3	6.5	1.6	13.3	10.2	8.2	3.2	0.60	0.43	0.25	592.6	694	7.1	21.3	2.7	239.3	114.2	2.1	3.4	0.6	101.7	7.5	-3.3	-2.2	-0.7
#7	-1	+1	+1	-1	-1	+1	-1	+1	17	9	31.0	15.8	13.9	12.0	889	11310	9	2960	580	210	79	352	13.8	5.9	1.5	13.8	9.6	7.4	3.7	0.60	0.42	0.28	665.6	700	15.2	38.0	2.7	231.7	116.0	4.6	7.0	0.6	89.7	4.3	-2.5	-5.3	-0.5
#8	+1	+1	+1	-1	-1	-1	+1	-1	14	8	34.3	14.9	13.7	11.7	858	14940	10	2840	700	280	111	400	16.4	6.3	-2.2	16.4	11.1	8.2	3.9	0.72	0.52	0.29	423.3	605	22.7	22.7	1.5	210.2	108.1	6.7	4.5	0.1	130.5	-16.6	0.0	-2.4	-0.4
#9	-1	-1	-1	+1	+1	+1	-1	+1	14	7	9.4	16.4	15.6	14.0	365	9890	11	2750	560	290	65	354	14.2	7.8	4.0	14.2	9.8	4.0	1.0	0.50	0.33	0.23	843.7	886	18.6	42.0	3.7	330.4	196.1	1.9	17.1	1.7	140.7	5.2	-6.7	4.4	-0.3
#10	+1	-1	-1	+1	+1	-1	+1	-1	18	8	19.7	16.3	15.0	13.2	775	13890	14	2560	660	360	57	400	15.3	7.6	1.0	15.3	9.9	6.3	1.8	0.52	0.34	0.21	781.6	894	25.5	30.7	3.1	273.3	162.5	4.6	11.1	0.7	30.4	4.9	-8.7	3.2	-0.7
#11	-1	+1	-1	+1	-1	+1	+1	-1	11	5	4.1	16.6	16.0	14.1	180	11950	20	2300	800	380	60	398	17.5	9.7	6.6	17.5	12.0	7.5	0.4	0.55	0.38	0.27	943.9	1162	46.2	33.0	5.9	449.4	398.3	16.1	10.3	3.8	180.4	8.3	-6.2	-8.3	0.6
#12	+1	+1	-1	+1	-1	-1	-1	+1	16	8	38.9	14.9	13.5	11.5	1059	13330	9	3260	720	280	142	413	17.0	6.4	-1.1	17.0	10.9	8.2	4.5	0.67	0.53	0.36	782.6	791	25.1	19.2	2.1	264.9	144.2	8.6	4.7	0.3	104.2	0.0	-4.2	-5.2	-0.2
#13	-1	-1	+1	+1	-1	-1	+1	+1	17	8	37.1	15.2	13.3	11.2	1243	11810	8	3120	660	250	89	377	14.7	6.2	0.8	14.7	11.0	7.7	5.3	0.65	0.53	0.31	696.9	630	23.6	20.2	1.0	246.7	113.3	7.4	3.7	0.1	126.8	5.0	1.0	-5.4	-0.5
#14	+1	-1	+1	+1	-1	+1	-1	-1	16	9	30.3	15.7	14.1	12.0	980	13900	10	2810	520	170	95	377	14.8	7.1	1.0	14.8	11.3	9.6	3.5	0.54	0.45	0.34	668.6	762	13.4	35.7	3.1	233.2	127.3	4.2	7.8	0.9	63.2	10.0	-6.0	-7.6	-0.2
#15	-1	+1	+1	+1	+1	-1	-1	-1	15	9	20.9	16.2	14.6	12.3	763	12240	11	2560	660	290	56	376	15.7	8.1	3.5	15.7	10.6	7.1	2.1	0.55	0.38	0.21	773.1	826	23.9	34.5	3.8	258.5	136.1	4.9	8.8	0.6	91.8	9.7	-4.5	-2.2	-0.7
#16	+1	+1	+1	+1	+1	+1	+1	+1	12	6	5.9	16.9	16.0	13.4	271	15520	20	2300	790	350	77	422	15.8	10.2	6.9	15.8	12.2	10.4	0.5	0.40	0.32	0.24	599.9	922	32.7	17.4	5.5	237.9	254.0	6.8	7.5	2.9	37.7	9.0	-18.9	-12.6	0.0
#17	0	0	0	0	0	0	0	0	18	15	30.0	15.9	14.1	12.1	962	12480	10	2910	600	270	122	372	14.6	6.3	-0.1	14.6	8.2	3.0	3.4	0.54	0.29	0.00	704.3	753	12.3	20.5	3.3	183.7	79.7	3.9	3.3	0.5	55.1	-13.9	-4.1	-7.7	-0.4
#18	0	0	0	0	0	0	0	0	18	12	30.5	16.0	14.1	12.0	906	12360	10	3030	630	250	113	371	15.4	6.3	-0.4	15.4	9.0	5.2	3.3	0.54	0.32	0.11	758.3	798	26.1	34.9	3.5	195.7	94.2	5.6	4.6	0.5	76.5	-17.2	-6.3	-9.3	-0.6
#19	0	0	0	0	0	0	0	0	18	12	30.2	15.8	14.1	12.0	904	12460	10	2980	620	250	105	368	16.1	6.0	-1.2	16.1	8.6	3.9	3.4	0.64	0.32	0.12	717.0	818	22.0	30.8	3.5	200.0	95.0	5.5	4.0	0.5	76.5	-13.4	-1.9	-7.9	-0.4
min.									10	5	4.1	14.2	13.2	11.2	180	6300	5	2280	390	80	44	306	13.3	5.9	-2.2	13.3	8.2	3.0	0.4	0.4	0.3	0.0	423.3	398	7.145	12.83	-0.58	183.7	79.7	1.867	3.252	-0.89	30.1	-23.6	-18.9	-12.6	-1.38
max.									18	15	38.9	16.9	16.0	14.1	1243	15520	20	3260	800	380	142	422	17.5	10.2	6.9	17.5	12.4	10.4	5.3	0.8	0.7	0.6	943.9	1162	46.21	62.67	5.941	449.4	398.3	16.09	17.84	3.847	180.4	10.04	5.289	4.434	0.622
mean									15	9	23.6	15.8	14.5	12.6	744	12378	12	2747	621	263	84	374	15.1	7.3	1.8	15.1	10.3	7.1	2.6	0.6	0.4	0.3	713	780	21	31	3	262	152	6	8	1	100	0	-5	-4	0
Stdv.									2	2	10.5	0.7	0.9	0.9	279	1991	4	271	109	81	26	26	1.1	1.4	2.7	1.1	1.2	2.0	1.4	0.1	0.1	0.1	120	152	9	12	1	62	75	3	4	1	46	11	5	5	0

Supplementary Information 3 Pearson correlation matrix of spiked batch culture data (DoE1) in CDM4NS0 (A.) or ActiPro (B.).

A. CDM4NS0

	CB1	CB2	CB3	CB4	CB5	CB6	CB7a	CB7b	max time	max time exp	max pCC	max diam	mean diam	min_diam	max circ	mean circ	min circ	pTiter	max Gluc	first Gluc below 3000	max Lac	max Glu	max Gln	max NH4+	max. osmolality	max. qP	mean qP	min qP	max exp qP	mean exp qP	min exp qP	Max. IVCD	max exp µv	mean exp µv	min exp µv	max exp qGluc	max exp qLac	max exp qGlu	max exp qGln	max exp qNH4+	mean exp qGluc	mean exp qLac	mean exp qGlu	mean exp qGln	mean exp qNH4+	min exp qGluc	min exp qLac	min exp qGlu	min exp qGln	min exp qNH4+
CB1		0.0	0.0	0.0	0.0	0.0	0.0	0.0	0.1	0.3	0.1	0.1	0.1	0.0	0.3	0.0	-0.2	0.1	0.5	0.0	0.0	0.1	-0.2	0.4	0.5	-0.3	0.1	0.0	-0.3	-0.4	-0.3	0.0	-0.2	-0.3	-0.3	-0.7	0.2	0.0	0.1	0.3	-0.6	-0.2	-0.3	-0.2	0.0	-0.4	-0.3	-0.3	-0.3	-0.1
CB2	0.0		0.0	0.0	0.0	0.0	0.0	0.0	-0.1	-0.1	0.0	0.0	0.1	0.2	0.1	0.1	-0.2	0.0	0.2	0.1	0.3	0.6	0.5	0.0	0.5	0.0	0.1	0.0	0.0	0.0	-0.2	-0.1	0.1	0.0	-0.2	-0.1	0.3	0.6	0.3	0.1	-0.2	0.0	0.2	0.1	0.0	-0.3	-0.2	-0.1	-0.1	-0.1
CB3	0.0	0.0		0.0	0.0	0.0	0.0	0.0	0.1	0.2	0.2	0.0	0.0	0.1	-0.2	-0.1	0.1	0.2	0.3	-0.1	-0.2	-0.1	-0.3	0.3	0.1	0.1	0.4	0.2	0.1	0.0	0.1	0.1	-0.2	-0.1	0.1	-0.4	0.0	0.0	0.0	0.2	-0.3	0.0	-0.1	-0.1	0.0	0.1	0.2	0.2	0.0	0.0
CB4	0.0	0.0	0.0		0.0	0.0	0.0	0.0	0.1	0.1	0.0	0.1	0.0	0.0	0.2	0.3	0.3	0.1	0.1	0.1	0.2	0.5	0.4	0.0	0.5	0.2	0.2	0.0	0.2	-0.1	-0.1	0.0	-0.2	-0.2	-0.1	-0.2	0.6	0.6	0.6	0.4	-0.2	0.0	0.1	0.1	0.1	-0.3	-0.2	0.0	-0.1	-0.1
CB5	0.0	0.0	0.0	0.0		0.0	0.0	0.0	-0.7	-0.5	-0.8	0.8	0.7	0.6	-0.5	-0.7	-0.5	-0.7	0.2	0.8	0.0	-0.1	0.4	0.2	0.1	-0.5	0.3	0.5	-0.5	0.2	0.4	-0.7	-0.6	-0.2	0.1	0.1	0.5	0.2	0.5	0.6	0.3	0.7	0.6	0.7	0.7	0.2	0.3	0.5	0.6	0.7
CB6	0.0	0.0	0.0	0.0	0.0		0.0	0.0	-0.4	-0.2	-0.5	0.5	0.5	0.5	-0.4	-0.5	-0.3	-0.4	0.2	0.2	0.2	-0.1	0.1	0.3	0.1	-0.4	0.3	0.4	-0.4	0.0	0.1	-0.5	-0.5	-0.3	0.0	0.1	0.3	-0.1	0.2	0.5	0.2	0.4	0.3	0.4	0.5	0.2	0.3	0.2	0.4	0.5
CB7a	0.0	0.0	0.0	0.0	0.0	0.0		0.0	-0.4	-0.2	0.0	0.1	0.1	0.2	0.1	-0.2	-0.5	-0.1	0.3	0.2	0.0	0.5	0.4	-0.1	0.4	0.1	0.1	-0.1	0.1	0.1	0.1	-0.1	0.0	0.1	0.1	-0.2	0.0	0.2	0.0	0.0	-0.1	0.2	0.3	0.2	0.0	0.1	0.3	0.3	0.3	0.0
CB7b	0.0	0.0	0.0	0.0	0.0	0.0	0.0		0.1	-0.1	0.0	0.1	0.1	0.0	-0.4	-0.2	0.0	0.2	0.1	-0.1	0.5	0.1	-0.1	0.1	0.0	0.0	0.0	0.1	0.0	-0.1	-0.1	0.1	0.1	-0.1	-0.2	0.2	0.1	-0.1	0.1	0.0	0.0	-0.1	0.0	0.0	0.1	-0.1	-0.2	0.1	-0.1	0.2
max time	0.1	-0.1	0.1	0.1	-0.7	-0.4	-0.4	0.1		0.8	0.7	-0.7	-0.7	-0.5	0.3	0.7	0.8	0.8	-0.1	-0.7	0.2	-0.1	-0.5	0.1	-0.1	0.4	-0.4	-0.5	0.4	-0.4	-0.4	0.7	0.6	-0.1	-0.3	-0.2	-0.3	-0.1	-0.3	-0.5	-0.4	-0.9	-0.8	-0.9	-0.8	-0.3	-0.5	-0.7	-0.8	-0.8
max time exp	0.3	-0.1	0.2	0.1	-0.5	-0.2	-0.2	-0.1	0.8		0.5	-0.4	-0.3	-0.2	0.2	0.5	0.6	0.5	0.1	-0.6	0.3	0.1	-0.3	0.3	0.0	0.2	-0.3	-0.6	0.2	-0.7	-0.6	0.5	0.4	-0.5	-0.7	-0.4	-0.1	0.1	0.0	-0.3	-0.6	-0.8	-0.8	-0.8	-0.8	-0.4	-0.5	-0.7	-0.8	-0.8
max pCC	0.1	0.0	0.2	0.0	-0.8	-0.5	0.0	0.0	0.7	0.5		-0.9	-0.9	-0.8	0.7	0.7	0.5	0.9	-0.2	-0.9	-0.2	0.0	-0.5	-0.3	-0.1	0.7	-0.1	-0.4	0.7	0.0	-0.2	1.0	0.6	0.4	0.0	-0.2	-0.6	-0.1	-0.5	-0.7	-0.3	-0.6	-0.5	-0.7	-0.8	-0.2	-0.3	-0.4	-0.6	-0.8
max diam	0.1	0.0	0.0	0.1	0.8	0.5	0.1	0.1	-0.7	-0.4	-0.9		1.0	0.8	-0.6	-0.8	-0.5	-0.8	0.5	0.9	0.3	0.1	0.5	0.5	0.3	-0.7	0.5	0.6	-0.7	0.0	0.3	-0.9	-0.7	-0.5	0.0	0.1	0.7	0.3	0.6	0.9	0.3	0.7	0.6	0.8	0.9	0.2	0.3	0.4	0.6	0.8
mean diam	0.1	0.1	0.0	0.0	0.7	0.5	0.1	0.1	-0.7	-0.3	-0.9	1.0		0.9	-0.7	-0.8	-0.6	-0.8	0.5	0.8	0.4	0.1	0.5	0.6	0.3	-0.8	0.3	0.5	-0.8	-0.1	0.2	-0.9	-0.6	-0.6	-0.1	0.1	0.7	0.2	0.5	0.8	0.2	0.6	0.4	0.7	0.8	0.2	0.3	0.3	0.6	0.7
min_diam	0.0	0.2	0.1	0.0	0.6	0.5	0.2	0.0	-0.5	-0.2	-0.8	0.8	0.9		-0.6	-0.7	-0.5	-0.8	0.5	0.7	0.4	0.2	0.5	0.6	0.3	-0.7	0.2	0.3	-0.7	-0.3	0.0	-0.9	-0.5	-0.6	-0.2	0.1	0.6	0.3	0.5	0.7	0.1	0.4	0.3	0.5	0.6	0.1	0.1	0.2	0.4	0.5
max circ	0.3	0.1	-0.2	0.2	-0.5	-0.4	0.1	-0.4	0.3	0.2	0.7	-0.6	-0.7	-0.6		0.7	0.2	0.5	-0.1	-0.4	-0.3	0.3	0.0	-0.3	0.3	0.4	-0.1	-0.4	0.4	0.1	-0.3	0.6	0.3	0.3	0.0	-0.4	-0.3	0.1	-0.3	-0.5	-0.4	-0.4	-0.2	-0.4	-0.5	-0.4	-0.4	-0.3	-0.4	-0.6
mean circ	0.0	0.1	-0.1	0.3	-0.7	-0.5	-0.2	-0.2	0.7	0.5	0.7	-0.8	-0.8	-0.7	0.7		0.7	0.7	-0.5	-0.6	-0.2	0.1	-0.2	-0.5	-0.1	0.7	-0.3	-0.5	0.7	-0.2	-0.5	0.7	0.5	0.2	-0.2	-0.2	-0.3	0.1	-0.2	-0.6	-0.5	-0.7	-0.5	-0.6	-0.7	-0.5	-0.5	-0.5	-0.7	-0.8
min circ	-0.2	-0.2	0.1	0.3	-0.5	-0.3	-0.5	0.0	0.8	0.6	0.5	-0.5	-0.6	-0.5	0.2	0.7		0.6	-0.4	-0.5	0.1	-0.2	-0.4	0.0	-0.4	0.4	-0.2	-0.4	0.4	-0.3	-0.3	0.5	0.3	-0.1	-0.2	0.1	-0.1	0.0	0.0	-0.3	-0.1	-0.6	-0.6	-0.6	-0.5	-0.2	-0.4	-0.4	-0.6	-0.5
pTiter	0.1	0.0	0.2	0.1	-0.7	-0.4	-0.1	0.2	0.8	0.5	0.9	-0.8	-0.8	-0.8	0.5	0.7	0.6		-0.1	-0.8	0.0	0.1	-0.5	-0.2	0.0	0.7	-0.1	-0.4	0.7	0.0	-0.2	1.0	0.6	0.3	-0.1	-0.2	-0.4	0.0	-0.4	-0.6	-0.3	-0.6	-0.6	-0.8	-0.7	-0.2	-0.3	-0.4	-0.6	-0.7
max Gluc	0.5	0.2	0.3	0.1	0.2	0.2	0.3	0.1	-0.1	0.1	-0.2	0.5	0.5	0.5	-0.1	-0.5	-0.4	-0.1		0.4	0.4	0.5	0.3	0.7	0.8	-0.3	0.5	0.3	-0.3	-0.2	0.0	-0.3	-0.3	-0.4	-0.2	-0.5	0.5	0.4	0.3	0.6	-0.3	0.1	0.0	0.1	0.3	-0.1	0.1	-0.1	0.1	0.2
first Gluc below 3000	0.0	0.1	-0.1	0.1	0.8	0.2	0.2	-0.1	-0.7	-0.6	-0.9	0.9	0.8	0.7	-0.4	-0.6	-0.5	-0.8	0.4		0.0	0.1	0.6	0.3	0.3	-0.6	0.4	0.5	-0.6	0.2	0.4	-0.9	-0.6	-0.2	0.2	0.1	0.6	0.3	0.5	0.8	0.3	0.7	0.7	0.8	0.8	0.2	0.3	0.4	0.7	0.8
max Lac	0.0	0.3	-0.2	0.2	0.0	0.2	0.0	0.5	0.2	0.3	-0.2	0.3	0.4	0.4	-0.3	-0.2	0.1	0.0	0.4	0.0		0.5	0.3	0.5	0.3	-0.2	-0.1	-0.1	-0.2	-0.5	-0.3	-0.2	0.1	-0.5	-0.5	0.1	0.5	0.4	0.4	0.3	0.0	-0.2	-0.2	-0.1	0.0	-0.1	-0.2	-0.2	-0.1	0.0
max Glu	0.1	0.6	-0.1	0.5	-0.1	-0.1	0.5	0.1	-0.1	0.1	0.0	0.1	0.1	0.2	0.3	0.1	-0.2	0.1	0.5	0.1	0.5		0.8	0.0	0.8	0.2	0.1	-0.2	0.2	-0.1	-0.3	0.0	0.1	-0.2	-0.3	-0.3	0.5	0.8	0.5	0.2	-0.3	0.0	0.2	0.1	-0.1	-0.3	-0.2	0.0	-0.1	-0.2
max Gln	-0.2	0.5	-0.3	0.4	0.4	0.1	0.4	-0.1	-0.5	-0.3	-0.5	0.5	0.5	0.5	0.0	-0.2	-0.4	-0.5	0.3	0.6	0.3	0.8		0.0	0.6	-0.1	0.2	0.1	-0.1	0.1	0.0	-0.5	-0.2	-0.2	-0.1	0.0	0.6	0.8	0.6	0.5	0.1	0.4	0.6	0.6	0.4	-0.1	0.0	0.2	0.4	0.2
max NH4+	0.4	0.0	0.3	0.0	0.2	0.3	-0.1	0.1	0.1	0.3	-0.3	0.5	0.6	0.6	-0.3	-0.5	0.0	-0.2	0.7	0.3	0.5	0.0	0.0		0.3	-0.6	0.3	0.1	-0.6	-0.5	-0.1	-0.4	-0.4	-0.6	-0.3	-0.2	0.4	0.1	0.2	0.5	-0.1	-0.1	-0.2	0.0	0.2	0.0	-0.1	-0.2	0.0	0.1
max. osmolality	0.5	0.5	0.1	0.5	0.1	0.1	0.4	0.0	-0.1	0.0	-0.1	0.3	0.3	0.3	0.3	-0.1	-0.4	0.0	0.8	0.3	0.3	0.8	0.6	0.3		-0.1	0.3	0.1	-0.1	-0.2	-0.2	-0.1	-0.2	-0.3	-0.2	-0.6	0.6	0.7	0.5	0.5	-0.5	0.1	0.2	0.2	0.2	-0.4	-0.2	0.0	0.0	0.0
max. qP	-0.3	0.0	0.1	0.2	-0.5	-0.4	0.1	0.0	0.4	0.2	0.7	-0.7	-0.8	-0.7	0.4	0.7	0.4	0.7	-0.3	-0.6	-0.2	0.2	-0.1	-0.6	-0.1		-0.2	-0.3	1.0	0.2	-0.1	0.8	0.6	0.5	0.1	0.0	-0.4	0.1	-0.2	-0.6	-0.2	-0.4	-0.2	-0.4	-0.6	-0.1	-0.1	-0.2	-0.4	-0.6
mean qP	0.1	0.1	0.4	0.2	0.3	0.3	0.1	0.0	-0.4	-0.3	-0.1	0.5	0.3	0.2	-0.1	-0.3	-0.2	-0.1	0.5	0.4	-0.1	0.1	0.2	0.3	0.3	-0.2		0.8	-0.2	0.4	0.5	-0.2	-0.6	0.1	0.4	-0.2	0.5	0.3	0.3	0.6	0.2	0.6	0.5	0.5	0.6	0.4	0.5	0.5	0.6	0.5
min qP	0.0	0.0	0.2	0.0	0.5	0.4	-0.1	0.1	-0.5	-0.6	-0.4	0.6	0.5	0.3	-0.4	-0.5	-0.4	-0.4	0.3	0.5	-0.1	-0.2	0.1	0.1	0.1	-0.3	0.8		-0.3	0.5	0.6	-0.4	-0.5	0.1	0.4	0.2	0.4	0.0	0.2	0.6	0.3	0.7	0.5	0.6	0.7	0.3	0.5	0.4	0.6	0.7
max exp qP	-0.3	0.0	0.1	0.2	-0.5	-0.4	0.1	0.0	0.4	0.2	0.7	-0.7	-0.8	-0.7	0.4	0.7	0.4	0.7	-0.3	-0.6	-0.2	0.2	-0.1	-0.6	-0.1	1.0	-0.2	-0.3		0.2	-0.1	0.8	0.6	0.5	0.1	0.0	-0.4	0.1	-0.2	-0.6	-0.2	-0.4	-0.2	-0.4	-0.6	-0.1	-0.1	-0.2	-0.4	-0.6
mean exp qP	-0.4	0.0	0.0	-0.1	0.2	0.0	0.1	-0.1	-0.4	-0.7	0.0	0.0	-0.1	-0.3	0.1	-0.2	-0.3	0.0	-0.2	0.2	-0.5	-0.1	0.1	-0.5	-0.2	0.2	0.4	0.5	0.2		0.8	0.1	-0.1	0.9	0.9	0.3	-0.2	-0.1	-0.3	0.0	0.6	0.7	0.7	0.5	0.4	0.6	0.6	0.7	0.7	0.5
min exp qP	-0.3	-0.2	0.1	-0.1	0.4	0.1	0.1	-0.1	-0.4	-0.6	-0.2	0.3	0.2	0.0	-0.3	-0.5	-0.3	-0.2	0.0	0.4	-0.3	-0.3	0.0	-0.1	-0.2	-0.1	0.5	0.6	-0.1	0.8		-0.2	-0.3	0.6	0.8	0.3	0.0	-0.1	-0.2	0.2	0.7	0.7	0.6	0.6	0.6	0.9	0.8	0.7	0.8	0.6
max IVCD	0.0	-0.1	0.1	0.0	-0.7	-0.5	-0.1	0.1	0.7	0.5	1.0	-0.9	-0.9	-0.9	0.6	0.7	0.5	1.0	-0.3	-0.9	-0.2	0.0	-0.5	-0.4	-0.1	0.8	-0.2	-0.4	0.8	0.1	-0.2		0.6	0.5	0.0	-0.2	-0.6	-0.2	-0.5	-0.7	-0.3	-0.6	-0.5	-0.7	-0.8	-0.1	-0.3	-0.3	-0.6	-0.7
max exp µv	-0.2	0.1	-0.2	-0.2	-0.6	-0.5	0.0	0.1	0.6	0.4	0.6	-0.7	-0.6	-0.5	0.3	0.5	0.3	0.6	-0.3	-0.6	0.1	0.1	-0.2	-0.4	-0.2	0.6	-0.6	-0.5	0.6	-0.1	-0.3	0.6		0.3	-0.2	0.2	-0.5	-0.1	-0.5	-0.8	-0.1	-0.7	-0.6	-0.7	-0.8	-0.1	-0.3	-0.6	-0.6	-0.7
mean exp µv	-0.3	0.0	-0.1	-0.2	-0.2	-0.3	0.1	-0.1	-0.1	-0.5	0.4	-0.5	-0.6	-0.6	0.3	0.2	-0.1	0.3	-0.4	-0.2	-0.5	-0.2	-0.2	-0.6	-0.3	0.5	0.1	0.1	0.5	0.9	0.6	0.5	0.3		0.8	0.3	-0.6	-0.3	-0.6	-0.5	0.4	0.3	0.3	0.1	-0.1	0.5	0.4	0.4	0.3	0.1
min exp µv	-0.3	-0.2	0.1	-0.1	0.1	0.0	0.1	-0.2	-0.3	-0.7	0.0	0.0	-0.1	-0.2	0.0	-0.2	-0.2	-0.1	-0.2	0.2	-0.5	-0.3	-0.1	-0.3	-0.2	0.1	0.4	0.4	0.1	0.9	0.8	0.0	-0.2	0.8		0.2	-0.2	-0.3	-0.4	0.0	0.6	0.6	0.6	0.5	0.4	0.7	0.7	0.6	0.7	0.5
max exp qGluc	-0.7	-0.1	-0.4	-0.2	0.1	0.1	-0.2	0.2	-0.2	-0.4	-0.2	0.1	0.1	0.1	-0.4	-0.2	0.1	-0.2	-0.5	0.1	0.1	-0.3	0.0	-0.2	-0.6	0.0	-0.2	0.2	0.0	0.3	0.3	-0.2	0.2	0.3	0.2		-0.2	-0.2	-0.2	-0.2	0.8	0.2	0.2	0.2	0.2	0.4	0.2	0.2	0.3	0.3
max exp qLac	0.2	0.3	0.0	0.6	0.5	0.3	0.0	0.1	-0.3	-0.1	-0.6	0.7	0.7	0.6	-0.3	-0.3	-0.1	-0.4	0.5	0.6	0.5	0.5	0.6	0.4	0.6	-0.4	0.5	0.4	-0.4	-0.2	0.0	-0.6	-0.5	-0.6	-0.2	-0.2		0.7	0.9	0.9	-0.1	0.4	0.4	0.5	0.6	-0.1	0.0	0.2	0.3	0.4
max exp qGlu	0.0	0.6	0.0	0.6	0.2	-0.1	0.2	-0.1	-0.1	0.1	-0.1	0.3	0.2	0.3	0.1	0.1	0.0	0.0	0.4	0.3	0.4	0.8	0.8	0.1	0.7	0.1	0.3	0.0	0.1	-0.1	-0.1	-0.2	-0.1	-0.3	-0.3	-0.2	0.7		0.7	0.4	-0.2	0.1	0.2	0.2	0.1	-0.2	-0.1	0.0	0.0	-0.1
max exp qGln	0.1	0.3	0.0	0.6	0.5	0.2	0.0	0.1	-0.3	0.0	-0.5	0.6	0.5	0.5	-0.3	-0.2	0.0	-0.4	0.3	0.5	0.4	0.5	0.6	0.2	0.5	-0.2	0.3	0.2	-0.2	-0.3	-0.2	-0.5	-0.5	-0.6	-0.4	-0.2	0.9	0.7		0.7	-0.2	0.3	0.3	0.4	0.4	-0.3	-0.1	0.1	0.1	0.2
max exp qNH4+	0.3	0.1	0.2	0.4	0.6	0.5	0.0	0.0	-0.5	-0.3	-0.7	0.9	0.8	0.7	-0.5	-0.6	-0.3	-0.6	0.6	0.8	0.3	0.2	0.5	0.5	0.5	-0.6	0.6	0.6	-0.6	0.0	0.2	-0.7	-0.8	-0.5	0.0	-0.2	0.9	0.4	0.7		0.0	0.6	0.5	0.7	0.8	0.1	0.3	0.3	0.5	0.7
mean exp qGluc	-0.6	-0.2	-0.3	-0.2	0.3	0.2	-0.1	0.0	-0.4	-0.6	-0.3	0.3	0.2	0.1	-0.4	-0.5	-0.1	-0.3	-0.3	0.3	0.0	-0.3	0.1	-0.1	-0.5	-0.2	0.2	0.3	-0.2	0.6	0.7	-0.3	-0.1	0.4	0.6	0.8	-0.1	-0.2	-0.2	0.0		0.6	0.5	0.5	0.5	0.8	0.6	0.6	0.7	0.6
mean exp qLac	-0.2	0.0	0.0	0.0	0.7	0.4	0.2	-0.1	-0.9	-0.8	-0.6	0.7	0.6	0.4	-0.4	-0.7	-0.6	-0.6	0.1	0.7	-0.2	0.0	0.4	-0.1	0.1	-0.4	0.6	0.7	-0.4	0.7	0.7	-0.6	-0.7	0.3	0.6	0.2	0.4	0.1	0.3	0.6	0.6		0.9	1.0	0.9	0.6	0.7	0.9	1.0	0.9
mean exp qGlu	-0.3	0.2	-0.1	0.1	0.6	0.3	0.3	0.0	-0.8	-0.8	-0.5	0.6	0.4	0.3	-0.2	-0.5	-0.6	-0.6	0.0	0.7	-0.2	0.2	0.6	-0.2	0.2	-0.2	0.5	0.5	-0.2	0.7	0.6	-0.5	-0.6	0.3	0.6	0.2	0.4	0.2	0.3	0.5	0.5	0.9		0.9	0.8	0.4	0.6	0.9	0.9	0.8
mean exp qGln	-0.2	0.1	-0.1	0.1	0.7	0.4	0.2	0.0	-0.9	-0.8	-0.7	0.8	0.7	0.5	-0.4	-0.6	-0.6	-0.8	0.1	0.8	-0.1	0.1	0.6	0.0	0.2	-0.4	0.5	0.6	-0.4	0.5	0.6	-0.7	-0.7	0.1	0.5	0.2	0.5	0.2	0.4	0.7	0.5	1.0	0.9		0.9	0.4	0.6	0.8	0.9	0.9
mean exp qNH4+	0.0	0.0	0.0	0.1	0.7	0.5	0.0	0.1	-0.8	-0.8	-0.8	0.9	0.8	0.6	-0.5	-0.7	-0.5	-0.7	0.3	0.8	0.0	-0.1	0.4	0.2	0.2	-0.6	0.6	0.7	-0.6	0.4	0.6	-0.8	-0.8	-0.1	0.4	0.2	0.6	0.1	0.4	0.8	0.5	0.9	0.8	0.9		0.4	0.5	0.6	0.8	1.0
min exp qGluc	-0.4	-0.3	0.1	-0.3	0.2	0.2	0.1	-0.1	-0.3	-0.4	-0.2	0.2	0.2	0.1	-0.4	-0.5	-0.2	-0.2	-0.1	0.2	-0.1	-0.3	-0.1	0.0	-0.4	-0.1	0.4	0.3	-0.1	0.6	0.9	-0.1	-0.1	0.5	0.7	0.4	-0.1	-0.2	-0.3	0.1	0.8	0.6	0.4	0.4	0.4		0.9	0.7	0.7	0.4
min exp qLac	-0.3	-0.2	0.2	-0.2	0.3	0.3	0.3	-0.2	-0.5	-0.5	-0.3	0.3	0.3	0.1	-0.4	-0.5	-0.4	-0.3	0.1	0.3	-0.2	-0.2	0.0	-0.1	-0.2	-0.1	0.5	0.5	-0.1	0.6	0.8	-0.3	-0.3	0.4	0.7	0.2	0.0	-0.1	-0.1	0.3	0.6	0.7	0.6	0.6	0.5	0.9		0.8	0.8	0.5
min exp qGlu	-0.3	-0.1	0.2	0.0	0.5	0.2	0.3	0.1	-0.7	-0.7	-0.4	0.4	0.3	0.2	-0.3	-0.5	-0.4	-0.4	-0.1	0.4	-0.2	0.0	0.2	-0.2	0.0	-0.2	0.5	0.4	-0.2	0.7	0.7	-0.3	-0.6	0.4	0.6	0.2	0.2	0.0	0.1	0.3	0.6	0.9	0.9	0.8	0.6	0.7	0.8		0.9	0.7
min exp qGln	-0.3	-0.1	0.0	-0.1	0.6	0.4	0.3	-0.1	-0.8	-0.8	-0.6	0.6	0.6	0.4	-0.4	-0.7	-0.6	-0.6	0.1	0.7	-0.1	-0.1	0.4	0.0	0.0	-0.4	0.6	0.6	-0.4	0.7	0.8	-0.6	-0.6	0.3	0.7	0.3	0.3	0.0	0.1	0.5	0.7	1.0	0.9	0.9	0.8	0.7	0.8	0.9		0.9
min exp qNH4+	-0.1	-0.1	0.0	-0.1	0.7	0.5	0.0	0.2	-0.8	-0.8	-0.8	0.8	0.7	0.5	-0.6	-0.8	-0.5	-0.7	0.2	0.8	0.0	-0.2	0.2	0.1	0.0	-0.6	0.5	0.7	-0.6	0.5	0.6	-0.7	-0.7	0.1	0.5	0.3	0.4	-0.1	0.2	0.7	0.6	0.9	0.8	0.9	1.0	0.4	0.5	0.7	0.9	

B. ActiPro

	CB1	CB2	CB3	CB4	CB5	CB6	CB7a	CB7b	max time	max time exp	max pCC	max diam	mean diam	min_diam	max circ	mean circ	min circ	pTiter	max Gluc	first Gluc below 3000	max Lac	max Glu	max Gln	max NH4+	max. osmolality	max. qP	mean qP	min qP	max exp qP	mean exp qP	min exp qP	Max. IVCD	max exp µv	mean exp µv	min exp µv	max exp qGluc	max exp qLac	max exp qGlu cons.	max exp qGln	max exp qNH4+	mean exp qGluc	mean exp qLac	mean exp qGlu	mean exp qGln	mean exp qNH4+	min exp qGluc	min exp qLac	min exp qGlu	min exp qGln	min exp qNH4+
CB1		0.0	0.0	0.0	0.0	0.0	0.0	0.0	0.2	0.2	0.3	-0.1	-0.1	-0.2	0.3	0.3	0.1	0.3	0.7	0.0	0.1	0.0	-0.2	0.5	0.5	0.0	-0.2	-0.4	0.0	0.0	0.2	0.2	-0.1	0.0	-0.1	-0.4	0.0	-0.1	0.0	0.0	-0.5	-0.2	-0.3	-0.2	-0.1	-0.6	-0.2	-0.3	-0.1	0.2
CB2	0.0		0.0	0.0	0.0	0.0	0.0	0.0	0.0	-0.1	0.0	0.1	0.1	0.0	0.1	0.0	-0.1	-0.1	0.3	0.3	0.0	0.6	0.4	0.2	0.5	0.5	0.1	0.1	0.5	0.1	0.1	-0.1	0.0	-0.1	-0.1	-0.1	0.2	0.5	-0.4	0.2	0.1	0.3	0.3	-0.2	0.3	0.0	0.0	-0.3	-0.1	0.4
CB3	0.0	0.0		0.0	0.0	0.0	0.0	0.0	0.0	0.1	0.2	0.1	-0.1	-0.4	0.1	0.4	0.2	0.2	0.4	-0.1	0.0	-0.1	-0.2	0.1	0.0	-0.1	0.1	0.1	-0.1	0.3	0.4	0.3	-0.2	0.0	-0.1	-0.5	-0.2	-0.2	-0.1	0.1	-0.4	-0.2	-0.3	-0.2	-0.1	-0.1	0.2	0.0	-0.1	-0.1
CB4	0.0	0.0	0.0		0.0	0.0	0.0	0.0	0.0	-0.1	-0.1	0.3	0.3	0.1	0.2	0.5	0.4	0.0	0.2	0.2	0.0	0.4	0.4	0.1	0.5	0.5	0.3	0.2	0.5	0.3	-0.1	-0.1	-0.3	-0.1	-0.1	0.4	0.5	0.5	-0.2	0.3	0.2	0.3	0.2	0.1	0.4	-0.2	0.3	-0.3	-0.1	0.5
CB5	0.0	0.0	0.0	0.0		0.0	0.0	0.0	0.1	-0.1	-0.5	0.6	0.6	0.5	-0.4	-0.2	0.2	-0.3	0.1	0.4	-0.5	0.0	0.3	-0.4	0.0	-0.3	0.4	0.4	-0.3	-0.1	-0.1	-0.6	-0.6	-0.6	-0.4	0.1	0.3	-0.1	-0.1	0.4	0.1	0.1	-0.5	0.4	0.2	-0.1	0.3	-0.5	0.6	-0.2
CB6	0.0	0.0	0.0	0.0	0.0		0.0	0.0	-0.1	0.0	-0.4	0.6	0.5	0.5	-0.5	0.0	0.3	-0.4	0.1	0.4	-0.3	0.0	0.1	0.0	0.0	-0.2	0.4	0.4	-0.2	0.1	0.1	-0.4	-0.6	-0.5	-0.1	0.3	0.5	0.1	0.6	0.6	0.2	0.3	-0.2	0.6	0.6	-0.1	0.1	-0.4	-0.1	0.4
CB7a	0.0	0.0	0.0	0.0	0.0	0.0		0.0	0.0	-0.1	-0.3	0.3	0.4	0.3	-0.2	0.2	0.5	-0.2	0.3	0.6	-0.4	0.6	0.7	-0.1	0.4	0.3	0.4	0.3	0.3	0.2	0.1	-0.3	-0.2	-0.3	-0.3	0.2	0.3	0.5	0.0	0.3	0.2	0.4	0.3	0.1	0.4	-0.1	-0.3	-0.3	0.0	0.2
CB7b	0.0	0.0	0.0	0.0	0.0	0.0	0.0		0.2	0.1	0.1	0.0	0.0	-0.1	-0.1	-0.1	0.3	0.1	0.0	0.0	0.4	0.1	0.0	0.2	0.1	-0.2	-0.2	0.0	-0.2	-0.3	-0.2	0.2	0.0	-0.1	-0.2	0.1	0.0	-0.1	-0.1	0.0	-0.2	-0.1	-0.2	-0.2	0.0	-0.3	-0.2	-0.2	-0.1	0.1
max time	0.2	0.0	0.0	0.0	0.1	-0.1	0.0	0.2		0.7	0.5	0.1	-0.3	-0.3	-0.2	-0.3	0.0	0.8	0.3	-0.3	0.5	0.0	0.1	0.5	0.1	-0.2	-0.5	-0.5	-0.2	-0.6	-0.5	0.4	-0.1	-0.4	-0.6	0.1	0.0	-0.3	0.0	0.0	-0.5	-0.5	-0.5	-0.2	-0.4	-0.5	-0.4	0.0	0.3	-0.1
max time exp	0.2	-0.1	0.1	-0.1	-0.1	0.0	-0.1	0.1	0.7		0.5	-0.1	-0.4	-0.4	0.0	0.0	-0.1	0.6	0.2	-0.4	0.5	-0.2	-0.2	0.6	-0.1	-0.2	-0.5	-0.6	-0.2	-0.8	-0.6	0.4	0.0	-0.4	-0.7	0.0	-0.1	-0.4	0.2	0.0	-0.7	-0.7	-0.5	-0.4	-0.4	-0.6	-0.7	0.1	-0.2	-0.1
max pCC	0.3	0.0	0.2	-0.1	-0.5	-0.4	-0.3	0.1	0.5	0.5		-0.8	-1.0	-0.9	0.6	0.0	-0.4	0.9	0.1	-0.8	0.9	-0.3	-0.5	0.7	-0.1	-0.1	-0.9	-0.9	-0.1	-0.4	-0.1	1.0	0.6	0.5	0.1	-0.5	-0.7	-0.4	-0.2	-0.7	-0.7	-0.8	-0.2	-0.7	-0.8	-0.2	-0.4	0.6	-0.2	-0.3
max diam	-0.1	0.1	0.1	0.3	0.6	0.6	0.3	0.0	0.1	-0.1	-0.8		0.9	0.8	-0.7	0.0	0.6	-0.5	0.4	0.8	-0.7	0.4	0.6	-0.3	0.3	0.0	0.8	0.7	0.0	0.1	0.0	-0.7	-0.9	-0.9	-0.6	0.5	0.8	0.3	0.2	0.9	0.4	0.6	-0.2	0.6	0.8	-0.2	0.2	-0.8	0.3	0.4
mean diam	-0.1	0.1	-0.1	0.3	0.6	0.5	0.4	0.0	-0.3	-0.4	-1.0	0.9		0.9	-0.7	0.0	0.6	-0.8	0.2	0.9	-0.8	0.4	0.7	-0.5	0.3	0.1	0.9	0.9	0.1	0.3	0.1	-0.9	-0.8	-0.7	-0.3	0.5	0.8	0.5	0.2	0.8	0.6	0.8	0.1	0.7	0.9	0.0	0.2	-0.8	0.3	0.4
min_diam	-0.2	0.0	-0.4	0.1	0.5	0.5	0.3	-0.1	-0.3	-0.4	-0.9	0.8	0.9		-0.7	-0.2	0.4	-0.8	-0.1	0.8	-0.8	0.3	0.6	-0.6	0.1	0.0	0.8	0.8	0.0	0.2	0.0	-0.9	-0.6	-0.6	-0.2	0.6	0.7	0.3	0.3	0.7	0.7	0.8	0.1	0.7	0.8	0.2	0.3	-0.6	0.4	0.3
max circ	0.3	0.1	0.1	0.2	-0.4	-0.5	-0.2	-0.1	-0.2	0.0	0.6	-0.7	-0.7	-0.7		0.4	-0.3	0.4	0.0	-0.5	0.5	0.0	-0.3	0.5	0.2	0.3	-0.5	-0.6	0.3	0.1	0.1	0.6	0.6	0.7	0.4	-0.6	-0.5	0.0	-0.5	-0.6	-0.4	-0.4	0.2	-0.6	-0.5	0.0	0.0	0.4	-0.4	-0.1
mean circ	0.3	0.0	0.4	0.5	-0.2	0.0	0.2	-0.1	-0.3	0.0	0.0	0.0	0.0	-0.2	0.4		0.5	0.0	0.4	0.2	0.0	0.3	0.1	0.3	0.4	0.5	0.2	0.0	0.5	0.4	0.2	0.1	-0.1	0.1	-0.1	-0.2	0.1	0.3	-0.3	0.2	-0.1	0.2	0.2	-0.2	0.2	0.0	-0.1	-0.1	-0.4	0.5
min circ	0.1	-0.1	0.2	0.4	0.2	0.3	0.5	0.3	0.0	-0.1	-0.4	0.6	0.6	0.4	-0.3	0.5		-0.2	0.4	0.7	-0.3	0.4	0.4	0.0	0.5	0.2	0.5	0.5	0.2	0.3	0.1	-0.3	-0.6	-0.5	-0.4	0.3	0.6	0.4	-0.2	0.6	0.2	0.5	0.1	0.1	0.7	-0.1	0.1	-0.6	-0.1	0.6
pTiter	0.3	-0.1	0.2	0.0	-0.3	-0.4	-0.2	0.1	0.8	0.6	0.9	-0.5	-0.8	-0.8	0.4	0.0	-0.2		0.2	-0.6	0.8	-0.1	-0.3	0.6	0.1	-0.1	-0.7	-0.8	-0.1	-0.4	-0.2	0.9	0.3	0.2	-0.2	-0.3	-0.4	-0.4	-0.1	-0.5	-0.7	-0.7	-0.3	-0.5	-0.7	-0.4	-0.3	0.4	0.0	-0.2
max Gluc	0.7	0.3	0.4	0.2	0.1	0.1	0.3	0.0	0.3	0.2	0.1	0.4	0.2	-0.1	0.0	0.4	0.4	0.2		0.4	-0.1	0.5	0.3	0.5	0.8	0.3	0.2	0.0	0.3	0.2	0.3	0.0	-0.5	-0.4	-0.4	-0.2	0.4	0.2	-0.1	0.4	-0.3	0.0	-0.2	0.0	0.3	-0.6	-0.2	-0.6	0.0	0.5
first Gluc below 3000	0.0	0.3	-0.1	0.2	0.4	0.4	0.6	0.0	-0.3	-0.4	-0.8	0.8	0.9	0.8	-0.5	0.2	0.7	-0.6	0.4		-0.8	0.7	0.7	-0.3	0.6	0.4	0.9	0.8	0.4	0.4	0.2	-0.8	-0.7	-0.6	-0.3	0.4	0.8	0.7	0.0	0.8	0.6	0.8	0.3	0.4	0.9	-0.1	0.2	-0.8	0.1	0.6
max Lac	0.1	0.0	0.0	0.0	-0.5	-0.3	-0.4	0.4	0.5	0.5	0.9	-0.7	-0.8	-0.8	0.5	0.0	-0.3	0.8	-0.1	-0.8		-0.2	-0.4	0.7	-0.1	0.0	-0.9	-0.8	0.0	-0.5	-0.4	0.9	0.5	0.3	0.0	-0.2	-0.5	-0.3	-0.2	-0.6	-0.6	-0.7	-0.2	-0.6	-0.7	-0.1	-0.4	0.5	-0.2	-0.1
max Glu	0.0	0.6	-0.1	0.4	0.0	0.0	0.6	0.1	0.0	-0.2	-0.3	0.4	0.4	0.3	0.0	0.3	0.4	-0.1	0.5	0.7	-0.2		0.9	0.2	0.8	0.7	0.4	0.3	0.7	0.2	0.0	-0.2	-0.3	-0.3	-0.3	0.3	0.6	0.8	-0.2	0.5	0.3	0.6	0.5	0.0	0.5	-0.1	-0.2	-0.5	-0.1	0.6
max Gln	-0.2	0.4	-0.2	0.4	0.3	0.1	0.7	0.0	0.1	-0.2	-0.5	0.6	0.7	0.6	-0.3	0.1	0.4	-0.3	0.3	0.7	-0.4	0.9		-0.1	0.6	0.6	0.6	0.4	0.6	0.2	-0.1	-0.5	-0.4	-0.5	-0.4	0.5	0.7	0.7	-0.1	0.6	0.4	0.6	0.3	0.3	0.6	0.0	-0.1	-0.6	0.2	0.4
max NH4+	0.5	0.2	0.1	0.1	-0.4	0.0	-0.1	0.2	0.5	0.6	0.7	-0.3	-0.5	-0.6	0.5	0.3	0.0	0.6	0.5	0.3	0.7	0.2	-0.1		0.4	0.3	-0.5	-0.6	0.3	-0.3	-0.2	0.6	0.1	0.0	-0.3	-0.2	-0.1	-0.1	-0.2	-0.1	-0.6	-0.4	-0.1	-0.4	-0.3	-0.3	-0.5	0.1	-0.3	0.3
max. osmolality	0.5	0.5	0.0	0.5	0.0	0.0	0.4	0.1	0.1	-0.1	-0.1	0.3	0.3	0.1	0.2	0.4	0.5	0.1	0.8	0.6	-0.1	0.8	0.6	0.4		0.7	0.3	0.1	0.7	0.3	0.2	-0.1	-0.3	-0.3	-0.3	0.1	0.6	0.6	-0.3	0.4	0.0	0.4	0.2	-0.1	0.4	-0.4	-0.1	-0.6	-0.1	0.7
max. qP	0.0	0.5	-0.1	0.5	-0.3	-0.2	0.3	-0.2	-0.2	-0.2	-0.1	0.0	0.1	0.0	0.3	0.5	0.2	-0.1	0.3	0.4	0.0	0.7	0.6	0.3	0.7		0.3	0.0	1.0	0.3	0.0	-0.1	0.1	0.0	-0.1	0.2	0.5	0.8	-0.2	0.3	0.2	0.4	0.7	-0.1	0.3	0.1	-0.2	-0.1	-0.3	0.6
mean qP	-0.2	0.1	0.1	0.3	0.4	0.4	0.4	-0.2	-0.5	-0.5	-0.9	0.8	0.9	0.8	-0.5	0.2	0.5	-0.7	0.2	0.9	-0.9	0.4	0.6	-0.5	0.3	0.3		0.9	0.3	0.7	0.4	-0.9	-0.7	-0.4	-0.1	0.4	0.7	0.5	0.1	0.8	0.6	0.8	0.2	0.6	0.8	0.1	0.4	-0.7	0.2	0.3
min qP	-0.4	0.1	0.1	0.2	0.4	0.4	0.3	0.0	-0.5	-0.6	-0.9	0.7	0.9	0.8	-0.6	0.0	0.5	-0.8	0.0	0.8	-0.8	0.3	0.4	-0.6	0.1	0.0	0.9		0.0	0.6	0.4	-0.8	-0.7	-0.4	0.0	0.4	0.6	0.4	0.1	0.7	0.7	0.8	0.2	0.6	0.8	0.3	0.6	-0.6	0.1	0.3
max exp qP	0.0	0.5	-0.1	0.5	-0.3	-0.2	0.3	-0.2	-0.2	-0.2	-0.1	0.0	0.1	0.0	0.3	0.5	0.2	-0.1	0.3	0.4	0.0	0.7	0.6	0.3	0.7	1.0	0.3	0.0		0.3	0.0	-0.1	0.1	0.0	-0.1	0.2	0.5	0.8	-0.2	0.3	0.2	0.4	0.7	-0.1	0.3	0.1	-0.2	-0.1	-0.3	0.6
mean exp qP	0.0	0.1	0.3	0.3	-0.1	0.1	0.2	-0.3	-0.6	-0.8	-0.4	0.1	0.3	0.2	0.1	0.4	0.3	-0.4	0.2	0.4	-0.5	0.2	0.2	-0.3	0.3	0.3	0.7	0.6	0.3		0.8	-0.3	-0.2	0.3	0.5	-0.1	0.2	0.4	-0.1	0.2	0.5	0.6	0.4	0.3	0.5	0.4	0.6	-0.2	0.0	0.2
min exp qP	0.2	0.1	0.4	-0.1	-0.1	0.1	0.1	-0.2	-0.5	-0.6	-0.1	0.0	0.1	0.0	0.1	0.2	0.1	-0.2	0.3	0.2	-0.4	0.0	-0.1	-0.2	0.2	0.0	0.4	0.4	0.0	0.8		-0.1	-0.1	0.4	0.6	-0.3	-0.1	0.1	-0.1	0.0	0.2	0.3	0.2	0.1	0.2	0.2	0.6	-0.2	-0.1	0.0
max IVCD	0.2	-0.1	0.3	-0.1	-0.6	-0.4	-0.3	0.2	0.4	0.4	1.0	-0.7	-0.9	-0.9	0.6	0.1	-0.3	0.9	0.0	-0.8	0.9	-0.2	-0.5	0.6	-0.1	-0.1	-0.9	-0.8	-0.1	-0.3	-0.1		0.6	0.5	0.1	-0.4	-0.6	-0.4	-0.2	-0.7	-0.7	-0.7	-0.1	-0.7	-0.8	-0.1	-0.3	0.6	-0.2	-0.2
max exp µv	-0.1	0.0	-0.2	-0.3	-0.6	-0.6	-0.2	0.0	-0.1	0.0	0.6	-0.9	-0.8	-0.6	0.6	-0.1	-0.6	0.3	-0.5	-0.7	0.5	-0.3	-0.4	0.1	-0.3	0.1	-0.7	-0.7	0.1	-0.2	-0.1	0.6		0.8	0.5	-0.4	-0.7	-0.2	-0.3	-0.8	-0.2	-0.4	0.4	-0.6	-0.7	0.3	-0.2	0.8	-0.3	-0.3
mean exp µv	0.0	-0.1	0.0	-0.1	-0.6	-0.5	-0.3	-0.1	-0.4	-0.4	0.5	-0.9	-0.7	-0.6	0.7	0.1	-0.5	0.2	-0.4	-0.6	0.3	-0.3	-0.5	0.0	-0.3	0.0	-0.4	-0.4	0.0	0.3	0.4	0.5	0.8		0.9	-0.5	-0.7	-0.2	-0.3	-0.8	0.0	-0.2	0.4	-0.4	-0.5	0.4	0.2	0.7	-0.2	-0.3
min exp µv	-0.1	-0.1	-0.1	-0.1	-0.4	-0.1	-0.3	-0.2	-0.6	-0.7	0.1	-0.6	-0.3	-0.2	0.4	-0.1	-0.4	-0.2	-0.4	-0.3	0.0	-0.3	-0.4	-0.3	-0.3	-0.1	-0.1	0.0	-0.1	0.5	0.6	0.1	0.5	0.9		-0.3	-0.5	-0.1	-0.1	-0.6	0.3	0.1	0.3	0.0	-0.2	0.5	0.5	0.4	-0.1	-0.3
max exp qGluc	-0.4	-0.1	-0.5	0.4	0.1	0.3	0.2	0.1	0.1	0.0	-0.5	0.5	0.5	0.6	-0.6	-0.2	0.3	-0.3	-0.2	0.4	-0.2	0.3	0.5	-0.2	0.1	0.2	0.4	0.4	0.2	-0.1	-0.3	-0.4	-0.4	-0.5	-0.3		0.7	0.4	0.4	0.5	0.6	0.5	0.2	0.5	0.5	0.1	0.0	-0.3	0.2	0.3
max exp qLac	0.0	0.2	-0.2	0.5	0.3	0.5	0.3	0.0	0.0	-0.1	-0.7	0.8	0.8	0.7	-0.5	0.1	0.6	-0.4	0.4	0.8	-0.5	0.6	0.7	-0.1	0.6	0.5	0.7	0.6	0.5	0.2	-0.1	-0.6	-0.7	-0.7	-0.5	0.7		0.7	0.2	0.9	0.5	0.7	0.2	0.5	0.9	-0.1	0.1	-0.7	0.1	0.7
max exp qGlu cons.	-0.1	0.5	-0.2	0.5	-0.1	0.1	0.5	-0.1	-0.3	-0.4	-0.4	0.3	0.5	0.3	0.0	0.3	0.4	-0.4	0.2	0.7	-0.3	0.8	0.7	-0.1	0.6	0.8	0.5	0.4	0.8	0.4	0.1	-0.4	-0.2	-0.2	-0.1	0.4	0.7		-0.1	0.5	0.5	0.7	0.7	0.1	0.6	0.1	0.1	-0.4	-0.2	0.6
max exp qGln	0.0	-0.4	-0.1	-0.2	-0.1	0.6	0.0	-0.1	0.0	0.2	-0.2	0.2	0.2	0.3	-0.5	-0.3	-0.2	-0.1	-0.1	0.0	-0.2	-0.2	-0.1	-0.2	-0.3	-0.2	0.1	0.1	-0.2	-0.1	-0.1	-0.2	-0.3	-0.3	-0.1	0.4	0.2	-0.1		0.3	0.1	0.0	-0.3	0.6	0.1	-0.1	-0.2	0.0	0.1	0.0
max exp qNH4+	0.0	0.2	0.1	0.3	0.4	0.6	0.3	0.0	0.0	0.0	-0.7	0.9	0.8	0.7	-0.6	0.2	0.6	-0.5	0.4	0.8	-0.6	0.5	0.6	-0.1	0.4	0.3	0.8	0.7	0.3	0.2	0.0	-0.7	-0.8	-0.8	-0.6	0.5	0.9	0.5	0.3		0.4	0.6	0.0	0.5	0.9	-0.2	0.1	-0.7	0.0	0.6
mean exp qGluc	-0.5	0.1	-0.4	0.2	0.1	0.2	0.2	-0.2	-0.5	-0.7	-0.7	0.4	0.6	0.7	-0.4	-0.1	0.2	-0.7	-0.3	0.6	-0.6	0.3	0.4	-0.6	0.0	0.2	0.6	0.7	0.2	0.5	0.2	-0.7	-0.2	0.0	0.3	0.6	0.5	0.5	0.1	0.4		0.9	0.5	0.6	0.7	0.6	0.5	-0.1	0.3	0.3
mean exp qLac	-0.2	0.3	-0.2	0.3	0.1	0.3	0.4	-0.1	-0.5	-0.7	-0.8	0.6	0.8	0.8	-0.4	0.2	0.5	-0.7	0.0	0.8	-0.7	0.6	0.6	-0.4	0.4	0.4	0.8	0.8	0.4	0.6	0.3	-0.7	-0.4	-0.2	0.1	0.5	0.7	0.7	0.0	0.6	0.9		0.6	0.4	0.9	0.4	0.4	-0.5	0.0	0.6
mean exp qGlu	-0.3	0.3	-0.3	0.2	-0.5	-0.2	0.3	-0.2	-0.5	-0.5	-0.2	-0.2	0.1	0.1	0.2	0.2	0.1	-0.3	-0.2	0.3	-0.2	0.5	0.3	-0.1	0.2	0.7	0.2	0.2	0.7	0.4	0.2	-0.1	0.4	0.4	0.3	0.2	0.2	0.7	-0.3	0.0	0.5	0.6		-0.2	0.3	0.4	0.1	0.2	-0.5	0.4
mean exp qGln	-0.2	-0.2	-0.2	0.1	0.4	0.6	0.1	-0.2	-0.2	-0.4	-0.7	0.6	0.7	0.7	-0.6	-0.2	0.1	-0.5	0.0	0.4	-0.6	0.0	0.3	-0.4	-0.1	-0.1	0.6	0.6	-0.1	0.3	0.1	-0.7	-0.6	-0.4	0.0	0.5	0.5	0.1	0.6	0.5	0.6	0.4	-0.2		0.5	0.2	0.3	-0.3	0.6	0.1
mean exp qNH4+	-0.1	0.3	-0.1	0.4	0.2	0.6	0.4	0.0	-0.4	-0.4	-0.8	0.8	0.9	0.8	-0.5	0.2	0.7	-0.7	0.3	0.9	-0.7	0.5	0.6	-0.3	0.4	0.3	0.8	0.8	0.3	0.5	0.2	-0.8	-0.7	-0.5	-0.2	0.5	0.9	0.6	0.1	0.9	0.7	0.9	0.3	0.5		0.1	0.3	-0.7	0.0	0.7
min exp qGluc	-0.6	0.0	-0.1	-0.2	-0.1	-0.1	-0.1	-0.3	-0.5	-0.6	-0.2	-0.2	0.0	0.2	0.0	0.0	-0.1	-0.4	-0.6	-0.1	-0.1	-0.1	0.0	-0.3	-0.4	0.1	0.1	0.3	0.1	0.4	0.2	-0.1	0.3	0.4	0.5	0.1	-0.1	0.1	-0.1	-0.2	0.6	0.4	0.4	0.2	0.1		0.3	0.5	0.2	-0.1
min exp qLac	-0.2	0.0	0.2	0.3	0.3	0.1	-0.3	-0.2	-0.4	-0.7	-0.4	0.2	0.2	0.3	0.0	-0.1	0.1	-0.3	-0.2	0.2	-0.4	-0.2	-0.1	-0.5	-0.1	-0.2	0.4	0.6	-0.2	0.6	0.6	-0.3	-0.2	0.2	0.5	0.0	0.1	0.1	-0.2	0.1	0.5	0.4	0.1	0.3	0.3	0.3		-0.1	0.2	0.0
min exp qGlu	-0.3	-0.3	0.0	-0.3	-0.5	-0.4	-0.3	-0.2	0.0	0.1	0.6	-0.8	-0.8	-0.6	0.4	-0.1	-0.6	0.4	-0.6	-0.8	0.5	-0.5	-0.6	0.1	-0.6	-0.1	-0.7	-0.6	-0.1	-0.2	-0.2	0.6	0.8	0.7	0.4	-0.3	-0.7	-0.4	0.0	-0.7	-0.1	-0.5	0.2	-0.3	-0.7	0.5	-0.1		-0.1	-0.5
min exp qGln	-0.1	-0.1	-0.1	-0.1	0.6	-0.1	0.0	-0.1	0.3	-0.2	-0.2	0.3	0.3	0.4	-0.4	-0.4	-0.1	0.0	0.0	0.1	-0.2	-0.1	0.2	-0.3	-0.1	-0.3	0.2	0.1	-0.3	0.0	-0.1	-0.2	-0.3	-0.2	-0.1	0.2	0.1	-0.2	0.1	0.0	0.3	0.0	-0.5	0.6	0.0	0.2	0.2	-0.1		-0.2
min exp qNH4+	0.2	0.4	-0.1	0.5	-0.2	0.4	0.2	0.1	-0.1	-0.1	-0.3	0.4	0.4	0.3	-0.1	0.5	0.6	-0.2	0.5	0.6	-0.1	0.6	0.4	0.3	0.7	0.6	0.3	0.3	0.6	0.2	0.0	-0.2	-0.3	-0.3	-0.3	0.3	0.7	0.6	0.0	0.6	0.3	0.6	0.4	0.1	0.7	-0.1	0.0	-0.5	-0.2	

Supplementary Information 4 PLS regression model qualities generated from DoE1 spiked batch cultures for CDM4NS0 (A.) or ActiPro (B.).

A. CDM4NS0

	R^2	Q^2
max time	0.812969	0.515249
max exp time	0.426684	0.143034
max pCC	0.957207	0.885265
max diam	0.908675	0.466644
mean diam	0.835685	0.793585
min diam	0.644572	0.0490317
max circ	0.76137	0.394029
mean circ	0.852367	0.372562
min circ	0.750981	0.298917
pTiter_OctetRed	0.942054	0.631753
max Gluc	0.66363	-0.169657
first Gluc limit	0.827916	0.350385
max Lac	0.431922	-0.115286
max Glu	0.952595	0.673943
max Gln	0.981607	0.726658
max NH4	0.366107	-0.2
max osm.	0.976067	0.764059
max qP_OctetRed	0.609994	-0.0376735
mean qP_OctetRed	0.46158	-0.2
min qP_OctetRed	0.419452	-0.177532
max exp qP_OctetRed	0.609994	-0.0376735
mean exp qP_OctetRed	0.205125	-0.2
min exp qP_OctetRed	0.313525	-0.2
Max. IVCD	0.961577	0.678165
max exp µv	0.686288	0.52717
mean exp µv	0.185809	0.0621897
min exp µv	0.199185	-0.2
max exp qGluc	0.908968	0.602289
max exp qLac	0.86174	0.561874
max exp qGlu	0.76689	0.461718
max exp qGln	0.735241	0.442792
max exp qNH4	0.93036	0.584177
mean exp qGluc	0.752233	0.633718
mean exp qLac	0.627784	0.586218
mean exp qGlu	0.633301	0.380519
mean exp qGln	0.752411	0.670133
mean exp qNH4	0.796988	0.757995
min exp qGluc	0.482389	-0.2
min exp qLac	0.523825	-0.2
min exp qGlu	0.538291	-0.170337
min exp qGln	0.681376	0.185333
min exp NH4	0.766405	0.428806

B. ActiPro

	R^2	Q^2
max exp time	0.0557511	0.05
max pCC	0.699288	0.493559
max diam	0.913011	0.584461
mean diam	0.823772	0.669371
min diam	0.825225	0.471868
max circ	0.66853	0.0920409
mean circ	0.585472	-0.2
min circ	0.709157	0.139798
pTiter OctetRed	0.569853	0.280363
max Gluc	0.929019	0.614439
first Gluc limit	0.82219	0.482857
max Lac	0.644423	0.225552
max Glu	0.977412	0.721754
max Gln	0.914384	0.609655
max NH4	0.506947	0.0207752
max osm.	0.966054	0.735363
max qP OctetRed	0.785482	0.482381
mean qP OctetRed	0.697076	0.326058
min qP OctetRed	0.667348	0.440907
max exp qP OctetRed	0.785482	0.482381
mean exp qP OctetRed	0.319768	-0.12251
min exp qP OctetRed	0.198103	0.0891415
max IVCD	0.755426	0.493482
max exp µv	0.892738	0.774825
mean exp µv	0.668037	0.338766
min exp µv	0.330827	-0.2
max exp qGluc	0.664641	0.0977428
max exp qLac	0.748006	0.41471
max exp qGlu	0.816312	0.775867
max exp qGln	0.496838	0.384606
max exp qNH4	0.785382	0.605319
mean exp qGluc	0.384467	0.247789
mean exp qLac	0.525251	0.258179
mean exp qGlu	0.757487	0.446553
mean exp qGln	0.552935	0.421892
mean exp qNH4	0.761855	0.549193
min exp qGluc	0.501354	-0.164525
min exp qLac	0.422013	-0.0110143
min exp qGlu	0.893018	0.626114
min exp qGln	0.399161	-0.2
min exp NH4	0.631491	0.461522
STY OctetRed	0.786405	0.388195
pVCD	0.845077	0.518904

Supplementary Information 5 PLS linear (main) coefficients for DoE1 spiked batch experiments in CDM4NS0 (A.) or ActiPro (B.).

'~' represents transformed data sets.

A. CDM4NS0

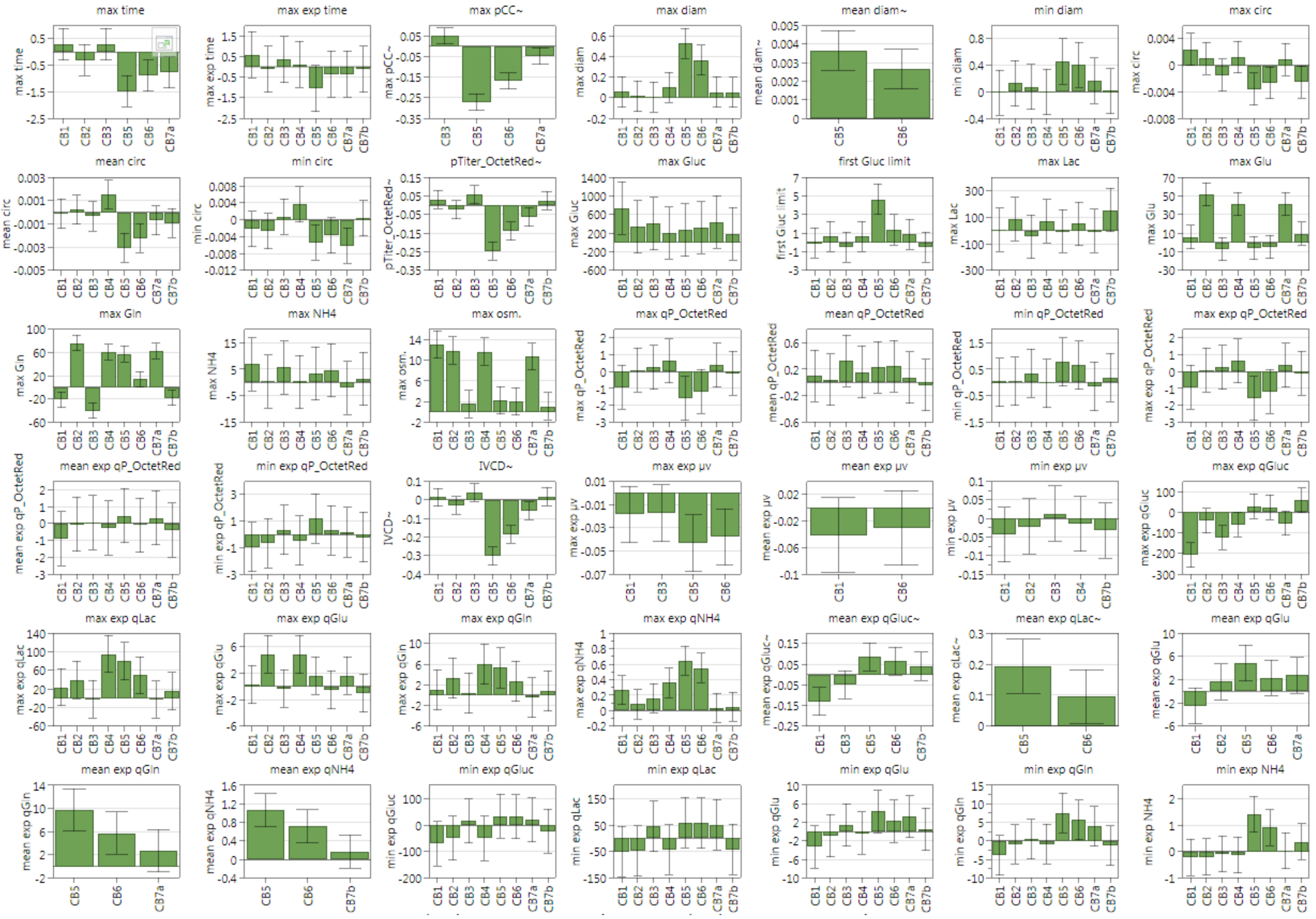

B. ActiPro

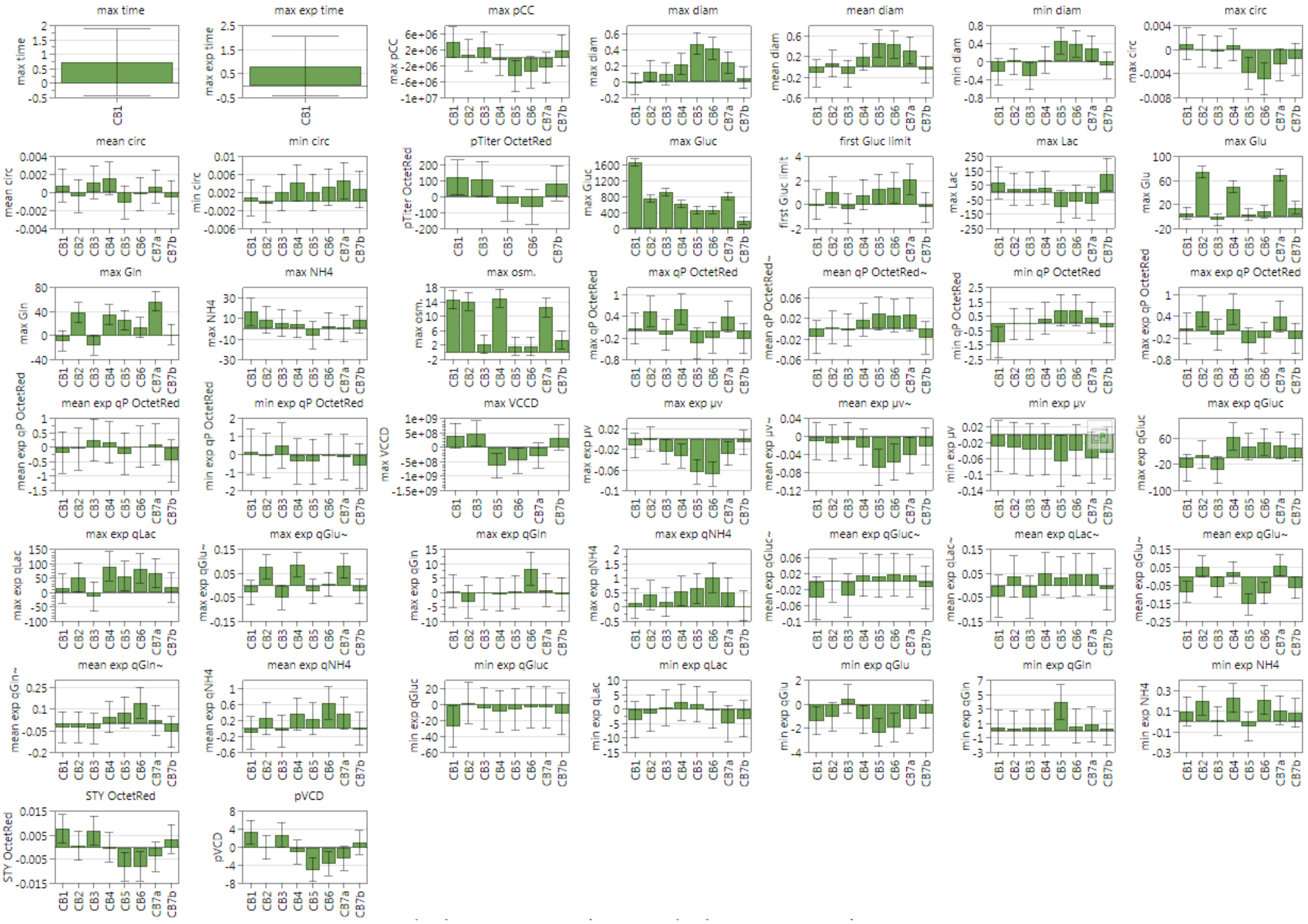
max time
max exp time
max pCC
max diam
mean diam
min diam
max circ
mean circ
min circ
pTiter OctetRed
max Gluc
first Gluc limit
max Lac
max Glu
max Gln
max NH4
max osm.
max qP OctetRed
mean qP OctetRed~
min qP OctetRed
max exp qP OctetRed
mean exp qP OctetRed
min exp qP OctetRed
max VCCD
max exp µv
mean exp µv~
min exp µv
max exp qGluc
max exp qLac
max exp qGlu~
max exp qGln
max exp qNH4
mean exp qGluc~
mean exp qLac~
mean exp qGlu~
mean exp qGln~
mean exp qNH4
min exp qGluc
min exp qLac
min exp qGlu
min exp qGln
min exp NH4
STY OctetRed
pVCD

Supplementary Information 6 Graphical representation of semi-continuous perfusion culture data from the optimization design (DoE2). *CDM4NS0 (A., left panels) or ActiPro basal media (B., right panels) were spiked with different combinations of CB1, 3, 7a and 7b according to the DoE2 matrix (Figure 3A and C).*

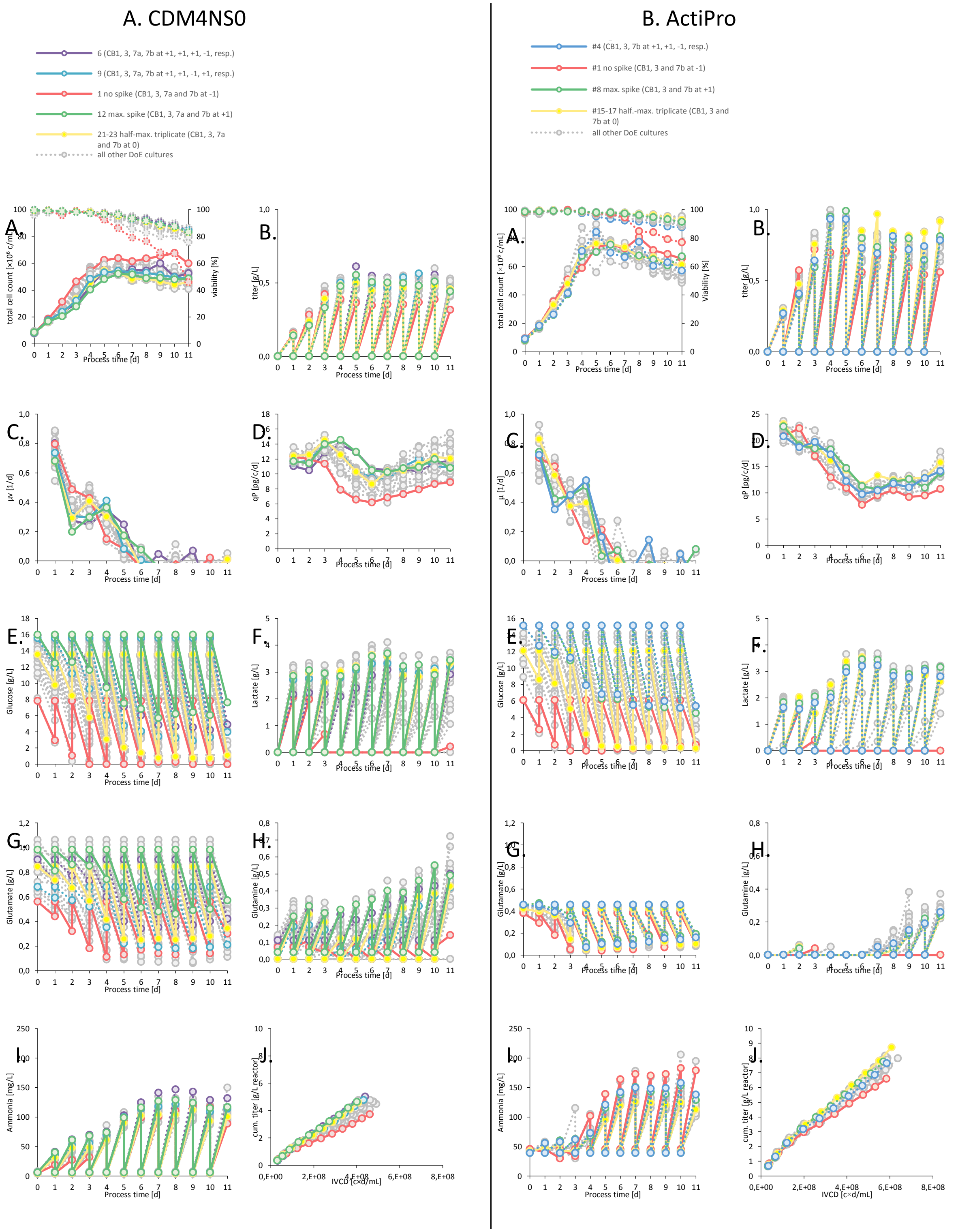

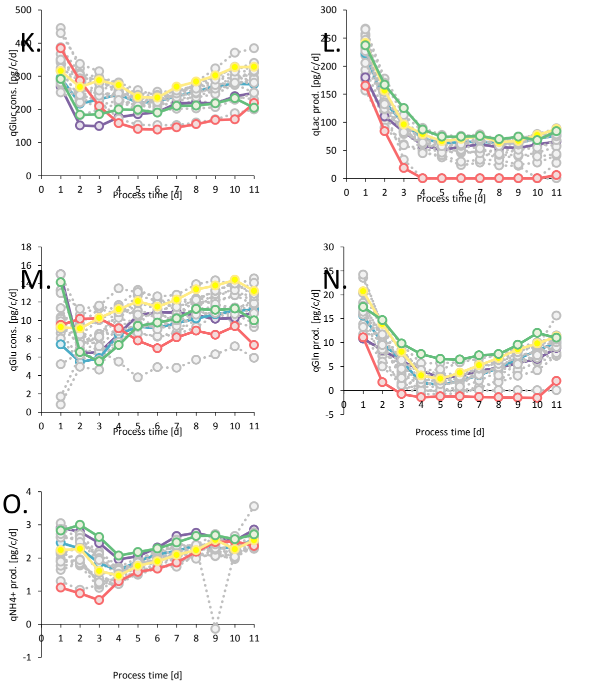
K.
qGluc cons. [pg/c/d]
L.
qLac prod. [pg/c/d]
M.
qGlu cons. [pg/c/d]
N.
qGln prod. [pg/c/d]
O.
qNH4+ prod. [pg/c/d]
Process time [d]

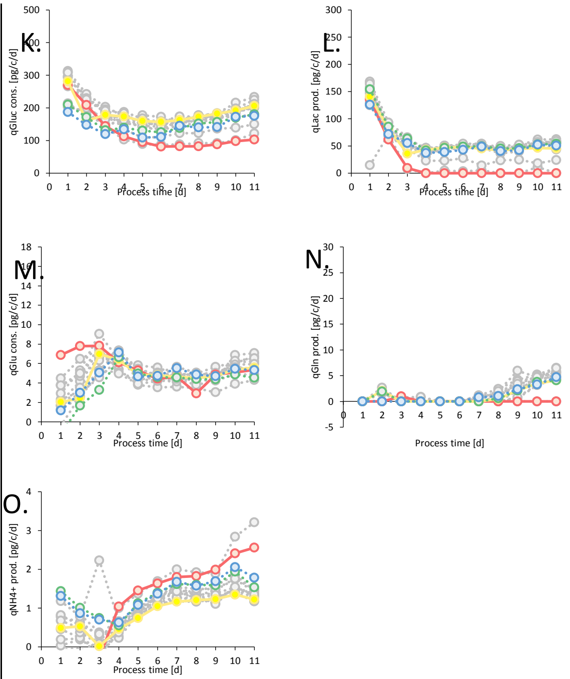
K.
qGluc cons. [pg/c/d]
L.
qLac prod. [pg/c/d]
M.
qGlu cons. [pg/c/d]
N.
qGln prod. [pg/c/d]
O.
qNH4+ prod. [pg/c/d]
Process time [d]

Supplementary Information 7 Summary table of semi-continuous perfusion experiments (DoE2) in CDM4NS0 (A.) or ActiPro (B).

A. CDM4NS0

max and start (d0) values

DoE run #	CB1	CB3	CB7a	CB7b	Design	Start Osm. [mOsm/kg]	IVCD [mio c*d/mL]	Start Gluc [mg/L]	Start Lac [mg/L]	Start Glu [mg/L]	Start Gln [mg/L]	Start NH4+ [mg/L]	Cum. Titer [mg/L]
1	-1	-1	-1	-1	fractional factorial design	300	460.6	7800.0	0.0	560	70	5.0	3737
2	+1	-1	-1	-1	fractional factorial design	351	491.0	13860.0	0.0	610	40	4.0	4485
3	-1	+1	-1	-1	fractional factorial design	312	437.0	12040.0	0.0	560	40	7.0	4867
4	+1	-1	+1	-1	fractional factorial design	383	479.1	15080.0	0.0	930	130	5.0	4670
5	-1	+1	+1	-1	fractional factorial design	345	425.1	13490.0	0.0	910	120	8.0	4452
6	+1	+1	+1	-1	fractional factorial design	384	439.0	15900.0	0.0	900	110	6.0	5022
7	-1	-1	-1	+1	fractional factorial design	306	460.4	8130.0	0.0	650	0	5.0	4095
8	-1	+1	-1	+1	fractional factorial design	318	408.1	11970.0	0.0	640	0	7.0	4775
9	+1	+1	-1	+1	fractional factorial design	364	431.6	15560.0	0.0	680	0	6.0	4776
10	-1	-1	+1	+1	fractional factorial design	344	475.4	10820.0	0.0	1060	140	5.0	4638
11	+1	-1	+1	+1	fractional factorial design	390	437.3	15290.0	0.0	1020	80	5.0	4596
12	+1	+1	+1	+1	fractional factorial design	390	400.3	15980.0	0.0	980	40	6.0	4646
13	-1	0	0	0	face-centered star points	321	409.1	11230.0	0.0	720	70	5.0	4671
14	+1	0	0	0	face-centered star points	375	412.7	15360.0	0.0	840	0	4.0	4702
15	0	-1	0	0	face-centered star points	349	461.6	12630.0	0.0	880	0	4.0	4777
16	0	+1	0	0	face-centered star points	357	406.8	14510.0	0.0	800	40	6.0	4706
17	0	0	-1	0	face-centered star points	337	417.1	13110.0	0.0	670	0	5.0	4678
18	0	0	+1	0	face-centered star points	362	420.3	14170.0	0.0	960	60	5.0	4521
19	0	0	0	-1	face-centered star points	346	434.2	13360.0	0.0	800	40	4.0	4658
20	0	0	0	+1	face-centered star points	352	418.9	13390.0	0.0	850	40	5.0	4738
21	0	0	0	0	center points	351	412.6	13540.0	0.0	843	0	4.7	4755
22	0	0	0	0	center points	351	409.1	13540.0	0.0	843	0	4.7	4505
23	0	0	0	0	center points	351	402.2	13540.0	0.0	843	0	4.7	4476
					Mean ± STDV	350 ± 26	433 ± 27	13230 ± 2178	0 ± 0	807 ± 145	44 ± 46	5 ± 1	4606 ± 261
					CV	7%	6%	16%	n.a.	18%	104%	20%	6%

Exponential Phase (mean of d0-4)

DoE run #	CB1	CB3	CB7a	CB7b	Design	VCD [×10⁶ c/mL]	Viability [%]	Cell diam. [µm]	Cell circularity	Titer [mg/L]	Res. Gluc [mg/L]	Res. Lac [mg/L]	Res. Glu [mg/L]	Res. Gln [mg/L]	Res. NH4+ [mg/L]	µ [1/d]	ΔIVCD ×10⁶ c*d/mL]	qP [pg/c/d]	qGluc [pg/c/d]	qLac [pg/c/d]	qGlu [pg/c/d]	qGln [pg/c/d]	qNH4+ [pg/c/d]
1	-1	-1	-1	-1	fractional factorial design	31.3 ± 13.8	97.9 ± 2.0	13.6 ± 0.6	0.89 ± 0.03	287 ± 133	1327 ± 1497	1580 ± 781	313 ± 130	120 ± 85	26 ± 7	0.57 ± 0.20	24.5 ± 12.3	11.9 ± 0.5	293.7 ± 87.4	89.0 ± 73.4	10.0 ± 0.4	4.0 ± 6.3	0.9 ± 0.2
2	+1	-1	-1	-1	fractional factorial design	28.1 ± 12.8	98.3 ± 0.4	14.6 ± 0.3	0.86 ± 0.01	284 ± 150	7937 ± 2314	2073 ± 76	380 ± 128	153 ± 72	37 ± 6	0.58 ± 0.22	21.8 ± 10.7	12.9 ± 0.5	285.2 ± 48.0	115.2 ± 63.3	10.4 ± 0.8	7.3 ± 6.4	1.7 ± 0.6
3	-1	+1	-1	-1	fractional factorial design	27.6 ± 10.6	98.5 ± 0.6	14.1 ± 0.2	0.90 ± 0.00	267 ± 130	5553 ± 2287	1947 ± 71	393 ± 81	150 ± 53	41 ± 11	0.50 ± 0.21	22.1 ± 9.5	11.9 ± 0.7	302.6 ± 37.1	102.0 ± 50.0	7.6 ± 1.4	6.4 ± 5.1	1.6 ± 0.3
4	+1	-1	+1	-1	fractional factorial design	25.8 ± 9.9	98.3 ± 1.3	14.8 ± 0.2	0.88 ± 0.01	255 ± 133	10710 ± 1336	2363 ± 49	747 ± 49	303 ± 23	51 ± 12	0.50 ± 0.22	20.6 ± 8.7	12.1 ± 1.2	224.0 ± 57.9	130.5 ± 57.8	9.6 ± 3.1	9.5 ± 3.9	2.3 ± 0.4
5	-1	+1	+1	-1	fractional factorial design	24.2 ± 6.2	98.7 ± 0.6	14.3 ± 0.4	0.90 ± 0.01	242 ± 103	8157 ± 1551	2260 ± 17	720 ± 35	270 ± 17	53 ± 16	0.43 ± 0.29	20.2 ± 7.1	11.8 ± 1.0	271.2 ± 42.5	122.8 ± 47.1	10.0 ± 2.9	8.1 ± 2.9	2.2 ± 0.1
6	+1	+1	+1	-1	fractional factorial design	23.3 ± 6.0	98.6 ± 0.6	14.5 ± 0.4	0.90 ± 0.01	226 ± 102	12510 ± 450	2180 ± 35	740 ± 26	263 ± 21	58 ± 15	0.44 ± 0.31	19.4 ± 7.1	11.5 ± 1.2	189.8 ± 68.0	124.4 ± 50.1	9.3 ± 4.9	8.5 ± 2.4	2.7 ± 0.2
7	-1	-1	-1	+1	fractional factorial design	30.3 ± 11.6	98.9 ± 0.5	13.3 ± 0.6	0.92 ± 0.01	300 ± 130	993 ± 1436	2380 ± 1022	457 ± 64	123 ± 85	33 ± 14	0.56 ± 0.29	24.1 ± 11.8	12.7 ± 0.8	331.8 ± 109.6	132.7 ± 104.5	8.5 ± 1.6	7.7 ± 8.3	1.2 ± 0.1
8	-1	+1	-1	+1	fractional factorial design	27.6 ± 11.3	97.9 ± 1.3	13.8 ± 0.1	0.91 ± 0.01	277 ± 131	5013 ± 2347	2693 ± 55	497 ± 59	163 ± 55	41 ± 11	0.52 ± 0.22	21.9 ± 9.7	12.5 ± 0.4	330.9 ± 49.1	143.0 ± 69.2	6.7 ± 1.2	9.4 ± 6.4	1.6 ± 0.3
9	+1	+1	-1	+1	fractional factorial design	24.0 ± 7.1	98.6 ± 0.5	14.5 ± 0.1	0.90 ± 0.00	253 ± 122	10810 ± 1380	2697 ± 61	560 ± 36	187 ± 15	48 ± 11	0.45 ± 0.25	19.8 ± 7.5	12.4 ± 1.4	247.8 ± 41.8	151.9 ± 64.2	6.2 ± 1.0	10.6 ± 4.7	2.2 ± 0.3
10	-1	-1	+1	+1	fractional factorial design	28.3 ± 11.6	98.9 ± 0.4	14.2 ± 0.2	0.90 ± 0.01	306 ± 160	3097 ± 2513	3260 ± 26	793 ± 100	313 ± 55	41 ± 9	0.52 ± 0.18	22.5 ± 10.2	13.3 ± 1.0	360.5 ± 60.6	168.8 ± 80.9	12.4 ± 2.3	9.6 ± 6.2	1.7 ± 0.4
11	+1	-1	+1	+1	fractional factorial design	23.1 ± 7.6	98.5 ± 0.3	14.7 ± 0.2	0.90 ± 0.01	252 ± 117	10463 ± 1333	3247 ± 95	863 ± 55	333 ± 15	52 ± 10	0.45 ± 0.24	18.9 ± 7.1	13.0 ± 1.3	264.7 ± 44.4	189.7 ± 71.7	8.5 ± 1.6	14.8 ± 5.4	2.6 ± 0.5
12	+1	+1	+1	+1	fractional factorial design	21.3 ± 5.4	98.6 ± 0.5	14.7 ± 0.3	0.90 ± 0.01	227 ± 96	12237 ± 539	2953 ± 105	840 ± 26	277 ± 31	56 ± 15	0.39 ± 0.26	18.0 ± 5.8	12.4 ± 1.4	219.9 ± 62.0	176.2 ± 56.3	8.7 ± 4.7	14.0 ± 3.9	2.8 ± 0.2
13	-1	0	0	0	face-centered star points	26.4 ± 7.9	98.7 ± 0.5	13.9 ± 0.2	0.91 ± 0.00	273 ± 128	4880 ± 2095	2687 ± 172	633 ± 68	243 ± 47	40 ± 11	0.50 ± 0.34	21.5 ± 9.1	12.5 ± 0.8	305.3 ± 40.1	146.3 ± 77.8	3.5 ± 2.3	10.0 ± 6.8	1.7 ± 0.3
14	+1	0	0	0	face-centered star points	23.6 ± 8.1	98.6 ± 0.4	14.3 ± 0.2	0.91 ± 0.00	245 ± 124	10930 ± 1262	2657 ± 133	673 ± 47	253 ± 21	49 ± 11	0.47 ± 0.21	19.3 ± 7.8	12.4 ± 1.4	239.7 ± 39.9	157.0 ± 71.6	9.1 ± 1.9	15.2 ± 7.6	2.5 ± 0.5
15	0	-1	0	0	face-centered star points	27.3 ± 12.0	99.0 ± 0.3	13.8 ± 0.1	0.92 ± 0.00	280 ± 155	6087 ± 2465	2963 ± 67	630 ± 111	240 ± 61	39 ± 8	0.55 ± 0.20	21.3 ± 10.2	12.8 ± 1.0	319.3 ± 42.5	164.3 ± 82.1	11.9 ± 0.9	14.2 ± 9.5	1.8 ± 0.5
16	0	+1	0	0	face-centered star points	23.1 ± 8.3	98.4 ± 0.5	14.1 ± 0.2	0.91 ± 0.00	241 ± 113	9957 ± 1493	2630 ± 89	690 ± 46	233 ± 21	50 ± 12	0.45 ± 0.16	18.8 ± 7.5	12.6 ± 0.9	247.9 ± 23.9	157.9 ± 68.9	5.8 ± 0.7	11.9 ± 6.0	2.4 ± 0.4
17	0	0	-1	0	face-centered star points	26.6 ± 11.5	97.9 ± 0.3	13.7 ± 0.1	0.91 ± 0.01	276 ± 149	6710 ± 2432	2730 ± 30	473 ± 103	160 ± 70	39 ± 9	0.51 ± 0.15	21.1 ± 9.4	12.7 ± 1.3	311.5 ± 32.6	149.8 ± 70.9	9.2 ± 0.8	9.8 ± 7.3	1.7 ± 0.4
18	0	0	+1	0	face-centered star points	24.3 ± 8.9	98.6 ± 0.4	14.2 ± 0.2	0.91 ± 0.00	242 ± 116	9520 ± 1912	2917 ± 47	847 ± 90	300 ± 10	50 ± 11	0.48 ± 0.20	19.6 ± 8.1	12.1 ± 0.9	237.9 ± 15.1	167.9 ± 72.7	5.0 ± 2.9	13.9 ± 6.1	2.4 ± 0.4
19	0	0	0	-1	face-centered star points	26.3 ± 11.9	98.9 ± 0.3	13.8 ± 0.1	0.92 ± 0.00	275 ± 142	7987 ± 2322	2540 ± 40	597 ± 97	227 ± 42	44 ± 11	0.49 ± 0.07	20.9 ± 9.4	12.9 ± 1.0	260.7 ± 19.0	140.0 ± 62.1	9.7 ± 0.4	10.7 ± 6.0	2.0 ± 0.4
20	0	0	0	+1	face-centered star points	23.6 ± 8.5	98.9 ± 0.4	14.0 ± 0.0	0.92 ± 0.00	268 ± 135	7697 ± 2008	3130 ± 98	693 ± 57	240 ± 26	42 ± 10	0.46 ± 0.21	19.2 ± 7.5	13.5 ± 1.8	300.4 ± 20.7	182.6 ± 77.1	8.3 ± 0.9	11.9 ± 5.6	2.0 ± 0.4
21	0	0	0	0	center points	24.9 ± 10.8	98.6 ± 0.4	13.9 ± 0.0	0.92 ± 0.01	265 ± 134	7820 ± 2327	2887 ± 51	657 ± 81	237 ± 40	43 ± 11	0.51 ± 0.19	19.7 ± 8.6	13.2 ± 1.0	294.5 ± 21.1	167.1 ± 72.2	9.5 ± 0.3	14.3 ± 7.9	2.1 ± 0.4
22	0	0	0	0	center points	23.4 ± 7.1	98.8 ± 0.6	14.0 ± 0.0	0.92 ± 0.00	253 ± 109	8133 ± 1750	2890 ± 110	667 ± 60	247 ± 23	43 ± 10	0.42 ± 0.19	19.5 ± 7.4	12.8 ± 0.7	281.9 ± 20.3	165.5 ± 69.5	9.1 ± 0.4	14.3 ± 6.8	2.1 ± 0.4
23	0	0	0	0	center points	23.1 ± 7.8	98.7 ± 0.4	13.9 ± 0.0	0.92 ± 0.00	261 ± 129	8033 ± 2121	2683 ± 474	647 ± 114	243 ± 25	41 ± 9	0.45 ± 0.27	18.8 ± 6.8	13.5 ± 2.0	294.6 ± 39.4	161.4 ± 77.9	10.0 ± 2.3	14.0 ± 4.4	2.0 ± 0.3
					Mean ± STDV	25.5 ± 2.5	98.6 ± 0.3	14.1 ± 0.4	0.90 ± 0.01	263 ± 21	7677 ± 3160	2624 ± 418	631 ± 153	230 ± 61	44 ± 8	0.49 ± 0.05	20.6 ± 1.7	12.6 ± 0.6	278.9 ± 41.3	148.1 ± 25.6	8.7 ± 2.1	10.9 ± 3.1	2.0 ± 0.5
					CV	10%	0%	3%	2%	8%	41%	16%	24%	26%	17%	10%	8%	4%	15%	17%	24%	28%	23%

Stationary phase (mean of d5-11)

DoE run #	CB1	CB3	CB7a	CB7b	Design	VCD [×10⁶ c/mL]	Viability [%]	Cell diam. [µm]	Cell circularity	Titer [mg/L]	Res. Gluc [mg/L]	Res. Lac [mg/L]	Res. Glu [mg/L]	Res. Gln [mg/L]	Res. NH4+ [mg/L]	µ [1/d]	ΔIVCD ×10⁶ c*d/mL]	qP [pg/c/d]	qGluc [pg/c/d]	qLac [pg/c/d]	qGlu [pg/c/d]	qGln [pg/c/d]	qNH4+ [pg/c/d]
1	-1	-1	-1	-1	fractional factorial design	46.6 ± 9.8	73 ± 15	12.9 ± 0.5	0.9 ± 0.0	355.5 ± 21.9	105.7 ± 133.9	31.4 ± 83.2	168.6 ± 60.4	20.0 ± 52.9	104.1 ± 12.1	-0.1 ± 0.2	48.3 ± 7.0	7.5 ± 1.0	162.8 ± 27.9	0.9 ± 2.3	8.1 ± 0.9	-0.9 ± 1.3	2.1 ± 0.4
2	+1	-1	-1	-1	fractional factorial design	53.1 ± 6.9	92 ± 5	15.3 ± 0.2	0.9 ± 0.0	448.4 ± 20.8	877.1 ± 367.4	2765.7 ± 90.7	98.6 ± 18.6	167.1 ± 149.7	109.1 ± 6.3	0.0 ± 0.1	53.9 ± 6.2	8.4 ± 1.2	244.3 ± 32.7	51.9 ± 5.5	9.6 ± 1.3	2.7 ± 3.2	2.0 ± 0.3
3	-1	+1	-1	-1	fractional factorial design	45.9 ± 6.2	88 ± 6	14.0 ± 0.4	0.9 ± 0.0	517.4 ± 44.1	0.0 ± 0.0	1105.7 ± 567.8	90.0 ± 33.2	140.0 ± 106.3	97.3 ± 8.4	0.0 ± 0.1	46.8 ± 5.0	11.2 ± 2.0	260.1 ± 30.1	23.3 ± 11.4	10.1 ± 0.6	2.4 ± 2.7	2.0 ± 0.3
4	+1	-1	+1	-1	fractional factorial design	53.4 ± 4.3	93 ± 4	15.7 ± 0.4	0.9 ± 0.0	481.4 ± 43.0	3160.0 ± 726.8	3131.4 ± 158.9	305.7 ± 42.8	417.1 ± 149.0	122.3 ± 11.5	0.0 ± 0.1	53.6 ± 3.0	9.0 ± 0.9	222.6 ± 9.7	58.6 ± 4.0	11.7 ± 1.0	5.4 ± 3.1	2.2 ± 0.2
5	-1	+1	+1	-1	fractional factorial design	46.3 ± 4.2	89 ± 5	15.6 ± 0.4	0.9 ± 0.0	464.3 ± 43.5	1082.9 ± 570.2	2754.3 ± 178.4	430.0 ± 52.0	338.6 ± 95.8	112.1 ± 8.4	0.0 ± 0.1	46.8 ± 2.7	9.9 ± 0.8	265.9 ± 17.0	59.1 ± 5.0	10.2 ± 0.6	4.7 ± 2.2	2.2 ± 0.2
6	+1	+1	+1	-1	fractional factorial design	49.6 ± 4.1	92 ± 4	15.9 ± 0.5	0.9 ± 0.0	550.4 ± 29.2	5172.9 ± 1022.9	2838.6 ± 210.3	377.1 ± 29.8	358.6 ± 94.6	131.4 ± 14.9	0.0 ± 0.1	49.4 ± 2.9	11.2 ± 0.9	217.8 ± 23.1	57.6 ± 5.1	10.6 ± 0.3	5.1 ± 2.2	2.5 ± 0.3
7	-1	-1	-1	+1	fractional factorial design	47.6 ± 5.8	84 ± 6	13.3 ± 0.5	0.9 ± 0.0	402.8 ± 18.9	28.6 ± 75.6	0.0 ± 0.0	385.7 ± 38.7	0.0 ± 0.0	118.7 ± 16.0	0.0 ± 0.1	48.7 ± 5.0	8.4 ± 1.3	168.1 ± 18.9	0.0 ± 0.0	5.5 ± 1.1	0.0 ± 0.0	2.4 ± 0.6
8	-1	+1	-1	+1	fractional factorial design	42.0 ± 6.5	87 ± 6	14.2 ± 0.4	0.9 ± 0.0	497.1 ± 41.6	0.0 ± 0.0	1984.3 ± 368.4	221.4 ± 16.8	161.4 ± 123.9	93.0 ± 4.7	0.0 ± 0.1	42.8 ± 5.6	11.9 ± 2.3	283.8 ± 38.3	46.4 ± 7.0	9.9 ± 1.4	4.1 ± 3.5	2.0 ± 0.3
9	+1	+1	-1	+1	fractional factorial design	47.2 ± 4.1	91 ± 4	15.2 ± 0.4	0.9 ± 0.0	502.0 ± 37.6	3644.3 ± 643.1	3157.1 ± 130.9	197.1 ± 16.0	235.7 ± 137.0	112.9 ± 7.0	0.0 ± 0.1	47.6 ± 3.4	10.6 ± 1.0	251.5 ± 24.4	66.6 ± 5.1	10.2 ± 0.8	5.1 ± 3.3	2.3 ± 0.2
10	-1	-1	+1	+1	fractional factorial design	50.7 ± 5.2	89 ± 6	14.0 ± 0.3	0.9 ± 0.0	457.7 ± 37.0	0.0 ± 0.0	1694.3 ± 326.4	448.6 ± 31.3	371.4 ± 117.4	112.3 ± 7.1	0.0 ± 0.1	51.5 ± 4.5	9.0 ± 1.2	211.6 ± 20.0	33.0 ± 6.2	11.9 ± 0.8	4.7 ± 2.7	2.1 ± 0.2
11	+1	-1	+1	+1	fractional factorial design	49.2 ± 3.7	92 ± 5	15.5 ± 0.5	0.9 ± 0.0	476.4 ± 38.0	3481.4 ± 622.5	3731.4 ± 234.0	410.0 ± 38.3	467.1 ± 78.3	109.4 ± 11.3	0.0 ± 0.1	49.1 ± 3.2	9.7 ± 1.0	241.4 ± 18.4	76.1 ± 3.8	12.4 ± 0.6	8.0 ± 2.0	2.1 ± 0.2
12	+1	+1	+1	+1	fractional factorial design	44.9 ± 3.5	91 ± 5	15.7 ± 0.4	0.9 ± 0.0	498.3 ± 33.1	6610.0 ± 734.6	3322.9 ± 273.0	512.9 ± 45.4	422.9 ± 81.2	118.1 ± 10.1	0.0 ± 0.1	44.8 ± 3.1	11.2 ± 1.0	209.5 ± 14.2	74.3 ± 5.1	10.4 ± 0.8	8.6 ± 2.2	2.5 ± 0.2
13	-1	0	0	0	face-centered star points	42.4 ± 5.4	88 ± 6	14.2 ± 0.4	0.9 ± 0.0	479.4 ± 28.3	0.0 ± 0.0	1917.1 ± 362.3	288.6 ± 41.0	275.7 ± 112.5	99.1 ± 6.0	0.0 ± 0.1	43.4 ± 5.0	11.2 ± 1.9	262.2 ± 31.3	44.0 ± 3.7	10.0 ± 0.7	5.1 ± 3.2	2.2 ± 0.3
14	+1	0	0	0	face-centered star points	44.6 ± 5.1	92 ± 4	15.3 ± 0.5	0.9 ± 0.0	493.5 ± 23.3	3920.0 ± 534.6	3104.3 ± 222.3	301.4 ± 44.5	300.0 ± 106.3	109.7 ± 7.9	0.0 ± 0.1	45.1 ± 4.4	11.0 ± 0.9	255.8 ± 30.2	69.3 ± 7.8	11.9 ± 0.4	6.9 ± 3.1	2.4 ± 0.3
15	0	-1	0	0	face-centered star points	49.2 ± 5.4	91 ± 5	14.4 ± 0.4	0.9 ± 0.0	494.0 ± 19.0	0.0 ± 0.0	2787.1 ± 431.6	201.4 ± 33.4	251.4 ± 147.1	107.4 ± 9.1	0.0 ± 0.1	50.1 ± 4.6	9.9 ± 1.1	253.9 ± 24.0	55.7 ± 7.8	13.6 ± 0.8	5.3 ± 3.5	2.1 ± 0.3
16	0	+1	0	0	face-centered star points	44.0 ± 6.2	90 ± 5	15.0 ± 0.4	0.9 ± 0.0	497.5 ± 20.1	2791.4 ± 542.9	2952.9 ± 235.3	334.3 ± 33.1	297.1 ± 85.2	102.8 ± 6.8	0.0 ± 0.1	44.6 ± 5.1	11.3 ± 1.3	266.1 ± 36.9	66.8 ± 8.6	10.5 ± 0.9	6.0 ± 2.6	1.8 ± 0.9
17	0	0	-1	0	face-centered star points	42.8 ± 8.4	89 ± 5	14.7 ± 0.4	0.9 ± 0.0	479.4 ± 89.1	495.7 ± 734.3	2997.1 ± 386.1	171.4 ± 59.6	168.6 ± 114.4	97.3 ± 12.4	-0.1 ± 0.1	44.1 ± 7.9	11.3 ± 3.4	295.7 ± 64.6	69.2 ± 11.2	11.6 ± 2.0	4.3 ± 3.3	2.1 ± 0.4
18	0	0	+1	0	face-centered star points	45.4 ± 4.2	90 ± 5	15.3 ± 0.4	0.9 ± 0.0	468.7 ± 57.8	2597.1 ± 721.0	3295.7 ± 327.6	455.7 ± 26.4	428.6 ± 140.3	110.1 ± 10.2	0.0 ± 0.1	45.9 ± 3.6	10.3 ± 1.7	254.4 ± 32.5	71.8 ± 4.3	11.1 ± 1.1	8.2 ± 3.6	2.3 ± 0.3
19	0	0	0	-1	face-centered star points	46.0 ± 6.8	90 ± 5	15.0 ± 0.4	0.9 ± 0.0	483.1 ± 28.9	925.7 ± 564.0	3027.1 ± 328.4	248.6 ± 29.7	302.9 ± 121.5	106.4 ± 7.3	0.0 ± 0.1	46.7 ± 6.3	10.5 ± 1.9	271.5 ± 46.4	65.4 ± 7.9	12.0 ± 1.5	6.0 ± 3.3	2.2 ± 0.3
20	0	0	0	+1	face-centered star points	45.7 ± 5.4	90 ± 5	15.0 ± 0.4	0.9 ± 0.0	492.5 ± 22.6	847.1 ± 390.5	3465.7 ± 286.6	281.4 ± 26.1	318.6 ± 121.0	101.9 ± 8.7	0.0 ± 0.1	46.1 ± 4.8	10.8 ± 1.3	274.9 ± 31.8	75.6 ± 7.0	12.4 ± 1.0	6.3 ± 3.3	2.1 ± 0.2
21	0	0	0	0	center points	44.2 ± 6.6	90 ± 5	14.9 ± 0.5	0.9 ± 0.0	499.0 ± 28.9	795.7 ± 607.1	3202.9 ± 302.9	261.4 ± 28.5	282.9 ± 108.3	102.4 ± 7.6	0.0 ± 0.1	44.7 ± 5.6	11.3 ± 1.7	290.1 ± 47.3	72.2 ± 8.2	13.2 ± 1.5	6.7 ± 3.3	2.2 ± 0.4
22	0	0	0	0	center points	44.2 ± 6.0	90 ± 5	14.9 ± 0.5	0.9 ± 0.0	464.3 ± 21.0	1200.0 ± 511.4	3211.4 ± 303.2	265.7 ± 38.2	292.9 ± 120.8	102.1 ± 8.0	0.0 ± 0.1	44.7 ± 5.3	10.5 ± 1.3	280.1 ± 39.2	72.5 ± 9.3	13.0 ± 1.0	6.9 ± 3.5	2.2 ± 0.3
23	0	0	0	0	center points	43.8 ± 5.3	90 ± 5	14.9 ± 0.5	0.9 ± 0.0	458.8 ± 21.3	1291.4 ± 379.8	3222.9 ± 375.8	287.1 ± 39.9	294.3 ± 106.0	98.4 ± 7.1	0.0 ± 0.1	44.1 ± 4.4	10.5 ± 0.9	280.2 ± 30.9	73.3 ± 7.7	12.7 ± 0.8	6.9 ± 3.0	2.1 ± 0.3
					Mean ± STDV	46.5 ± 3.1	89 ± 4	14.8 ± 0.8	0.9 ± 0.0	476.6 ± 38.4	1696.8 ± 1871.2	2595.7 ± 1019.6	293.2 ± 113.8	274.5 ± 121.4	107.8 ± 9.1	0.0 ± 0.0	47.1 ± 3.1	10.3 ± 1.1	248.9 ± 35.6	55.8 ± 22.3	11.0 ± 1.8	5.2 ± 2.4	2.2 ± 0.2
					CV	7%	4%	%	2%	8%	110%	39%	39%	44%	8%	-78%	7%	11%	14%	40%	16%	46%	8%

B. ActiPro

max and start (d0) values

DoE run #	CB1	CB3	CB7b	Design	Start Osm [mOsm/kg]	Res. Osm [mOsm/kg]	IVCD [×106 c×d/mL]	Start Gluc [mg/L]	Start Lac [mOsm/kg]	Start Glu [mg/L]	Start Gln [mg/L]	Start NH4+ [mg/L]	Cum. Titer OctetRed [mg/L]
1	-1	-1	-1	fractional factorial design	337	300 ± 17	584	6100	0.0	380	0.0	45.5	6605
2	+1	-1	-1		399	371 ± 21	575	13705	0.0	445	0.0	42.5	7125
3	-1	+1	-1		348	310 ± 23	559	11010	0.0	400	0.0	42.5	7843
4	+1	+1	-1		397	371 ± 23	585	15135	0.0	455	0.0	39	7653
5	-1	-1	+1		337	304 ± 17	574	6100	0.0	380	0.0	45.5	6742
6	+1	-1	+1		399	376 ± 18	541	13705	0.0	445	0.0	42.5	7344
7	-1	+1	+1		348	314 ± 27	589	11010	0.0	400	0.0	42.5	8001
8	+1	+1	+1		397	374 ± 20	570	15135	0.0	455	0.0	39	7767
9	-1	0	0		347	303 ± 25	637	8925	0.0	380	0.0	44	7996
10	+1	0	0		398	371 ± 18	587	14195	0.0	445	0.0	41	7879
11	0	-1	0		371	337 ± 22	548	10480	0.0	405	0.0	44.5	7570
12	0	+1	0		373	344 ± 21	578	13130	0.0	430	0.0	41	8165
13	0	0	-1		375	328 ± 18	595	12095	0.0	420	0.0	42	7479
14	0	0	+1		375	342 ± 22	587	12095	0.0	420	0.0	42	7875
15	0	0	0	center points	375	338 ± 21	633	12095	0.0	420	0.0	42	8753
16	0	0	0		375	334 ± 22	614	12095	0.0	420	0.0	42	8970
17	0	0	0		375	339 ± 21	583	12095	0.0	420	0.0	42	8473
mean:					372 ± 22	338 ± 27	585 ± 26	11712 ± 2664	0 ± 0	419 ± 25	0 ± 0	42 ± 2	7779 ± 629
CV:					6%	8%	4%	23%	n.a.	6%	n.a.	4%	8%

Exponential phase (d0-4)

DoE run #	CB1	CB3	CB7b	Design	VCD [×10⁶ c/mL]	Viability [%]	Cell diameter [µm[	Circularity	Titer OctetRed [mg/L]	Res. Gluc [mg/L]	Res. Lac [mg/L]	Res. Glu [mg/L]	Res. Gln [mg/L]
1	-1	-1	-1	fractional factorial design	34.6 ± 16.1	99.0 ± 0.5	14.0 ± 0.7	0.91 ± 0.01	521 ± 226	1097 ± 1319	1257 ± 748	173 ± 120	13 ± 23
2	+1	-1	-1		29.5 ± 12.8	99.0 ± 0.3	14.7 ± 0.3	0.90 ± 0.01	426 ± 185	9413 ± 1805	1757 ± 191	323 ± 112	0 ± 0
3	-1	+1	-1		33.4 ± 17.3	99.2 ± 0.2	14.4 ± 0.4	0.89 ± 0.01	509 ± 256	5577 ± 1925	1697 ± 100	260 ± 108	0 ± 0
4	+1	+1	-1		28.2 ± 11.3	98.7 ± 0.3	14.9 ± 0.3	0.90 ± 0.01	439 ± 188	11950 ± 741	1670 ± 125	373 ± 76	0 ± 0
5	-1	-1	+1		35.3 ± 16.5	99.3 ± 0.2	13.6 ± 0.7	0.92 ± 0.01	541 ± 253	820 ± 1131	1403 ± 935	197 ± 131	10 ± 17
6	+1	-1	+1		27.6 ± 12.2	98.3 ± 1.3	14.8 ± 0.3	0.89 ± 0.01	412 ± 164	9017 ± 1515	2043 ± 129	373 ± 86	0 ± 0
7	-1	+1	+1		35.5 ± 17.7	99.0 ± 0.6	14.4 ± 0.5	0.90 ± 0.01	533 ± 274	5060 ± 2187	1370 ± 1030	287 ± 104	17 ± 29
8	+1	+1	+1		27.4 ± 11.6	98.6 ± 0.2	14.9 ± 0.4	0.89 ± 0.00	420 ± 164	11703 ± 880	1887 ± 144	413 ± 60	13 ± 23
9	-1	0	0		36.9 ± 19.2	98.9 ± 0.5	14.1 ± 0.6	0.90 ± 0.01	549 ± 242	2757 ± 2227	1907 ± 99	207 ± 150	13 ± 23
10	+1	0	0		28.4 ± 12.0	98.2 ± 1.1	14.8 ± 0.4	0.89 ± 0.01	427 ± 191	10417 ± 1202	1877 ± 159	363 ± 83	13 ± 23
11	0	-1	0		32.1 ± 14.9	98.8 ± 0.1	14.4 ± 0.2	0.89 ± 0.01	547 ± 284	4733 ± 1980	1960 ± 104	237 ± 159	10 ± 17
12	0	+1	0		32.3 ± 15.6	98.9 ± 0.4	14.7 ± 0.3	0.90 ± 0.01	491 ± 237	8883 ± 1634	1837 ± 129	340 ± 82	17 ± 29
13	0	0	-1		33.3 ± 16.3	99.2 ± 0.0	14.4 ± 0.4	0.90 ± 0.00	490 ± 225	6285 ± 1365	1880 ± 184	215 ± 120	20 ± 28
14	0	0	+1		29.6 ± 13.0	99.0 ± 0.1	14.5 ± 0.4	0.90 ± 0.01	493 ± 228	6773 ± 1717	1990 ± 132	310 ± 108	20 ± 35
15	0	0	0	center points	33.3 ± 15.4	99.3 ± 0.1	14.5 ± 0.2	0.90 ± 0.01	509 ± 245	7157 ± 1888	1360 ± 1009	293 ± 133	17 ± 29
16	0	0	0		33.7 ± 15.0	99.1 ± 0.3	14.4 ± 0.3	0.90 ± 0.01	498 ± 225	6970 ± 2687	1835 ± 276	275 ± 191	0 ± 0
17	0	0	0		31.5 ± 13.6	99.1 ± 0.2	14.3 ± 0.2	0.90 ± 0.00	515 ± 232	5070 ± n.a.*	2010 ± n.a.*	150 ± n.a.*	0 ± n.a.*
mean:					31.9 ± 3.0	98.9 ± 0.3	14.4 ± 0.3	0.90 ± 0.01	489 ± 225	6687 ± 3320	1749 ± 252	282 ± 77	10 ± 8
CV:					9%	0%	2%	1%	46%	50%	14%	27%	81%

DoE run #	Res. NH4+ [mg/L]	µ [1/d]	ΔIVCD [×10⁶ c/mL]	qP [pg/c/d]	qGluc [pg/c/d]	qLac [pg/c/d]	qGlu [pg/c/d]	qGln [pg/c/d]	qNH4+ [pg/c/d]
1	35 ± 6	0.57 ± 0.18	27.0 ± 14.6	20.0 ± 2.7	207.8 ± 62.5	69.0 ± 63.3	7.5 ± 0.6	0.3 ± 0.5	-0.4 ± 0.2
2	54 ± 3	0.50 ± 0.22	23.8 ± 12.0	18.4 ± 1.4	186.5 ± 17.4	86.4 ± 38.6	4.3 ± 2.4	0.0 ± 0.0	0.6 ± 0.3
3	43 ± 4	0.57 ± 0.06	25.6 ± 13.5	20.0 ± 0.7	226.4 ± 40.7	79.7 ± 40.9	5.0 ± 1.4	0.0 ± 0.0	0.1 ± 0.1
4	59 ± 3	0.51 ± 0.19	22.5 ± 9.8	19.7 ± 1.0	151.6 ± 33.7	84.4 ± 36.7	3.1 ± 2.0	0.0 ± 0.0	1.0 ± 0.3
5	41 ± 7	0.57 ± 0.18	27.6 ± 14.9	20.2 ± 1.7	219.0 ± 77.7	76.5 ± 72.2	6.1 ± 1.5	0.2 ± 0.4	-0.2 ± 0.3
6	58 ± 3	0.51 ± 0.11	22.0 ± 10.7	19.5 ± 2.9	226.4 ± 40.6	109.2 ± 53.0	2.4 ± 2.7	0.0 ± 0.0	0.8 ± 0.5
7	45 ± 4	0.60 ± 0.15	27.4 ± 15.3	19.9 ± 1.2	236.3 ± 52.9	44.7 ± 28.6	3.6 ± 1.5	0.7 ± 1.1	0.1 ± 0.1
8	60 ± 4	0.54 ± 0.18	21.6 ± 10.2	20.0 ± 2.3	170.9 ± 39.2	101.1 ± 47.4	1.2 ± 2.3	0.6 ± 1.1	1.1 ± 0.3
9	39 ± 8	0.63 ± 0.15	28.0 ± 15.7	20.6 ± 2.7	241.6 ± 60.1	85.1 ± 48.6	5.5 ± 2.0	0.5 ± 0.9	-0.1 ± 0.1
10	76 ± 34	0.57 ± 0.25	22.2 ± 10.7	19.6 ± 1.8	180.4 ± 32.4	98.4 ± 46.5	3.0 ± 2.1	0.6 ± 1.1	1.4 ± 0.7
11	45 ± 8	0.63 ± 0.26	24.6 ± 13.0	22.3 ± 0.5	252.9 ± 55.9	97.5 ± 53.5	5.7 ± 3.1	0.3 ± 0.5	0.2 ± 0.3
12	53 ± 2	0.57 ± 0.15	25.1 ± 13.7	20.2 ± 2.0	180.3 ± 31.2	90.2 ± 50.7	3.0 ± 1.5	0.7 ± 1.2	0.6 ± 0.2
13	48 ± 4	0.58 ± 0.17	25.9 ± 14.4	19.8 ± 2.4	182.4 ± 24.4	60.5 ± 16.4	6.0 ± 1.5	0.8 ± 1.2	0.2 ± 0.2
14	49 ± 4	0.52 ± 0.22	23.7 ± 12.3	21.5 ± 2.0	244.4 ± 58.5	102.0 ± 55.1	3.8 ± 2.3	0.9 ± 1.5	0.4 ± 0.3
15	49 ± 7	0.64 ± 0.28	25.6 ± 13.8	20.6 ± 2.7	214.9 ± 79.4	79.5 ± 73.1	4.2 ± 2.4	0.7 ± 1.2	0.4 ± 0.3
16	45 ± 2	0.60 ± 0.25	26.4 ± 14.3	19.7 ± 2.6	213.6 ± 60.2	89.9 ± 57.2	3.8 ± 4.3	0.0 ± 0.0	0.2 ± 0.2
17	43 ± n.a.*	0.55 ± 0.18	25.0 ± 12.6	21.1 ± 2	184.9 ± n.a.*	52.9 ± n.a.*	7.1 ± n.a.*	0.0 ± n.a.*	0.0 ± n.a.*
mean:	49 ± 10	0.57 ± 0.04	24.9 ± 2.0	20.2 ± 0.8	207.1 ± 29.4	82.8 ± 17.8	4.4 ± 1.7	0.4 ± 0.3	0.4 ± 0.5
CV:	20%	8%	8%	4%	14%	22%	38%	89%	133%

Stationary phase (d5-11)

DoE run #	CB1	CB3	CB7b	Design	VCD [×10⁶ c/mL]	Viability [%]	Cell diameter [µm[	Circularity	Titer OctetRed [mg/L]	Res. Gluc [mg/L]	Res. Lac [mg/L]	Res. Glu [mg/L]	Res. Gln [mg/L]
1	-1	-1	-1	fractional factorial design	63.7 ± 8.9	87.6 ± 8.5	13.1 ± 0.5	0.92 ± 0.01	620 ± 76	371 ± 239	0 ± 0	84 ± 47	0 ± 0
2	+1	-1	-1		62.8 ± 10.1	94.5 ± 2.5	15.3 ± 0.8	0.88 ± 0.03	710 ± 108	2963 ± 246	3114 ± 223	107 ± 26	63 ± 96
3	-1	+1	-1		59.2 ± 8.8	95.8 ± 1.4	14.8 ± 0.7	0.90 ± 0.03	774 ± 112	306 ± 53	2779 ± 447	64 ± 11	121 ± 108
4	+1	+1	-1		65.2 ± 10.6	95.4 ± 2.4	15.3 ± 0.9	0.88 ± 0.03	772 ± 81	5877 ± 686	2971 ± 191	123 ± 28	103 ± 100
5	-1	-1	+1		60.0 ± 9.6	91.1 ± 2.7	13.4 ± 0.5	0.92 ± 0.01	635 ± 33	260 ± 135	217 ± 235	156 ± 24	0 ± 0
6	+1	-1	+1		59.4 ± 11.5	92.9 ± 3.0	15.3 ± 0.7	0.89 ± 0.02	747 ± 88	2727 ± 255	3219 ± 341	130 ± 39	87 ± 117
7	-1	+1	+1		59.8 ± 9.3	95.1 ± 1.9	15.0 ± 0.7	0.90 ± 0.02	780 ± 81	116 ± 145	2960 ± 254	104 ± 25	153 ± 131
8	+1	+1	+1		64.4 ± 5.6	95.3 ± 2.5	15.3 ± 0.7	0.89 ± 0.01	794 ± 103	5480 ± 631	3160 ± 214	157 ± 27	90 ± 106
9	-1	0	0		68.0 ± 11.1	96.3 ± 1.2	13.8 ± 0.6	0.92 ± 0.01	771 ± 88	191 ± 139	1550 ± 404	81 ± 25	174 ± 170
10	+1	0	0		66.5 ± 8.8	95.6 ± 2.4	15.1 ± 0.7	0.90 ± 0.01	800 ± 109	3800 ± 274	3261 ± 253	116 ± 23	71 ± 107
11	0	-1	0		58.2 ± 8.3	94.0 ± 2.9	14.2 ± 0.8	0.92 ± 0.01	710 ± 132	139 ± 134	3000 ± 337	83 ± 21	120 ± 123
12	0	+1	0		62.5 ± 10.2	95.1 ± 2.4	15.0 ± 0.6	0.89 ± 0.01	803 ± 76	2703 ± 254	3176 ± 224	116 ± 24	134 ± 124
13	0	0	-1		64.5 ± 6.5	96.6 ± 1.3	15.2 ± 1.0	0.90 ± 0.02	732 ± 62	1177 ± 266	3214 ± 220	80 ± 10	120 ± 110
14	0	0	+1		64.4 ± 10.6	96.0 ± 1.8	15.3 ± 1.0	0.89 ± 0.01	777 ± 77	1046 ± 166	3390 ± 266	111 ± 20	111 ± 124
15	0	0	0	center points	69.4 ± 11.1	95.8 ± 2.0	15.0 ± 0.8	0.91 ± 0.01	905 ± 126	259 ± 210	2963 ± 326	93 ± 27	99 ± 120
16	0	0	0		67.5 ± 3.1	97.2 ± 0.5	14.6 ± 0.6	0.90 ± 0.01	924 ± 113	334 ± 226	3156 ± 480	90 ± 24	26 ± 58
17	0	0	0		64.3 ± 5.7	96.1 ± 1.7	14.7 ± 0.6	0.90 ± 0.01	855 ± 81	621 ± 296	3131 ± 387	91 ± 20	81 ± 88
mean:					63.5 ± 3.3	94.7 ± 2.3	14.7 ± 0.7	0.90 ± 0.01	771 ± 91	1669 ± 1914	2662 ± 1042	105 ± 26	91 ± 49
CV:					5%	2%	5%	1%	12%	115%	39%	25%	53%

DoE run #	Res. NH4+ [mg/L]	µ [1/d]	ΔIVCD [×10⁶ c/mL]	qP [pg/c/d]	qGluc [pg/c/d]	qLac [pg/c/d]	qGlu [pg/c/d]	qGln [pg/c/d]	qNH4+ [pg/c/d]
1	169 ± 14	-0.02 ± 0.12	64.1 ± 7.3	9.7 ± 1.1	90.1 ± 8.6	0.0 ± 0.0	4.7 ± 0.8	0.0 ± 0.0	2.0 ± 0.4
2	128 ± 14	-0.06 ± 0.10	64.4 ± 8.1	11.1 ± 1.8	169.4 ± 22.9	48.9 ± 5.2	5.3 ± 0.8	1.2 ± 1.8	1.3 ± 0.2
3	117 ± 8	-0.05 ± 0.17	60.4 ± 6.5	13.0 ± 2.7	178.9 ± 18.7	46.1 ± 6.6	5.6 ± 0.5	2.2 ± 2.1	1.2 ± 0.2
4	144 ± 12	-0.04 ± 0.17	66.3 ± 7.8	11.7 ± 1.5	142.2 ± 26.2	45.3 ± 5.2	5.0 ± 0.4	1.7 ± 1.8	1.6 ± 0.3
5	173 ± 22	-0.06 ± 0.10	61.7 ± 7.8	10.5 ± 1.5	96.0 ± 12.4	3.5 ± 4.0	3.7 ± 0.4	0.0 ± 0.0	2.1 ± 0.7
6	125 ± 17	-0.05 ± 0.14	60.7 ± 9.4	12.5 ± 1.9	184.7 ± 28.6	53.7 ± 6.5	5.3 ± 1.2	1.8 ± 2.4	1.4 ± 0.3
7	115 ± 8	-0.09 ± 0.11	62.6 ± 9.8	12.6 ± 1.8	177.6 ± 27.1	48.0 ± 6.0	4.8 ± 0.5	2.8 ± 2.6	1.2 ± 0.2
8	138 ± 13	-0.01 ± 0.08	64.7 ± 4.8	12.3 ± 1.5	150.5 ± 20.4	48.9 ± 3.3	4.6 ± 0.2	1.5 ± 1.8	1.5 ± 0.2
9	130 ± 15	-0.05 ± 0.12	69.6 ± 9.0	11.3 ± 2.0	127.2 ± 15.1	22.2 ± 4.5	4.3 ± 0.4	2.8 ± 2.8	1.3 ± 0.2
10	136 ± 14	-0.02 ± 0.14	67.0 ± 6.5	12.0 ± 1.6	156.7 ± 18.0	48.9 ± 3.9	4.9 ± 0.5	1.2 ± 1.8	1.4 ± 0.3
11	118 ± 13	-0.05 ± 0.10	59.6 ± 7.2	12.0 ± 2.2	175.6 ± 20.5	50.7 ± 5.6	5.5 ± 0.7	2.2 ± 2.4	1.2 ± 0.2
12	130 ± 11	-0.04 ± 0.11	63.7 ± 8.7	12.7 ± 1.4	166.5 ± 24.1	50.4 ± 5.2	5.0 ± 0.4	2.4 ± 2.3	1.4 ± 0.3
13	122 ± 9	-0.03 ± 0.08	65.5 ± 5.8	11.3 ± 1.4	168.3 ± 19.7	49.3 ± 3.6	5.2 ± 0.5	2.0 ± 1.9	1.2 ± 0.2
14	120 ± 10	-0.05 ± 0.10	65.8 ± 9.3	11.9 ± 0.8	170.7 ± 23.4	51.9 ± 4.0	4.7 ± 0.6	1.9 ± 2.2	1.2 ± 0.2
15	114 ± 15	-0.04 ± 0.09	70.7 ± 9.7	12.9 ± 1.8	170.1 ± 23.2	42.3 ± 4.6	4.7 ± 0.6	1.6 ± 2.0	1.0 ± 0.3
16	118 ± 13	-0.03 ± 0.05	68.5 ± 3.1	13.5 ± 1.5	172.2 ± 10.8	46.0 ± 5.4	4.8 ± 0.2	0.4 ± 0.9	1.1 ± 0.2
17	120 ± 8	-0.02 ± 0.08	64.8 ± 4.5	13.2 ± 1	178.0 ± 16.6	48.2 ± 3.2	5.1 ± 0.4	1.3 ± 1.5	1.2 ± 0.2
mean:	130 ± 17	-0.04 ± 0.02	64.7 ± 3.2	12 ± 2	157.3 ± 28.2	41.4 ± 16.4	4.9 ± 0.5	1.6 ± 0.8	1.4 ± 0.3
CV:	13%	-46%	5%	14%	18%	40%	9%	53%	21%

Supplementary Information 8 Pearson correlation matrix of semi-continuous perfusion culture data (DoE2) in CDM4NS0 (A.) or ActiPro (B.). *Data generated from the exponential or stationary phase are depicted in the upper or lower panels, respectively.*

A. CDM4NS0

mean of d0-3 (expon.)

	VCD [×10^6 c/mL]	Viability [%]	Cell Diameter [µm]	Cell Circularity	Titer [mg/L]	residual Gluc [mg/L]	residual Lac [mg/L]	residual Glu [mg/L]	residual Gln [mg/L]	residual NH4+ [mg/L]	µv [day-1]	ΔIVCD[×10^6 c×d/mL]	qP [pg/c/d]	qGluc cons. [pg/c/d]	qLac prod. [pg/c/d]	qGlu cons. [pg/c/d]	qGln prod. [pg/c/d]	qNH4+ prod. [pg/c/d]	start. Osm [mOsm/kg]	IVCD[×10^6 c×d]	pVCD [×10^6 c/mL]	start Gluc [mg/L]	start Glu [mg/L]	Start Gln [mg/L]	start NH4+ [mg/L]
CB1 level	-0.65	0.20	0.13	0.76	-0.44	0.73	0.50	0.40	0.34	0.33	-0.40	-0.74	0.47	-0.49	0.65	0.07	0.80	0.62	0.78	-0.41	0.23	0.71	0.37	-0.49	-0.56
CB3 level	-0.61	0.13	-0.22	0.63	-0.51	0.36	0.28	0.18	0.10	0.28	-0.64	-0.58	0.18	-0.23	0.36	-0.47	0.48	0.34	0.07	-0.90	-0.62	0.35	0.01	-0.46	-0.06
CB7a level	-0.66	0.63	0.04	0.67	-0.50	0.44	0.62	0.89	0.95	0.49	-0.51	-0.67	0.52	-0.32	0.70	0.04	0.80	0.63	0.55	-0.49	-0.36	0.39	0.91	-0.01	-0.48
CB7b level	-0.43	0.23	-0.42	0.71	-0.09	0.05	0.84	0.35	0.26	-0.03	-0.19	-0.48	0.55	0.29	0.87	-0.32	0.80	0.16	0.10	-0.66	-0.44	0.12	0.27	-0.67	-0.36
VCD [×10^6 c/mL]		-0.26	-0.56	-0.26	0.85	-0.87	-0.48	-0.73	-0.64	-0.83	0.91	0.99	-0.05	0.64	-0.61	0.22	-0.71	-0.91	-0.79	0.63	0.52	-0.86	-0.59	0.03	-0.13
Viability [%]	-0.26		0.01	0.42	-0.02	0.07	0.44	0.45	0.44	0.19	-0.25	-0.23	0.31	0.03	0.42	0.13	0.42	0.22	0.20	0.00	-0.25	0.09	0.48	0.04	-0.25
Cell Diameter [µm]	-0.56	0.01		-0.59	-0.61	0.78	0.11	0.54	0.59	0.75	-0.48	-0.56	-0.25	-0.68	0.15	0.02	0.19	0.75	0.82	0.15	0.03	0.78	0.50	0.49	0.15
Cell Circularity	-0.26	0.42	-0.59		-0.06	-0.09	0.48	0.23	0.10	-0.03	-0.27	-0.26	0.35	0.18	0.53	-0.20	0.56	0.05	-0.12	-0.67	-0.50	-0.10	0.17	-0.50	-0.15
Titer [mg/L]	0.85	-0.02	-0.61	-0.06		-0.87	-0.07	-0.57	-0.48	-0.86	0.81	0.82	0.45	0.87	-0.22	0.31	-0.42	-0.87	-0.71	0.53	0.46	-0.80	-0.41	-0.10	-0.33
residual Gluc [mg/L]	-0.87	0.07	0.78	-0.09	-0.87		0.23	0.60	0.56	0.88	-0.72	-0.88	-0.19	-0.86	0.36	-0.13	0.53	0.94	0.91	-0.32	-0.20	0.98	0.48	0.08	0.06
residual Lac [mg/L]	-0.48	0.44	0.11	0.48	-0.07	0.23		0.69	0.62	0.31	-0.36	-0.51	0.64	0.15	0.97	-0.04	0.79	0.41	0.43	-0.34	-0.29	0.33	0.66	-0.13	-0.24
residual Glu [mg/L]	-0.73	0.45	0.54	0.23	-0.57	0.60	0.69		0.96	0.78	-0.66	-0.71	0.09	-0.43	0.72	-0.04	0.67	0.81	0.76	-0.28	-0.34	0.59	0.95	0.40	0.01
residual Gln [mg/L]	-0.64	0.44	0.59	0.10	-0.48	0.56	0.62	0.96		0.71	-0.57	-0.64	0.10	-0.39	0.63	0.07	0.61	0.75	0.75	-0.13	-0.20	0.57	0.95	0.51	-0.07
residual NH4+ [mg/L]	-0.83	0.19	0.75	-0.03	-0.86	0.88	0.31	0.78	0.71		-0.79	-0.80	-0.30	-0.76	0.38	-0.17	0.45	0.96	0.84	-0.34	-0.39	0.86	0.66	0.33	0.33
µv [day-1]	0.91	-0.25	-0.48	-0.27	0.81	-0.72	-0.36	-0.66	-0.57	-0.79		0.84	0.09	0.59	-0.46	0.21	-0.50	-0.79	-0.63	0.65	0.60	-0.69	-0.53	-0.09	-0.35
ΔIVCD [×10^6 c×d/mL]	0.99	-0.23	-0.56	-0.26	0.82	-0.88	-0.51	-0.71	-0.64	-0.80	0.84		-0.13	0.62	-0.65	0.17	-0.75	-0.90	-0.82	0.60	0.43	-0.88	-0.59	0.07	-0.04
qP [pg/c/d]	-0.05	0.31	-0.25	0.35	0.45	-0.19	0.64	0.09	0.10	-0.30	0.09	-0.13		0.53	0.62	0.23	0.45	-0.13	0.00	-0.08	0.07	-0.08	0.17	-0.36	-0.52
qGluc cons. [pg/c/d]	0.64	0.03	-0.68	0.18	0.87	-0.86	0.15	-0.43	-0.39	-0.76	0.59	0.62	0.53		0.00	0.21	-0.22	-0.78	-0.73	0.18	0.10	-0.74	-0.31	-0.22	-0.09
qLac prod. [pg/c/d]	-0.61	0.42	0.15	0.53	-0.22	0.36	0.97	0.72	0.63	0.38	-0.46	-0.65	0.62	0.00		-0.08	0.88	0.52	0.53	-0.44	-0.34	0.43	0.67	-0.21	-0.28
qGlu cons. [pg/c/d]	0.22	0.13	0.02	-0.20	0.31	-0.13	-0.04	-0.04	0.07	-0.17	0.21	0.17	0.23	0.21	-0.08		-0.05	-0.13	0.10	0.51	0.49	-0.09	0.25	0.15	-0.29
qGln prod. [pg/c/d]	-0.71	0.42	0.19	0.56	-0.42	0.53	0.79	0.67	0.61	0.45	-0.50	-0.75	0.45	-0.22	0.88	-0.05		0.62	0.61	-0.52	-0.35	0.57	0.62	-0.36	-0.37
qNH4+ prod. [pg/c/d]	-0.91	0.22	0.75	0.05	-0.87	0.94	0.41	0.81	0.75	0.96	-0.79	-0.90	-0.13	-0.78	0.52	-0.13	0.62		0.93	-0.38	-0.33	0.92	0.70	0.20	0.10
start. Osm [mOsm/kg]	-0.79	0.20	0.82	-0.12	-0.71	0.91	0.43	0.76	0.75	0.84	-0.63	-0.82	0.00	-0.73	0.53	0.10	0.61	0.93		-0.11	-0.06	0.90	0.73	0.22	-0.14
IVCD [×10^6 c×d]	0.63	0.00	0.15	-0.67	0.53	-0.32	-0.34	-0.28	-0.13	-0.34	0.65	0.60	-0.08	0.18	-0.44	0.51	-0.52	-0.38	-0.11		0.78	-0.31	-0.08	0.42	-0.26
max. VCD [×10^6 c/mL]	0.52	-0.25	0.03	-0.50	0.46	-0.20	-0.29	-0.34	-0.20	-0.39	0.60	0.43	0.07	0.10	-0.34	0.49	-0.35	-0.33	-0.06	0.78		-0.18	-0.16	0.17	-0.48
start Gluc [mg/L]	-0.86	0.09	0.78	-0.10	-0.80	0.98	0.33	0.59	0.57	0.86	-0.69	-0.88	-0.08	-0.74	0.43	-0.09	0.57	0.92	0.90	-0.31	-0.18		0.49	0.04	0.05
start Glu [mg/L]	-0.59	0.48	0.50	0.17	-0.41	0.48	0.66	0.95	0.95	0.66	-0.53	-0.59	0.17	-0.31	0.67	0.25	0.62	0.70	0.73	-0.08	-0.16	0.49		0.46	-0.10
Start Gln [mg/L]	0.03	0.04	0.49	-0.50	-0.10	0.08	-0.13	0.40	0.51	0.33	-0.09	0.07	-0.36	-0.22	-0.21	0.15	-0.36	0.20	0.22	0.42	0.17	0.04	0.46		0.27
start NH4+ [mg/L]	-0.13	-0.25	0.15	-0.15	-0.33	0.06	-0.24	0.01	-0.07	0.33	-0.35	-0.04	-0.52	-0.09	-0.28	-0.29	-0.37	0.10	-0.14	-0.26	-0.48	0.05	-0.10	0.27	

mean of d5-11 (stat.)

	VCD [×10^6 c/mL]	Viability [%]	Cell Diameter [µm]	Cell Circularity	Titer [mg/L]	residual Gluc [mg/L]	residual Lac [mg/L]	residual Glu [mg/L]	residual Gln [mg/L]	residual NH4+ [mg/L]	µv [day-1]	ΔIVCD [×10^6 c×d/mL]	qP [pg/c/d]	qGluc cons. [pg/c/d]*	qLac prod. [pg/c/d]*	qGlu cons. [pg/c/d]	qGln prod. [pg/c/d]	qNH4+ prod. [pg/c/d]	start. Osm [mOsm/kg]	IVCD[×10^6 c×d]	pVCD [×10^6 c/mL]	start Gluc [mg/L]	start Glu [mg/L]	Start Gln [mg/L]	start NH4+ [mg/L]
CB1 level	-0.19	0.55	0.62	-0.18	0.36	0.60	0.80	-0.06	0.46	-0.15	0.45	-0.25	0.37	0.56	0.80	0.69	0.71	-0.01	0.78	-0.41	0.23	0.71	0.37	-0.49	-0.56
CB3 level	-0.84	0.27	0.29	0.15	0.48	0.10	0.47	-0.02	0.21	-0.65	0.14	-0.87	0.86	0.76	0.59	0.38	0.55	0.37	0.07	-0.90	-0.62	0.35	0.01	-0.46	-0.06
CB7a level	-0.28	0.45	0.44	0.11	0.34	0.23	0.58	0.53	0.83	-0.10	0.52	-0.35	0.37	0.43	0.60	0.69	0.78	0.12	0.55	-0.49	-0.36	0.39	0.91	-0.01	-0.48
CB7b level	-0.64	0.23	-0.05	0.52	0.13	-0.05	0.42	0.19	0.19	-0.48	-0.01	-0.65	0.51	0.57	0.54	0.56	0.61	-0.02	0.10	-0.66	-0.44	0.12	0.27	-0.67	-0.36
VCD [×10^6 c/mL]		0.17	0.20	-0.51	-0.10	0.14	-0.02	0.03	0.16	0.66	0.25	0.99	-0.67	-0.50	-0.18	-0.04	-0.20	0.00	0.32	0.89	0.67	0.10	0.23	0.54	-0.21
Viability [%]	0.17		0.82	-0.56	0.76	0.44	0.82	0.21	0.70	0.21	0.79	0.07	0.49	0.54	0.78	0.60	0.75	0.13	0.74	-0.17	-0.11	0.83	0.53	0.00	-0.09
Cell Diameter [µm]	0.20	0.82		-0.83	0.63	0.73	0.87	0.35	0.78	0.42	0.85	0.08	0.36	0.35	0.82	0.47	0.75	0.33	0.90	-0.21	-0.12	0.95	0.57	0.20	0.07
Cell Circularity	-0.51	-0.56	-0.83		-0.37	-0.62	-0.55	-0.19	-0.52	-0.57	-0.63	-0.43	0.01	-0.03	-0.45	-0.10	-0.31	-0.22	-0.70	-0.19	-0.15	-0.73	-0.31	-0.46	-0.20
Titer [mg/L]	-0.10	0.76	0.63	-0.37		0.43	0.60	0.05	0.51	0.10	0.62	-0.19	0.80	0.54	0.59	0.49	0.58	0.14	0.53	-0.37	-0.33	0.73	0.29	-0.03	0.25
residual Gluc [mg/L]	0.14	0.44	0.73	-0.62	0.43		0.52	0.50	0.59	0.62	0.73	0.03	0.24	-0.17	0.50	0.10	0.55	0.58	0.84	-0.23	-0.25	0.77	0.47	0.16	0.10
residual Lac [mg/L]	-0.02	0.82	0.87	-0.55	0.60	0.52		0.21	0.77	0.06	0.71	-0.13	0.47	0.62	0.99	0.76	0.89	0.12	0.82	-0.38	-0.13	0.89	0.58	-0.06	-0.16
residual Glu [mg/L]	0.03	0.21	0.35	-0.19	0.05	0.50	0.21		0.63	0.52	0.54	-0.05	0.01	-0.28	0.22	0.00	0.48	0.55	0.47	-0.20	-0.40	0.22	0.79	0.44	0.17
residual Gln [mg/L]	0.16	0.70	0.78	-0.52	0.51	0.59	0.77	0.63		0.33	0.80	0.05	0.29	0.24	0.73	0.63	0.87	0.26	0.84	-0.22	-0.21	0.74	0.90	0.45	-0.01
residual NH4+ [mg/L]	0.66	0.21	0.42	-0.57	0.10	0.62	0.06	0.52	0.33		0.51	0.59	-0.33	-0.61	-0.04	-0.21	0.04	0.66	0.54	0.43	0.16	0.30	0.44	0.50	0.01
µv [day-1]	0.25	0.79	0.85	-0.63	0.62	0.73	0.71	0.54	0.80	0.51		0.11	0.32	0.15	0.66	0.39	0.74	0.43	0.86	-0.19	-0.25	0.81	0.69	0.21	0.08
ΔIVCD [×10^6 c×d/mL]	0.99	0.07	0.08	-0.43	-0.19	0.03	-0.13	-0.05	0.05	0.59	0.11		-0.73	-0.53	-0.28	-0.10	-0.31	-0.07	0.20	0.95	0.72	-0.02	0.14	0.51	-0.24
qP [pg/c/d]	-0.67	0.49	0.36	0.01	0.80	0.24	0.47	0.01	0.29	-0.33	0.32	-0.73		0.72	0.57	0.39	0.56	0.10	0.21	-0.80	-0.61	0.50	0.07	-0.35	0.30
qGluc cons. [pg/c/d]	-0.50	0.54	0.35	-0.03	0.54	-0.17	0.62	-0.28	0.24	-0.61	0.15	-0.53	0.72		0.68	0.63	0.52	-0.31	0.11	-0.57	-0.26	0.42	0.02	-0.39	0.03
qLac prod. [pg/c/d]	-0.18	0.78	0.82	-0.45	0.59	0.50	0.99	0.22	0.73	-0.04	0.66	-0.28	0.57	0.68		0.75	0.90	0.13	0.76	-0.52	-0.23	0.86	0.54	-0.15	-0.13
qGlu cons. [pg/c/d]	-0.04	0.60	0.47	-0.10	0.49	0.10	0.76	0.00	0.63	-0.21	0.39	-0.10	0.39	0.63	0.75		0.73	-0.12	0.52	-0.25	-0.01	0.54	0.57	-0.05	-0.33
qGln prod. [pg/c/d]	-0.20	0.75	0.75	-0.31	0.58	0.55	0.89	0.48	0.87	0.04	0.74	-0.31	0.56	0.52	0.90	0.73		0.25	0.78	-0.55	-0.38	0.78	0.74	-0.03	-0.12
qNH4+ prod. [pg/c/d]	0.00	0.13	0.33	-0.22	0.14	0.58	0.12	0.55	0.26	0.66	0.43	-0.07	0.10	-0.31	0.13	-0.12	0.25		0.39	-0.18	-0.30	0.25	0.35	0.06	-0.03
start. Osm [mOsm/kg]	0.32	0.74	0.90	-0.70	0.53	0.84	0.82	0.47	0.84	0.54	0.86	0.20	0.21	0.11	0.76	0.52	0.78	0.39		-0.11	-0.06	0.90	0.73	0.22	-0.14
IVCD[×10^6 c×d]	0.89	-0.17	-0.21	-0.19	-0.37	-0.23	-0.38	-0.20	-0.22	0.43	-0.19	0.95	-0.80	-0.57	-0.52	-0.25	-0.55	-0.18	-0.11		0.78	-0.31	-0.08	0.42	-0.26
pVCD [×10^6 c/mL]	0.67	-0.11	-0.12	-0.15	-0.33	-0.25	-0.13	-0.40	-0.21	0.16	-0.25	0.72	-0.61	-0.26	-0.23	-0.01	-0.38	-0.30	-0.06	0.78		-0.18	-0.16	0.17	-0.48
start Gluc [mg/L]	0.10	0.83	0.95	-0.73	0.73	0.77	0.89	0.22	0.74	0.30	0.81	-0.02	0.50	0.42	0.86	0.54	0.78	0.25	0.90	-0.31	-0.18		0.49	0.04	0.05
start Glu [mg/L]	0.23	0.53	0.57	-0.31	0.29	0.47	0.58	0.79	0.90	0.44	0.69	0.14	0.07	0.02	0.54	0.57	0.74	0.35	0.73	-0.08	-0.16	0.49		0.46	-0.10
Start Gln [mg/L]	0.54	0.00	0.20	-0.46	-0.03	0.16	-0.06	0.44	0.45	0.50	0.21	0.51	-0.35	-0.39	-0.15	-0.05	-0.03	0.06	0.22	0.42	0.17	0.04	0.46		0.27
start NH4+ [mg/L]	-0.21	-0.09	0.07	-0.20	0.25	0.10	-0.16	0.17	-0.01	0.01	0.08	-0.24	0.30	0.03	-0.13	-0.33	-0.12	-0.03	-0.14	-0.26	-0.48	0.05	-0.10	0.27	

B. ActiPro

mean of d0-3 (expon.)

	VCD [×10^6 c/mL]	Viability [%]	Cell Diameter [µm]	Cell Circularity	Titer [mg/L]	residual Gluc [mg/L]	residual Lac [mg/L]	residual Glu [mg/L]	residual Gln [mg/L]	residual NH4+ [mg/L]	µv [day-1]	ΔIVCD [×10^6 c×d/mL]	qP [pg/c/d]	qGluc cons. [pg/c/d]	qLac prod. [pg/c/d]	qGlu cons. [pg/c/d]	qGln prod. [pg/c/d]	qNH4+ prod. [pg/c/d]	res. Osm [mOsm/kg]	start. Osm [mOsm/kg]	IVCD[×10^6 c×d]	start Gluc [mg/L]	start Glu [mg/L]	start NH4+ [mg/L]
CB1 level	-0.92	-0.64	0.83	-0.53	-0.89	0.89	0.50	0.74	-0.27	0.83	-0.58	-0.91	-0.32	-0.58	0.55	-0.63	-0.11	0.88	0.98	0.99	-0.26	0.85	0.95	-0.69
CB3 level	-0.06	0.00	0.43	-0.36	-0.09	0.43	0.01	0.38	0.14	0.21	-0.01	-0.10	-0.06	-0.34	-0.17	-0.47	0.28	0.30	0.08	0.07	0.18	0.45	0.27	-0.71
CB7b level	-0.09	-0.22	-0.05	-0.05	0.02	-0.02	0.14	0.24	0.27	0.11	0.01	-0.09	0.28	0.38	0.24	-0.40	0.30	0.13	0.09	0.00	-0.12	0.00	0.00	0.00
VCD [×10^6 c/mL]	1.00	0.68	-0.81	0.62	0.89	-0.84	-0.50	-0.75	0.27	-0.80	0.74	0.99	0.20	0.49	-0.59	0.63	0.12	-0.86	-0.94	-0.89	0.49	-0.79	-0.90	0.67
Viability [%]	0.68	1.00	-0.63	0.73	0.68	-0.57	-0.42	-0.64	0.10	-0.76	0.38	0.72	0.21	0.21	-0.56	0.59	0.01	-0.75	-0.67	-0.60	0.37	-0.51	-0.57	0.37
Cell Diameter [µm]	-0.81	-0.63	1.00	-0.81	-0.80	0.96	0.44	0.87	-0.15	0.79	-0.45	-0.84	-0.29	-0.47	0.47	-0.80	0.05	0.89	0.87	0.86	-0.22	0.95	0.91	-0.85
Cell Circularity	0.62	0.73	-0.81	1.00	0.51	-0.67	-0.51	-0.66	0.02	-0.68	0.17	0.70	-0.06	0.07	-0.52	0.62	-0.13	-0.72	-0.60	-0.58	0.33	-0.68	-0.58	0.55
Titer [mg/L]	0.89	0.68	-0.80	0.51	1.00	-0.86	-0.35	-0.80	0.29	-0.82	0.73	0.87	0.61	0.61	-0.51	0.70	0.13	-0.86	-0.90	-0.87	0.32	-0.79	-0.90	0.68
residual Gluc [mg/L]	-0.84	-0.57	0.96	-0.67	-0.86	1.00	0.41	0.89	-0.19	0.85	-0.52	-0.85	-0.38	-0.61	0.47	-0.82	0.02	0.93	0.91	0.91	-0.17	0.97	0.97	-0.91
residual Lac [mg/L]	-0.50	-0.42	0.44	-0.51	-0.35	0.41	1.00	0.23	-0.20	0.36	-0.29	-0.52	0.21	-0.04	0.50	-0.26	-0.05	0.40	0.50	0.56	-0.25	0.53	0.45	-0.34
residual Glu [mg/L]	-0.75	-0.64	0.87	-0.66	-0.80	0.89	0.23	1.00	-0.04	0.81	-0.46	-0.78	-0.38	-0.36	0.62	-0.96	0.13	0.89	0.80	0.74	-0.23	0.79	0.82	-0.74
residual Gln [mg/L]	0.27	0.10	-0.15	0.02	0.29	-0.19	-0.20	-0.04	1.00	-0.03	0.43	0.23	0.33	0.17	-0.11	0.00	0.97	-0.06	-0.28	-0.26	0.34	-0.20	-0.25	0.08
residual NH4+ [mg/L]	-0.80	-0.76	0.79	-0.68	-0.82	0.85	0.36	0.81	-0.03	1.00	-0.43	-0.83	-0.35	-0.52	0.51	-0.73	0.14	0.97	0.85	0.82	-0.22	0.78	0.84	-0.69
µv [day-1]	0.74	0.38	-0.45	0.17	0.73	-0.52	-0.29	-0.46	0.43	-0.43	1.00	0.66	0.45	0.47	-0.26	0.35	0.32	-0.47	-0.61	-0.54	0.53	-0.47	-0.61	0.42
ΔIVCD [×10^6 c×d/mL]	0.99	0.72	-0.84	0.70	0.87	-0.85	-0.52	-0.78	0.23	-0.83	0.66	1.00	0.16	0.46	-0.63	0.66	0.07	-0.89	-0.93	-0.89	0.48	-0.81	-0.89	0.68
qP [pg/c/d]	0.20	0.21	-0.29	-0.06	0.61	-0.38	0.21	-0.38	0.33	-0.35	0.45	0.16	1.00	0.51	0.04	0.33	0.26	-0.33	-0.31	-0.29	0.00	-0.29	-0.38	0.26
qGluc cons. [pg/c/d]	0.49	0.21	-0.47	0.07	0.61	-0.61	-0.04	-0.36	0.17	-0.52	0.47	0.46	0.51	1.00	0.06	0.26	0.07	-0.53	-0.53	-0.54	0.00	-0.54	-0.65	0.67
qLac prod. [pg/c/d]	-0.59	-0.56	0.47	-0.52	-0.51	0.47	0.50	0.62	-0.11	0.51	-0.26	-0.63	0.04	0.06	1.00	-0.57	-0.03	0.56	0.57	0.52	-0.27	0.37	0.45	-0.22
qGlu cons. [pg/c/d]	0.63	0.59	-0.80	0.62	0.70	-0.82	-0.26	-0.96	0.00	-0.73	0.35	0.66	0.33	0.26	-0.57	1.00	-0.18	-0.82	-0.71	-0.65	0.14	-0.76	-0.73	0.74
qGln prod. [pg/c/d]	0.12	0.01	0.05	-0.13	0.13	0.02	-0.05	0.13	0.97	0.14	0.32	0.07	0.26	0.07	-0.03	-0.18	1.00	0.12	-0.10	-0.08	0.35	0.02	-0.05	-0.14
qNH4+ prod. [pg/c/d]	-0.86	-0.75	0.89	-0.72	-0.86	0.93	0.40	0.89	-0.06	0.97	-0.47	-0.89	-0.33	-0.53	0.56	-0.82	0.12	1.00	0.91	0.88	-0.22	0.87	0.91	-0.79
res. Osm [mOsm/kg]	-0.94	-0.67	0.87	-0.60	-0.90	0.91	0.50	0.80	-0.28	0.85	-0.61	-0.93	-0.31	-0.53	0.57	-0.71	-0.10	0.91	1.00	0.98	-0.32	0.88	0.97	-0.72
start. Osm [mOsm/kg]	-0.89	-0.60	0.86	-0.58	-0.87	0.91	0.56	0.74	-0.26	0.82	-0.54	-0.89	-0.29	-0.54	0.52	-0.65	-0.08	0.88	0.98	1.00	-0.21	0.91	0.96	-0.73
IVCD [×10^6 c×d]	0.49	0.37	-0.22	0.33	0.32	-0.17	-0.25	-0.23	0.34	-0.22	0.53	0.48	0.00	0.00	-0.27	0.14	0.35	-0.22	-0.32	-0.21	1.00	-0.14	-0.24	-0.02
start Gluc [mg/L]	-0.79	-0.51	0.95	-0.68	-0.79	0.97	0.53	0.79	-0.20	0.78	-0.47	-0.81	-0.29	-0.54	0.37	-0.76	0.02	0.87	0.88	0.91	-0.14	1.00	0.95	-0.91
start Glu [mg/L]	-0.90	-0.57	0.91	-0.58	-0.90	0.97	0.45	0.82	-0.25	0.84	-0.61	-0.89	-0.38	-0.65	0.45	-0.73	-0.05	0.91	0.97	0.96	-0.24	0.95	1.00	-0.85
start NH4+ [mg/L]	0.67	0.37	-0.85	0.55	0.68	-0.91	-0.34	-0.74	0.08	-0.69	0.42	0.68	0.26	0.67	-0.22	0.74	-0.14	-0.79	-0.72	-0.73	-0.02	-0.91	-0.85	1.00

mean of d5-11 (stat.)

	VCD [×10^6 c/mL]	Viability [%]	Cell Diameter [µm]	Cell Circularity	Titer [mg/L]	residual Gluc [mg/L]	residual Lac [mg/L]	residual Glu [mg/L]	residual Gln [mg/L]	residual NH4+ [mg/L]	µv [day-1]	ΔIVCD [×10^6 c×d/mL]	qP [pg/c/d]	qGluc cons. [pg/c/d]*	qLac prod. [pg/c/d]*	qGlu cons. [pg/c/d]	qGln prod. [pg/c/d]	qNH4+ prod. [pg/c/d]	res. Osm [mOsm/kg]	start. Osm [mOsm/kg]	IVCD[×10^6 c×d]	start Gluc [mg/L]	start Glu [mg/L]	start NH4+ [mg/L]
CB1 level	0.18	0.26	0.69	-0.73	0.24	0.81	0.62	0.43	-0.06	-0.15	0.37	0.12	0.20	0.37	0.61	0.37	-0.04	-0.13	0.92	0.99	-0.26	0.85	0.95	-0.69
CB3 level	0.17	0.56	0.47	-0.48	0.50	0.33	0.42	0.01	0.54	-0.31	0.05	0.18	0.51	0.28	0.39	0.10	0.50	-0.28	-0.12	0.07	0.18	0.45	0.27	-0.71
CB7b level	-0.18	0.02	0.05	0.16	0.12	-0.04	0.07	0.61	0.06	-0.04	-0.28	-0.13	0.23	0.09	0.08	-0.47	0.09	0.02	0.19	0.00	-0.12	0.00	0.00	0.00
VCD [×10^6 c/mL]	1.00	0.39	0.09	-0.06	0.57	0.12	0.09	-0.12	-0.02	-0.08	0.48	0.99	0.08	-0.12	-0.04	-0.29	-0.13	-0.26	0.07	0.22	0.85	0.20	0.17	-0.33
Viability [%]	0.39	1.00	0.66	-0.42	0.77	0.11	0.78	-0.21	0.60	-0.77	0.04	0.42	0.70	0.69	0.73	0.29	0.55	-0.79	0.10	0.40	0.36	0.62	0.39	-0.61
Cell Diameter [µm]	0.09	0.66	1.00	-0.87	0.50	0.54	0.92	0.14	0.42	-0.63	0.05	0.09	0.57	0.78	0.91	0.55	0.44	-0.57	0.53	0.78	-0.14	0.92	0.83	-0.82
Cell Circularity	-0.06	-0.42	-0.87	1.00	-0.28	-0.77	-0.69	-0.28	-0.21	0.28	-0.15	-0.03	-0.33	-0.49	-0.69	-0.49	-0.23	0.22	-0.59	-0.77	0.24	-0.88	-0.88	0.85
Titer [mg/L]	0.57	0.77	0.50	-0.28	1.00	0.03	0.65	-0.16	0.27	-0.67	0.24	0.57	0.86	0.60	0.57	0.15	0.23	-0.72	0.00	0.35	0.47	0.54	0.37	-0.57
residual Gluc [mg/L]	0.12	0.11	0.54	-0.77	0.03	1.00	0.35	0.60	0.01	0.19	0.33	0.06	0.00	0.02	0.34	0.14	0.00	0.22	0.81	0.76	-0.26	0.74	0.84	-0.78
residual Lac [mg/L]	0.09	0.78	0.92	-0.69	0.65	0.35	1.00	-0.05	0.49	-0.82	0.08	0.09	0.74	0.91	0.99	0.64	0.52	-0.77	0.43	0.73	-0.11	0.88	0.73	-0.72
residual Glu [mg/L]	-0.12	-0.21	0.14	-0.28	-0.16	0.60	-0.05	1.00	-0.29	0.47	-0.03	-0.13	-0.14	-0.25	-0.05	-0.48	-0.27	0.53	0.62	0.35	-0.29	0.24	0.42	-0.31
residual Gln [mg/L]	-0.02	0.60	0.42	-0.21	0.27	0.01	0.49	-0.29	1.00	-0.64	-0.33	0.05	0.36	0.50	0.51	0.32	0.99	-0.62	-0.04	0.05	0.14	0.32	0.06	-0.30
residual NH4+ [mg/L]	-0.08	-0.77	-0.63	0.28	-0.67	0.19	-0.82	0.47	-0.64	1.00	0.14	-0.12	-0.77	-0.92	-0.81	-0.61	-0.66	0.98	0.05	-0.30	-0.12	-0.50	-0.25	0.32
µv [day-1]	0.48	0.04	0.05	-0.15	0.24	0.33	0.08	-0.03	-0.33	0.14	1.00	0.33	0.05	-0.10	0.03	0.10	-0.39	0.07	0.24	0.34	0.07	0.25	0.33	-0.34
ΔIVCD [×10^6 c×d/mL]	0.99	0.42	0.09	-0.03	0.57	0.06	0.09	-0.13	0.05	-0.12	0.33	1.00	0.09	-0.10	-0.04	-0.33	-0.05	-0.30	0.02	0.16	0.91	0.17	0.12	-0.29
qP [pg/c/d]	0.08	0.70	0.57	-0.33	0.86	0.00	0.74	-0.14	0.36	-0.77	0.05	0.09	1.00	0.80	0.72	0.40	0.37	-0.72	-0.02	0.32	0.03	0.55	0.37	-0.51
qGluc cons. [pg/c/d]	-0.12	0.69	0.78	-0.49	0.60	0.02	0.91	-0.25	0.50	-0.92	-0.10	-0.10	0.80	1.00	0.93	0.72	0.55	-0.85	0.17	0.50	-0.19	0.66	0.48	-0.44
qLac prod. [pg/c/d]	-0.04	0.73	0.91	-0.69	0.57	0.34	0.99	-0.05	0.51	-0.81	0.03	-0.04	0.72	0.93	1.00	0.70	0.55	-0.74	0.42	0.71	-0.22	0.86	0.71	-0.68
qGlu cons. [pg/c/d]	-0.29	0.29	0.55	-0.49	0.15	0.14	0.64	-0.48	0.32	-0.61	0.10	-0.33	0.40	0.72	0.70	1.00	0.38	-0.53	0.15	0.44	-0.45	0.51	0.42	-0.28
qGln prod. [pg/c/d]	-0.13	0.55	0.44	-0.23	0.23	0.00	0.52	-0.27	0.99	-0.66	-0.39	-0.05	0.37	0.55	0.55	0.38	1.00	-0.61	-0.02	0.07	0.04	0.33	0.08	-0.28
qNH4+ prod. [pg/c/d]	-0.26	-0.79	-0.57	0.22	-0.72	0.22	-0.77	0.53	-0.62	0.98	0.07	-0.30	-0.72	-0.85	-0.74	-0.53	-0.61	1.00	0.07	-0.28	-0.29	-0.46	-0.21	0.30
res. Osm [mOsm/kg]	0.07	0.10	0.53	-0.59	0.00	0.81	0.43	0.62	-0.04	0.05	0.24	0.02	-0.02	0.17	0.42	0.15	-0.02	0.07	1.00	0.88	-0.32	0.70	0.83	-0.53
start. Osm [mOsm/kg]	0.22	0.40	0.78	-0.77	0.35	0.76	0.73	0.35	0.05	-0.30	0.34	0.16	0.32	0.50	0.71	0.44	0.07	-0.28	0.88	1.00	-0.21	0.91	0.96	-0.73
IVCD [×10^6 c×d]	0.85	0.36	-0.14	0.24	0.47	-0.26	-0.11	-0.29	0.14	-0.12	0.07	0.91	0.03	-0.19	-0.22	-0.45	0.04	-0.29	-0.32	-0.21	1.00	-0.14	-0.24	-0.02
start Gluc [mg/L]	0.20	0.62	0.92	-0.88	0.54	0.74	0.88	0.24	0.32	-0.50	0.25	0.17	0.55	0.66	0.86	0.51	0.33	-0.46	0.70	0.91	-0.14	1.00	0.95	-0.91
start Glu [mg/L]	0.17	0.39	0.83	-0.88	0.37	0.84	0.73	0.42	0.06	-0.25	0.33	0.12	0.37	0.48	0.71	0.42	0.08	-0.21	0.83	0.96	-0.24	0.95	1.00	-0.85
start NH4+ [mg/L]	-0.33	-0.61	-0.82	0.85	-0.57	-0.78	-0.72	-0.31	-0.30	0.32	-0.34	-0.29	-0.51	-0.44	-0.68	-0.28	-0.28	0.30	-0.53	-0.73	-0.02	-0.91	-0.85	1.00

Supplementary Information 9 PLS regression model qualities generated from DoE2 semi-continuous perfusion models for CDM4NS (A.) or ActiPro (B.).

A. CDM4NS0

	R^2	Q^2
mVCD_stat	0.908735	0.749029
mViability_stat~	0.934508	0.759554
mDiam_stat	0.993466	0.707411
mTiter_stat	0.717332	0.568064
mResGluc_stat	0.954637	0.872409
mResLac_stat	0.966822	0.830138
mResGlu_stat	0.891112	0.687558
mResGln_stat	0.989198	0.79202
mResNH4+_stat	0.899665	0.613412
mµ_stat	0.156463	0.05
mDeltaIVCD_stat	0.93448	0.73118
mqP_stat~	0.845035	0.702786
mqGluc_stat	0.927084	0.744659
mqLac_stat~	0.963274	0.784082
mqGlu_stat~	0.768426	0.655032
mqGln_stat~	0.938532	0.785926
mqNH4+_stat	0.724333	0.244307
IVCD	0.932619	0.72053
pVCD	0.959979	0.743282
mVCD_exp	0.93582	0.85794
mViability_exp	0.595492	0.30431
mDiameter_exp	0.953204	0.757249
mTiter_exp	0.935435	0.800854
mResGluc_exp	0.993921	0.805512
mResLac_exp	0.934673	0.701697
mResGlu_exp	0.989519	0.907394
mResGln_exp	0.996311	0.910789
mResNH4+_exp	0.98443	0.841261
mµ_exp	0.646966	0.600035
mDeltaIVCD_exp	0.960524	0.833402
mqP_exp	0.878431	0.60518
mqGluc_exp	0.86888	0.722059
mqLac_exp	0.986535	0.912713
mqGlu_exp	0.214901	0.18173
mqGln_exp	0.881059	0.793632
mqNH4+_exp	0.981723	0.896656
startOsm	0.992399	0.924549
startGluc	0.99521	0.937325
startGlu	0.968584	0.890533
startGln	0.782324	0.617386
startNH4+	0.961184	0.773351
mTiter	0.742086	0.617812
pTiter	0.708445	0.558996

B. ActiPro

	R^2	Q^2
mVCD_stat	0.741919	0.560084
mViability_stat~	0.823211	0.676639
mDiameter_stat~	0.970021	0.764105
mTiter_stat	0.791559	0.662078
mResGluc_stat~	0.805661	0.674798
mResLac_stat	0.958226	0.8543
mResGlu_stat	0.828964	0.577224
mResGln_stat	0.469254	0.287767
mResNH4+_stat~	0.863958	0.75029
mµ_stat	0.753463	0.465009
mDeltaIVCD_stat	0.704291	0.468912
mqP_stat	0.737085	0.569698
mqGluc_stat	0.934877	0.879173
mqLac_stat~	0.924271	0.877781
mqGlu_stat	0.879621	0.465865
mqGln_stat	0.504617	0.35743
mqNH4+_stat~	0.849125	0.696966
mresOsm_stat	0.921633	0.864193
startOsm	0.998385	0.922544
IVCD	0.723516	0.541215
pVCD	0.312254	0.134588
startGluc	0.997275	0.894626
startGlu	0.980299	0.972475
startNH4+	0.985971	0.968557
mVCD_exp	0.889308	0.855908
mViability_exp	0.679134	0.474126
mDiameter_exp	0.924818	0.813947
mTiter_exp	0.889484	0.840384
mResGluc_exp	0.978539	0.93775
mResLac_exp	0.252071	0.159244
mResGlu_exp	0.746525	0.707259
mResGln_exp	0.35737	0.113
mResNH4+_exp~	0.829826	0.763227
mµ_exp	0.57316	0.479736
mdeltaIVCD_exp	0.882684	0.833753
mqP_exp	0.539596	0.351023
mqGluc_exp	0.74037	0.689002
mqLac_exp	0.559283	0.272042
mqGlu_exp	0.783561	0.743515
mqGln_exp	0.426574	0.178688
mqNH4+_exp	0.903294	0.860277
mResOsm_exp	0.99359	0.822687

Supplementary Information 10 PLS regression coefficients for DoE2 semi-continuous perfusion cultures in CDM4NS0 (A.) or ActiPro (B.). *'~' represents transformed data sets.*

A. CDM4NS0

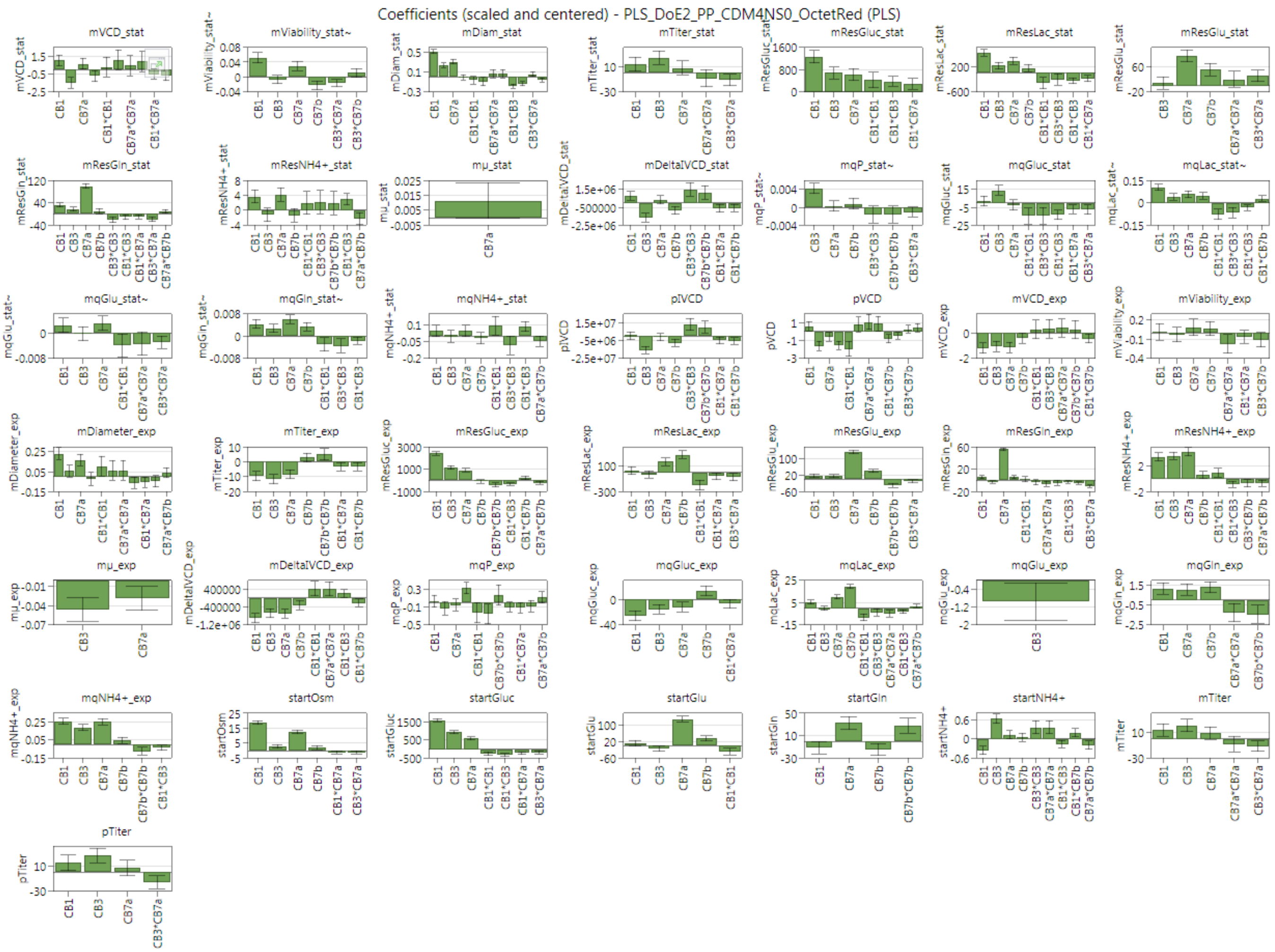

B. ActiPro

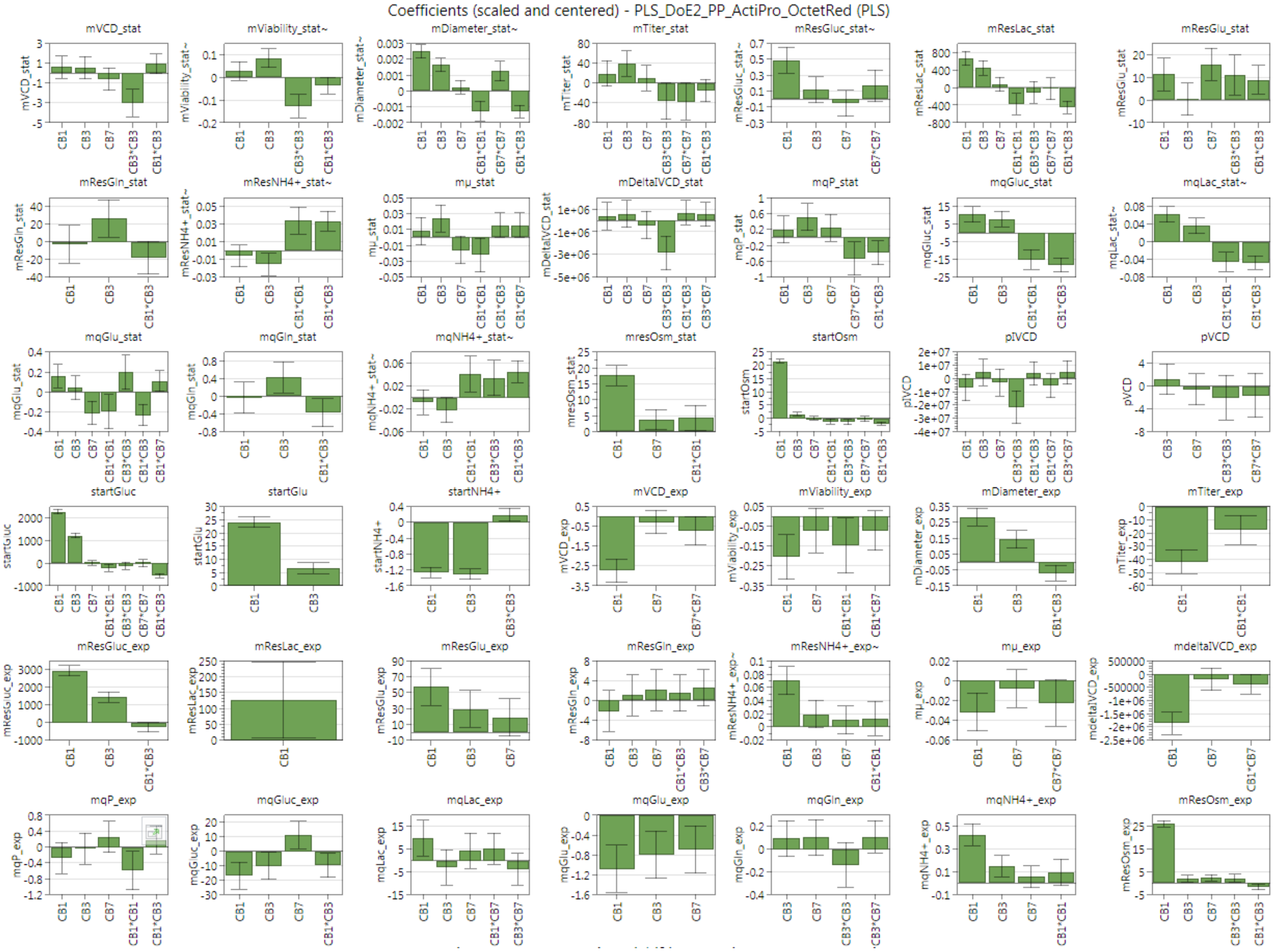
Coefficients (scaled and centered) - PLS_DoE2_PP_ActiPro_OctetRed (PLS)

Supplementary Information 11 Metabolite concentrations and specific consumption- or production rates of semi-continuous perfusion cultures for media comparison.

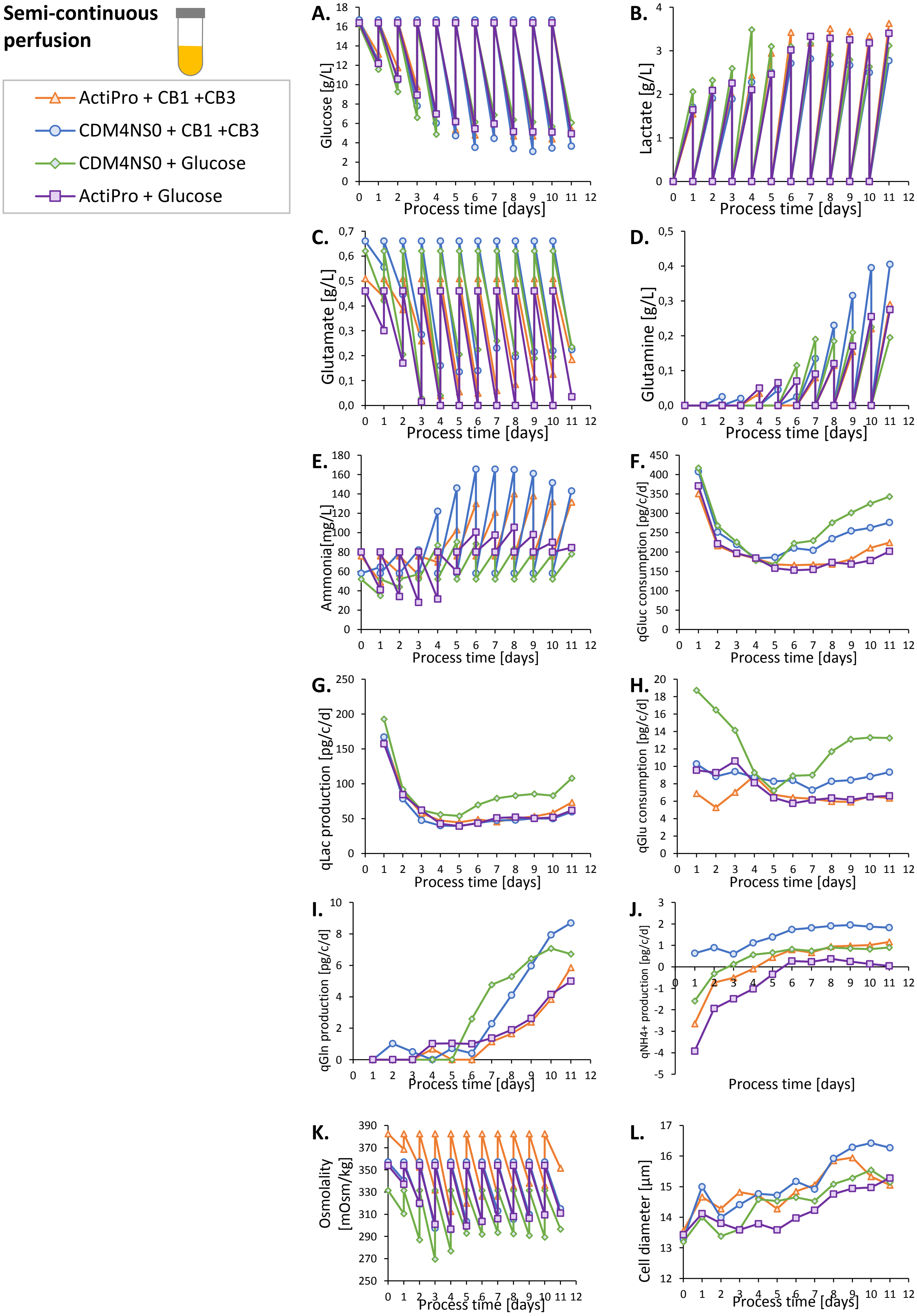

Supplementary Information 12 Comparison of glucose-spiked and CB1 + CB3-spiked CDM4NS0 and ActiPro small-scale semi-continuous perfusion cultures in the exponential or stationary phase.

	Exponential phase (mean of day 0-4)			Stationary phase (mean of day 5-11)		
media	CDM4NS0 + Gluc	CDM4NS0 + CB1 + CB3	ratio	CDM4NS0 + Gluc	CDM4NS0 + CB1 + CB3	ratio
Total cell count [×10^6 c/mL]	42 ± 26	41 ± 25	1.0	51 ± 5	62 ± 5	1.2
Viability [%]	96 ± 7	98 ± 1	1.0	72 ± 6	91 ± 4	1.3
Titer [g/L]	0.29 ± 0.19	0.35 ± 0.23	1.2	0.32 ± 0.04	0.50 ± 0.04	1.6
specific growth rate µ [1/d]	0.42 ± 0.57	0.50 ± 0.42	1.2	-0.11 ± 0.16	-0.02 ± 0.03	0.2
specific productivity qP [pg/c/d]	11.12 ± 5.25	12.28 ± 3.31	1.1	8.97 ± 2.89	9.06 ± 0.86	1.0
Cell diameter [µm]	13.9 ± 0.6	14.4 ± 0.6	1.0	15.0 ± 0.4	15.7 ± 0.7	1.0
Osmolality [mOsm/kg]	295 ± 23	319 ± 25	1.1	293 ± 2	309 ± 5	1.1
Glucose [g/L]	9.1 ± 4.2	9.7 ± 4.4	1.1	6.2 ± 0.4	3.8 ± 0.6	0.6
Lactate [g/L]	2.3 ± 1.2	1.7 ± 0.9	0.8	3.0 ± 0.2	2.7 ± 0.1	0.9
Glutamate [g/L]	0.25 ± 0.23	0.37 ± 0.21	1.5	0.22 ± 0.02	0.19 ± 0.04	0.9
Glutamine [g/L]	0.00 ± 0.00	0.02 ± 0.02	n.a.	0.16 ± 0.08	0.22 ± 0.16	1.4
Ammonia [mg/L]	61 ± 23	92 ± 35	1.5	83 ± 5	157 ± 10	1.9
specific glucose consumption qGluc [pg/c/d]	251 ± 101	250 ± 93	1.0	266 ± 63	233 ± 34	0.9
specific lactate production qLac [pg/c/d]	91.4 ± 58.8	74.5 ± 54.1	0.8	80.3 ± 16.4	48.4 ± 6.2	0.6
specific glutamate consumption qGlu [pg/c/d]	13.2 ± 4.8	9.1 ± 0.8	0.7	10.9 ± 2.5	8.4 ± 0.6	0.8
specific glutamine production qGln [pg/c/d]	0.0 ± 0.0	0.4 ± 0.4	n.a.	4.7 ± 2.6	4.3 ± 3.4	0.9
specific ammonia production qNH4+ [pg/c/d]	-0.1 ± 0.9	0.9 ± 0.3	-8.3	0.8 ± 0.1	1.8 ± 0.2	2.2
max. IVCD [×10^9 c×d]	1.40 ± 0.00	1.32 ± 0.00	0.9	4.10 ± 0.09	5.22 ± 0.05	1.3

	Exponential phase (mean of day 0-4)			Stationary phase (mean of day 5-11)		
media	ActiPro + Gluc	ActiPro + CB1 + CB3	ratio	ActiPro + Gluc	ActiPro + CB1 + CB3	ratio
Total cell count [×10^6 c/mL]	40 ± 24	40 ± 27	1.0	67 ± 6	69 ± 9	1.0
Viability [%]	99 ± 1	98 ± 1	1.0	97 ± 1	95 ± 3	1.0
Titer [g/L]	0.37 ± 0.24	0.42 ± 0.31	1.1	0.50 ± 0.03	0.61 ± 0.06	1.2
specific growth rate µ [1/d]	0.53 ± 0.42	0.54 ± 0.35	1.0	0.02 ± 0.12	-0.01 ± 0.11	-0.5
specific productivity qP [pg/c/d]	13.67 ± 3.73	15.20 ± 3.16	1.1	7.93 ± 0.70	9.56 ± 0.77	1.2
Cell diameter [µm]	13.7 ± 0.2	14.4 ± 0.5	1.0	14.5 ± 0.6	15.2 ± 0.6	1.0
Osmolality [mOsm/kg]	318 ± 23	345 ± 28	1.1	306 ± 4	333 ± 10	1.1
Glucose [g/L]	10.2 ± 3.7	10.6 ± 4.1	1.0	5.4 ± 0.5	4.9 ± 0.4	0.9
Lactate [g/L]	1.8 ± 0.9	1.8 ± 1.0	1.0	3.1 ± 0.3	3.4 ± 0.2	1.1
Glutamate [g/L]	0.16 ± 0.19	0.28 ± 0.20	1.8	0.01 ± 0.01	0.10 ± 0.05	19.3
Glutamine [g/L]	0.02 ± 0.03	0.01 ± 0.01	0.3	0.15 ± 0.09	0.12 ± 0.11	0.8
Ammonia [mg/L]	46 ± 20	69 ± 19	1.5	91 ± 15	128 ± 13	1.4
specific glucose consumption qGluc [pg/c/d]	226 ± 84	223 ± 74	1.0	170 ± 17	184 ± 24	1.1
specific lactate production qLac [pg/c/d]	77.2 ± 48.2	80.4 ± 49.9	1.0	50.0 ± 7.1	53.4 ± 9.9	1.1
specific glutamate consumption qGlu [pg/c/d]	8.8 ± 1.6	7.0 ± 1.3	0.8	6.3 ± 0.3	6.3 ± 0.3	1.0
specific glutamine production qGln [pg/c/d]	0.0 ± 0.6	0.1 ± 0.3	n.a.	2.4 ± 1.6	2.1 ± 2.1	0.9
specific ammonia production qNH4+ [pg/c/d]	-1.7 ± 1.4	-0.7 ± 1.2	0.4	0.1 ± 0.2	0.9 ± 0.2	6.3
max. IVCD [×10^9 c×d]	1.21 ± 0.05	1.17 ± 0.00	1.0	5.62 ± 0.06	5.65 ± 0.10	1.0

Supplementary Information 13 Metabolite concentrations and specific consumption- or production rates of bioreactor perfusion cultures for media comparison.

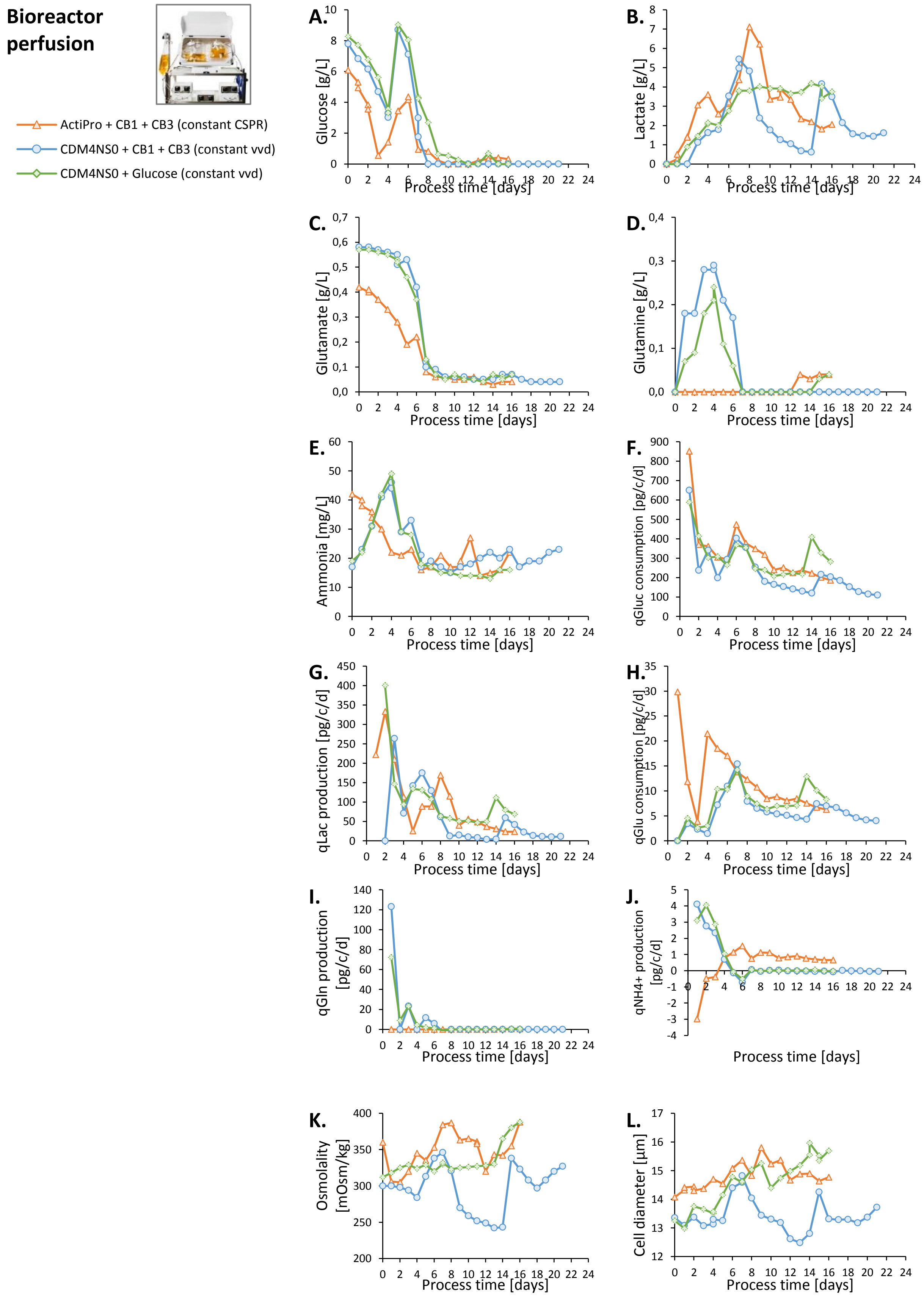

Supplement: Supplementary file 1 — Supporting Information [file BTPR-36-e2933-s001.pdf]
